# Src is activated by the nuclear receptor peroxisome proliferator-activated receptor β/δ in ultraviolet radiation-induced skin cancer

**DOI:** 10.1002/emmm.201302666

**Published:** 2013-11-06

**Authors:** Alexandra Montagner, Maria B Delgado, Corinne Tallichet-Blanc, Jeremy S K Chan, Ming K Sng, Hélène Mottaz, Gwendoline Degueurce, Yannick Lippi, Catherine Moret, Michael Baruchet, Maria Antsiferova, Sabine Werner, Daniel Hohl, Talal Al Saati, Pierre J Farmer, Nguan S Tan, Liliane Michalik, Walter Wahli

**Affiliations:** 1 Center for Integrative Genomics, National Research Center Frontiers in Genetics, University of LausanneLe Genopode, Lausanne, Switzerland; 2 School of Biological Sciences, Nanyang Technological UniversityNanyang Drive, Singapore, Singapore; 3 GeT-TRiX Facility, INRA ToxAlim, UMR1331Chemin de Tournefeuille, Toulouse Cedex, France; 4 Department of Biology, Institute of Molecular Health Sciences, ETH ZurichSchafmattstrasse, Zurich, Switzerland; 5 Department of Dermatology, University Hospital of Lausanne (CHUV)Lausanne, Switzerland; 6 INSERM/UPS, US006/CREFRE, Histopathology Facility, Place du Docteur BaylacCHU Purpan, Toulouse Cedex, France; 7 Exploratory Biomarker Analysis, Biomarker Technologies, Bioinformatics, Non Clinical Development, Merck Serono International S.A. SwitzerlandChemin des Mines, Geneva, Switzerland; 8 Institute of Molecular and Cell Biology, Biopolis DriveProteos, Singapore, Singapore; 9 Lee Kong Chian School of Medicine, Imperial College London, Nanyang Technological UniversitySingapore, Singapore

**Keywords:** keratinocyte, PPAR beta/delta, Skin cancer, Src, UV

## Abstract

Although non-melanoma skin cancer (NMSC) is the most common human cancer and its incidence continues to rise worldwide, the mechanisms underlying its development remain incompletely understood. Here, we unveil a cascade of events involving peroxisome proliferator-activated receptor (PPAR) β/δ and the oncogene *Src*, which promotes the development of ultraviolet (UV)-induced skin cancer in mice. UV-induced PPARβ/δ activity, which directly stimulated *Src* expression, increased Src kinase activity and enhanced the EGFR/Erk1/2 signalling pathway, resulting in increased epithelial-to-mesenchymal transition (EMT) marker expression. Consistent with these observations, PPARβ/δ-null mice developed fewer and smaller skin tumours, and a PPARβ/δ antagonist prevented UV-dependent *Src* stimulation. Furthermore, the expression of *PPAR*β*/*δ positively correlated with the expression of *SRC* and EMT markers in human skin squamous cell carcinoma (SCC), and critically, linear models applied to several human epithelial cancers revealed an interaction between PPARβ/δ and SRC and TGFβ1 transcriptional levels. Taken together, these observations motivate the future evaluation of PPARβ/δ modulators to attenuate the development of several epithelial cancers.

## Introduction

The incidence of non-melanoma skin cancer (NMSC) is increasing at a high rate (3–8% yearly), with over one million estimated new cases each year in the United States alone (Madan *et al*, 2010). Squamous cell carcinoma (SCC) and basal cell carcinoma, which both derive from keratinocytes, are the most common types of NMSC. Although mortality due to NMSC is low, its morbidity is high and is accompanied by heavy personal burden and enormous, rising costs for society. NMSCs are associated with excessive and/or chronic exposure to ultraviolet (UV) radiation and mainly occur on sun-exposed areas. In particular, UVB radiation (290–320 nm, sunburn rays) predominantly affects keratinocytes because of its low skin penetration and is believed to act as a carcinogen (Ehrhart *et al*, 2003). Despite the prevalence of NMSCs, the mechanisms underlying their progression remain poorly defined.

Tumours have often been referred to as ‘wounds that never heal’. Gene expression profiling, which has revealed that a wound-response gene expression pattern predicts metastasis, survival likelihood and response to chemotherapy in cancer patients, supports this concept (Bissell & Radisky, 2001; Chang *et al*, 2004; Farmer *et al*, 2009; Nuyten & van de Vijver, 2006; Schafer & Werner, 2008). Apoptosis resistance, cell proliferation and migration, matrix remodelling, and angiogenesis are processes of life-saving importance after an injury but are also common features of tumour progression. The identification of molecular regulators of the mechanisms that activate, sustain and eventually shut down these processes may advance our understanding of tumour development in general and of NMSC in particular.

Our previous work on skin wound healing in mice demonstrated the importance of the peroxisome proliferator-activated receptors (PPARs), members of the nuclear receptor superfamily, in tissue repair (Michalik *et al*, 2001). Injury strongly stimulates expression of PPARα (NR1C1) and PPARβ/δ (NR1C2) in keratinocytes at the edges of skin wounds, and individual genetic deletion of these isotypes delays healing (Michalik *et al*, 2001). Both PPARα and PPARβ/δ affect the early inflammatory reaction, but PPARβ/δ-null mice exhibit additional defects in healing resulting from impaired keratinocyte adhesion and migration, as well as increased apoptosis at wound edges (Di-Poi *et al*, 2002; Michalik *et al*, 2005; Tan *et al*, 2001; Tan *et al*, 2007). Thus, during wound healing, inflammation-induced PPARβ/δ controls processes that are also involved in carcinogenesis. Whether PPARβ/δ is pro- or anti-carcinogenic remains controversial, however, because both effects have been observed (Michalik *et al*, 2004; Muller-Brusselbach *et al*, 2007; Peters & Gonzalez, 2009; Schug *et al*, 2007; Schug *et al*, 2008). To address this question, we investigated PPARβ/δ function in keratinocyte-derived UV-induced skin tumours. Here, we unveil a previously unrecognized control by PPARβ/δ over a pro-oncogenic pathway, identifying PPARβ/δ as a key focus for further epithelial cancer studies.

## Results

### UV radiation stimulates PPARβ/δ expression and activity, enhancing skin tumour formation and progression *in vivo*

**Figure 1:**
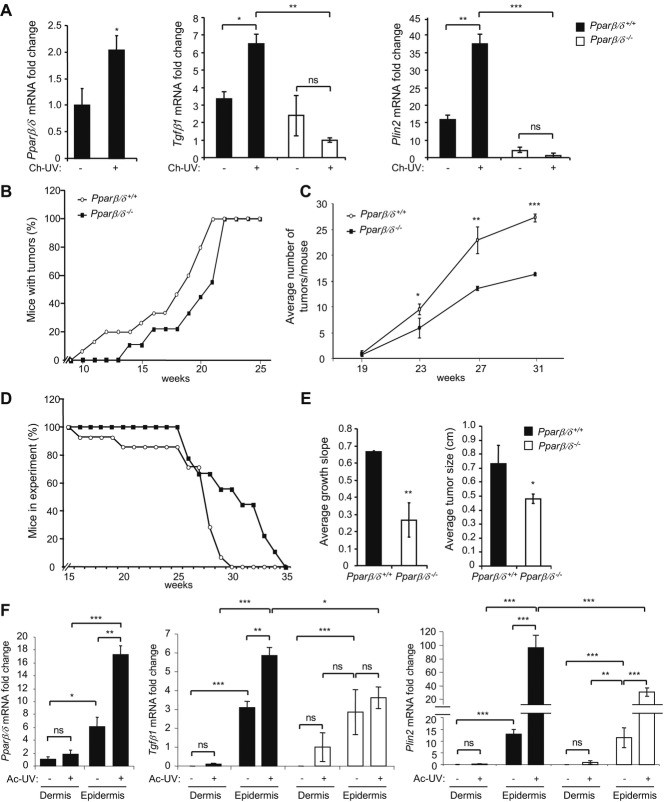
PPARβ/´ is over-expressed and activated upon UV radiation and enhances skin tumour formation and growth. A  Quantification of *Ppar*β/δ (left), *Tgf*β*1* (middle) and *Plin2* (right) expression by RT-PCR in non-tumoural dorsal skin of chronically irradiated (Ch-UV, +) or non-irradiated (−) *Ppar*β/δ^*+/+*^ and *Ppar*β/δ^*−/−*^ mice. Values from non-irradiated dorsal skin of *Ppar*β/δ^*+/+*^ mice (left) or from irradiated dorsal skin of *Ppar*β/δ^*−/−*^ mice (right) were set to 1. Means ± SEM of 13 *Ppar*β/δ^*+/+*^ and 9 *Ppar*β/δ^*−/−*^ mice are presented. *p*-values are *p = 0.035 for *Ppar*β/δ (left); *p = 0.025 and **p = 0.008 for *Tgf*β*1*(middle); **p = 0.078 and ***p = 0.0003 for *Plin2* (right) calculated by two-tailed Student's *t*-test. B,C  Percentage of *Ppar*β/δ^*+/+*^ and *Ppar*β/δ^*−/−*^ mice bearing tumours (B) and average number of tumours per mouse (C) measured at the indicated time points of UV exposure. For (C), *p*-values are *p = 0.025, **p = 0.008, ***p = 0.0001 calculated by two-tailed Student's *t*-test. Data for 13 *Ppar*β/δ^*+/+*^ and 9 *Ppar*β/δ^*−/−*^ mice are presented. D  Percentage of *Ppar*β/δ^*+/+*^ and *Ppar*β/δ^*−/−*^ mice involved in the experiment over time. Mice were withdrawn from the experiment based on criteria given in the Material and Methods section. E  Tumour growth rate (left) and final size (right) from *Ppar*β/δ^*+/+*^ and *Ppar*β/δ^*−/−*^ mice measured before sacrifice. Means ± SEM for 13 *Ppar*β/δ^*+/+*^ and 9 *Ppar*β/δ^*−/−*^ mice are presented. *p*-values are **p = 0.009 for tumour growth rate (left) and *p = 0.045 for tumour size (right) calculated by two-tailed Student's *t*-test. F  Measurement of *Ppar*β/δ (left), *Tgf*β*1* (middle) and *Plin2* (right) expression by RT-PCR in dermis and epidermis compartments of dorsal skin of *Ppar*β/δ^*+/+*^ and *Ppar*β/δ^*−/−*^ mice 24 h after acute UV (Ac-UV) irradiation. Values from dermis of non-irradiated dorsal skin of *Ppar*β/δ^*+/+*^ mice (left) or from dermis of Ac-UV dorsal skin of *Ppar*β/δ^*−/−*^ mice (middle and right) were set to 1. Means ± SEM for 12 *Ppar*β/δ^*+/+*^and 12 *Ppar*β/δ^*−/−*^ mice are presented. Values are representative of three independent experiments; *p*-values are *p = 0.025, **p = 0.007, ***p = 0.002 for *Ppar*β/δ; ***p = 0.0003, *p = 0.049, **p = 0.0093, ***p = 0.0041, ***p = 0.0004 from top to bottom for *Tgf*β*1*; ***p = 0.0048, ***p = 0.0013, ***p = 0.0006, ***p = 0.0009, **p = 0.0071, ***p = 0.0035, ***p = 0.0029 from top to bottom and from left to right for *Plin2* calculated by two-tailed Student's *t*-test; ns, not significant.

To test if PPARβ/δ is implicated in UV-dependent tumour development, we first investigated whether its expression responds to UV radiation. SKH-1 hairless mice were exposed to chronic low-dose UV radiation for up to 31 weeks to mimic repeated exposure to sunlight. Interestingly, *Ppar*β/δ expression increased in dorsal skin upon chronic UV exposure, *Ppar*α expression was reduced and *Ppar*γ expression was unchanged (Fig 1A, left) (supplementary Fig S1). Additionally, the expression of *Tgf*β*1* and *Plin2*, two well-known PPARβ/δ target genes, was stimulated in wild-type but not in *Ppar*β/δ^−/−^ mice, indicating activation of the receptor (Fig 1A). Importantly, when wild-type and *Ppar*β*/*δ^−/−^SKH-1 hairless mice were subjected to chronic UV radiation, tumour incidence and multiplicity were much higher in wild-type mice compared to*Ppar*β*/*δ^−/−^ mice during the course of chronic UV exposure (Fig 1B). Wild-type mice fulfilled the criteria for withdrawal from the experiment (tumour larger than 9 mm in diameter) earlier than *Ppar*β*/*δ^−/−^ mice (Fig 1C, D). Eventually, a 100% tumour incidence was reached in both genotypes after 22 weeks of UV exposure, which might have resulted from gene mutations arising from long-term UV exposure and associated modifications in gene expression in the *Ppar*β*/*δ^−/−^ mice, which override the inhibitory effect of *Ppar*β*/*δ deficiency on tumourigenesis. Nevertheless, the growth rate of the tumours and their final size were higher in wild-type mice (Fig 1E).

To gain additional support for a *Ppar*β*/*δ-specific response to UV exposure, the SKH-1 mice were exposed to a single acute UV dose and sacrificed 24 h later. The UV treatment caused enhanced erythema in wild-type mice, characterized by an increased expression of inflammatory markers (supplementary Fig S2A). As with chronic irradiation, PPARβ/δ expression and activity were enhanced in the skin of wild-type animals upon acute UV exposure, especially in the epithelial compartment where carcinomas arise, as documented by the stimulation of two known target genes, *Tgf*β*1* and *Plin2* (Fig 1F) (supplementary Fig S2B). These observations suggest a major function for PPARβ/δ in the initiation and progression of skin tumours in response to UV irradiation.

### Increased levels of activated PPARβ/δ enhance Src expression

**Figure 2:**
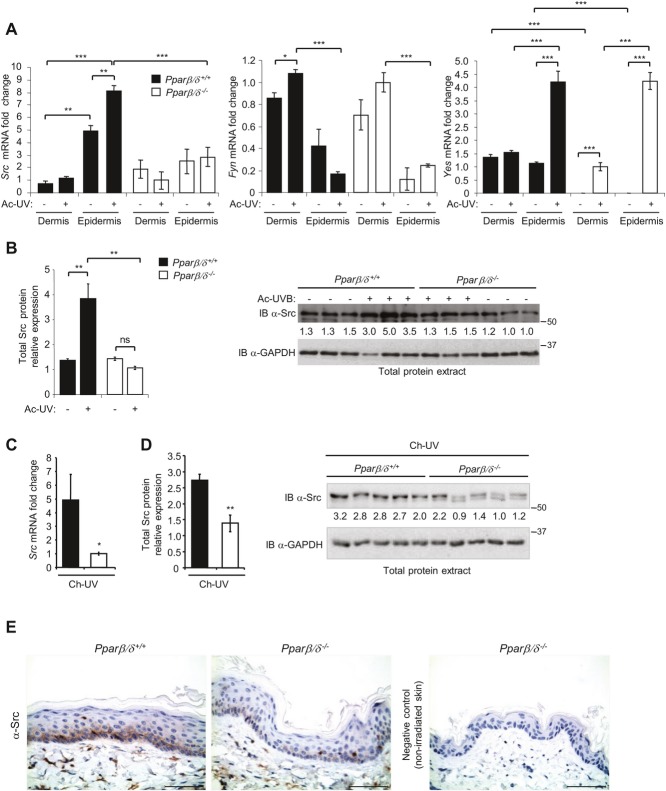
PPARβ/´ promotes Src expression in irradiated skin. A  *Src*, *Fyn* and *Yes* mRNA expression by RT-PCR in acutely (Ac-UV, +) and non-irradiated (−) dermis and epidermis of *Ppar*β/δ^*+/*+^ and *Ppar*β/δ^*−/−*^ mice. The value from irradiated dermis of *Ppar*β/δ^*−/−*^ mice was set to 1. Means ± SEM are presented (*n* = 6 mice/genotype/group). Data are representative of three independent experiments; *p*-values are, from top to bottom and from left to right, ***p = 0.0001, ***p = 0.0005, **p = 0.01, **p = 0.008 for *Src*; ***p = 0.0008, *p = 0.017, ***p = 0.0009 for *Fyn*; ***p = 0.0004, ***p = 0.0006, ***p = 0.0005, ***p = 0.0004, ***p = 0.0008, ***p = 0.0001, ***p = 0.001 for *Yes* calculated by two-tailed Student's *t*-test. B  Right, immunoblot of total Src in Ac-UV and non-irradiated skin of *Ppar*β/δ^*+/*+^ and *Ppar*β/δ^*−/−*^ mice. One of three independent experiments is shown (*n* = 6 mice/genotype/group). GAPDH was used as loading control; IB, immunoblot; left, quantification of the immunoblot (right) of total Src in Ac-UV and non-irradiated skin of *Ppar*β/δ^*+/*+^ and *Ppar*β/δ^*−/−*^ mice. Values are normalized to GAPDH, *n* = 3 mice/group; from left to right, **p = 0.010, 0.009, *t*-test. C  *Src* mRNA levels in non-tumoural skin of chronically irradiated (Ch-UV)*Ppar*β/δ^*+/*+^ mice and *Ppar*β/δ^*−/−*^ mice. Value from irradiated skin of *Ppar*β/δ^*−/−*^ was set to 1. Means ± SEM are given for 13 *Ppar*β/δ^*+/*+^ and 9 *Ppar*β/δ^*−/−*^ mice; *p = 0.035, *t*-test. D  Left, quantification of Src protein from the immunoblot (right) normalized to GAPDH (*n* = 5); **p = 0.009, *t*-test. Right, immunoblot of Src levels in Ch-UV non-tumourigenic skin of *Ppar*β/δ^*+/*+^ and *Ppar*β/δ^*−/−*^ mice (*n* = 5). One of three independent experiments is shown. E  Representative pictures of Src immunohistochemistry in Ch-UV skin of 13*Ppar*β/δ^+/+^ and 9 *Ppar*β/δ^*−/−*^ mice. Scale bars, 50 μm.

Our previous gene expression analyses indicated a higher expression of the tyrosine kinase *Src*, a well-characterized proto-oncogene and enhancer of skin carcinoma (Guasch *et al*, 2007; Joseloff *et al*, 2002; Matsumoto *et al*, 2003; Matsumoto *et al*, 2004; Serrels *et al*, 2009; Yagi *et al*, 2007), in wild-type versus *Ppar*β*/*δ^−/−^ primary keratinocytes (supplementary Fig S3A) (Montagner *et al*, 2011). In addition to Src, the Fyn and Yes tyrosine kinases, which also belong to the Src family kinases, were expressed in skin (Fig 2A) (supplementary Fig S3B). Interestingly, acute UV irradiation caused a selective PPARβ/δ-dependent enhancement of Src mRNA and protein expression *in vivo* (Fig 2A, B) (supplementary Fig S3B). Similarly, Src mRNA and protein levels were consistently and strikingly higher *in vivo* in chronically irradiated wild-type skin versus *Ppar*β*/*δ^−/−^ skin (Fig 2C, D). This effect was selective since the expression of *Fyn* and *Yes* was not affected after chronic irradiation (supplementary Fig S3C).

**Figure 3:**
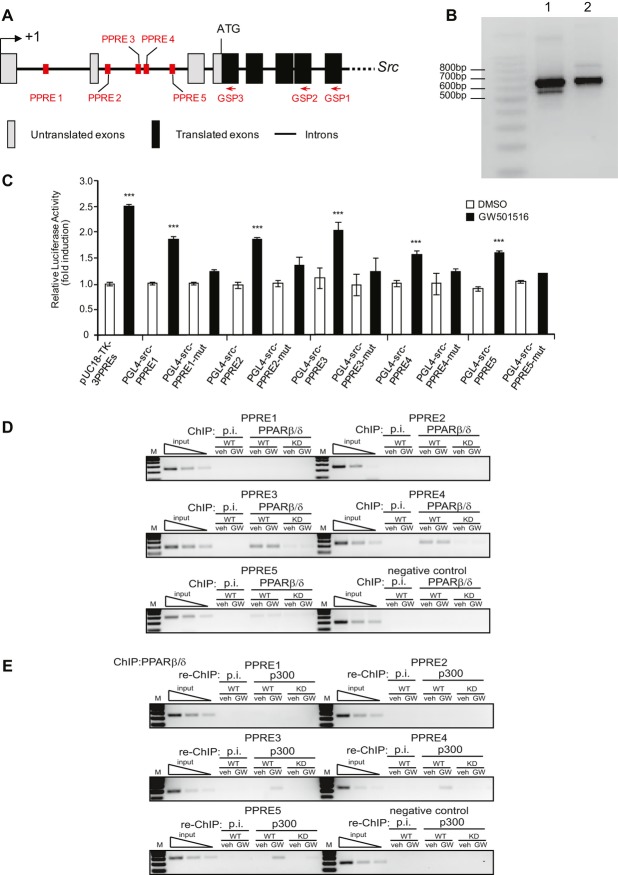
Src is a direct PPARβ/´ target gene. A  Schematic structure of the murine *Src* gene. Untranslated and translated exons of Src-001 transcript and position of primers used for 5′ RACE are depicted as indicated. GSP, gene-specific primer. B  Identification of the Src transcript isoform expressed in wild-type primary keratinocyte cultures treated with GW501516 (100 nM) for 24 h (lane 1) and total skin of SKH-1 wild-type mice after acute irradiation (lane 2). In both samples, a major 650-bp product was obtained. Sequencing of the isolated DNA bands indicated that the transcripts corresponded to the Src-001 transcript (ENSMUST00000029175). C  PPARβ/δ activity on wild-type or mutated PPREs in the Src promoter (supplementary Fig S5) via luciferase assays in NIH3T3 cells treated with GW501516 (300 nM) or vehicle (DMSO). Fold inductions were calculated as the ratio of firefly luciferase to Renilla luciferase activity using DMSO-treated NIH3T3 cells transfected with a TK-3PPRE construct designated as 1. Values are shown as the mean ± SD (*n* = 3).*p*-values are, from left to right, ***p = 0.0002, 0.0003, 0.0001, 0.0032, 0.0001, 0.0008 calculated by two-tailed Student's *t*-test. One of three independent experiments is shown. D,E  Representative results of ChIP experiments using anti-PPARβ/δ antibody (D) followed by re-ChIP with anti-p300 (E) performed in mouse keratinocytes (MKs) downregulated for PPARβ/δ expression by siRNA [knockdown cells (KD)] or not (wild-type cells, WT) treated or not with GW501516. The results show a PCR amplification of the PPRE3, 4 and 5 sites. Preimmune serum (p.i.) served as a control for ChIP. Primer sequences are given in supplementary Table S3. Data are representative of *n* = 3 independent experiments.

The highest PPARβ/δ-dependent Src expression occurred in the epidermis, mainly in keratinocytes of the lower layers (Fig 2A, E) (supplementary Fig S4) where PPARβ/δ has been localized (Michalik *et al*, 2001). Consistent with these observations, human immortalized keratinocytes (HaCaT cells) stimulated with the PPARβ/δ agonist GW501516 exhibited higher Src mRNA and protein expression, similar to ANGPTL4, which was used as a positive control of PPARβ/δ activity (supplementary Fig S5A, B) (Kersten *et al*, 2000; Rieck *et al*, 2008). Characterization by 5′ RACE and sequencing of the *Src* transcript induced in a PPARβ/δ-dependent manner by GW501516 in primary keratinocyte culture or by UV in mouse skin indicated its correspondence with the Src-001 transcript described in Ensembl (ENSMUST00000029175) (Fig 3A, B). *In silico Src* analysis revealed five peroxisome proliferator response elements (PPREs), which resemble the consensus PPRE motif, in the intragenic *Src* regulatory region (Fig 3A) (supplementary Fig S5C). These elements were potentially responsive to PPARβ/δ activation in a reporter assay (Fig 3C), indicating that *Src* is likely to be a PPARβ/δ target gene. This finding is in agreement with PPARβ/δ-induced *SRC* expression in the absence of *de novo* protein synthesis (supplementary Fig S5D).

To determine whether these PPREs serve as binding sites for PPARβ/δ, chromatin immunoprecipitation (ChIP) PPARβ/δ experiments followed by re-ChIP p300, a PPARβ/δ co-activator, were performed using lysates from control (wild-type) and PPARβ/δ-knockdown (KD) mouse keratinocytes (MKs, Fig 3D, E). The endogenous PPARβ/δ of KD cells was strongly downregulated by siRNA (supplementary Fig S5E). PPARβ/δ was constitutively associated with PPRE 3, 4 and 5 among the five PPREs identified *in silico* and tested in transactivation assays (Fig 3A, C), but p300 was recruited by PPARβ/δ only upon GW501516 activation. Thus, ligand activation appeared to be necessary for PPARβ/δ to regulate *Src* gene expression (Fig 3E), as for *Tgf*β*1* (supplementary Fig S6).

Collectively, these observations demonstrated that UV induced PPARβ/δ expression and activity. This activity in turn directly increased Src gene and protein expression, results that define *Src* as a novel PPARβ/δ target gene.

### PPARβ/δ enhances the Src-dependent EGFR/MAPK Erk1/2 signalling pathway in response to UV

**Figure 4:**
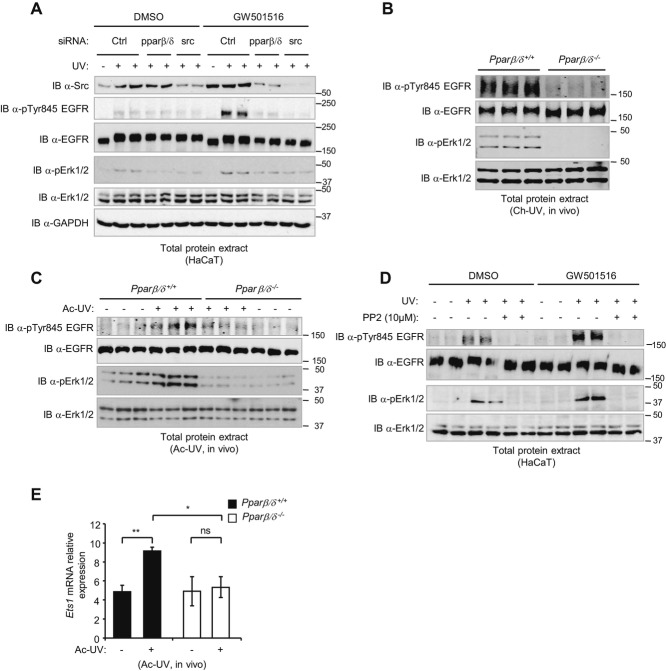
PPARβ/´-dependent upregulation of Src expression enhances EGFR/Erk1/2 signalling upon UV exposure in vitro and in vivo. A  Immunoblots of Src, pTyr845 EGFR, total EGFR, pErk1/2 and total Erk1/2 levels from whole-cell lysates of HaCaT cells transiently transfected with control (Ctrl), *Ppar*β/δ or *Src* siRNA, treated 24 h with GW501516 or DMSO, and then subjected to UVB radiation (40 mJ/cm^2^) before harvesting 30 min later. GAPDH was used as a loading control. Data are representative of three independent experiments. B,C  Immunoblot of pTyr845 EGFR, total EGFR, pErk1/2 and total Erk1/2 levels in protein extracts from chronically irradiated (Ch-UV) (B) or acutely irradiated (Ac-UV) (C) dorsal skin of *Ppar*β/δ^*+/*+^ and *Ppar*β/δ^*−/−*^ mice. Data are representative of three independent experiments. D  Immunoblot of pTyr845 EGFR, total EGFR, pErk1/2 and total Erk1/2 levels from whole-cell lysates of HaCaT cells treated with the PPARβ/δ agonist GW501516 or DMSO for 24 h before UVB exposure (40 mJ/cm^2^) in the presence or absence of a Src family kinase inhibitor (PP2), and harvested 30 min later. Data are representative of three independent experiments. E  Real-time RT-PCR of *Ets1* mRNA expression in non-irradiated and Ac-UV dorsal skin of *Ppar*β/δ^*+/*+^ and *Ppar*β/δ^*−/−*^ mice. Data are representative of three independent experiments for 12 *Ppar*β/δ^*+/*+^ and 12 *Ppar*β/δ^*−/−*^ mice; *p*-values are *p = 0.032, **p = 0.009 calculated by two-tailed Student's *t*-test.

UV irradiation activates the EGFR/MAPK Erk1/2 pathway via Src (Bode & Dong, 2003; Fritsche *et al*, 2007), a key mechanism in skin photocarcinogenesis in mice and humans (Rho *et al*, 2011). The possibility that PPARβ/δ activates not only Src but also the entire Src/EGFR/Erk1/2 downstream pathway was tested next in various PPARβ/δ and Src gain- and loss-of-function models. Src and Erk1/2 phosphorylation upon UV treatment was higher in wild-type than in PPARβ/δ^−/−^ primary keratinocytes (supplementary Fig S7), confirming the importance of PPARβ/δ activity for UV-dependent full activation of Src/Erk1/2. KD of PPARβ/δ in HaCaT cells consistently abolished UV-induced activation of the EGFR/Erk1/2 pathway (Fig 4A) (supplementary Fig S8), as did the absence of PPARβ/δ in skin *in vivo* (Fig 4B, C). Conversely, activation of PPARβ/δ by its agonist GW501516 enhanced the UV-induced phosphorylation of EGFR (Tyr845) and Erk1/2 in HaCaT cells (Fig 4A, D). Importantly, this action of PPARβ/δ completely depended on Src activity because Src loss of function, either by pharmacological inhibition using a PP2-specific Src family kinase inhibitor or Src KD by siRNA (supplementary Fig S8), prevented an increase in EGFR and Erk1/2 phosphorylation (Fig 4A, D). Taken together, these observations provide evidence in support of a PPARβ/δ-dependent UV stimulation of the entire Src/EGFR/Erk1/2 signalling pathway. Finally, *Ets1*, whose expression is stimulated by activated Erk1/2 (Fitsialos *et al*, 2007), was enhanced in the UV-irradiated dorsal skin of wild-type mice only (Fig 4E).

**Figure 5:**
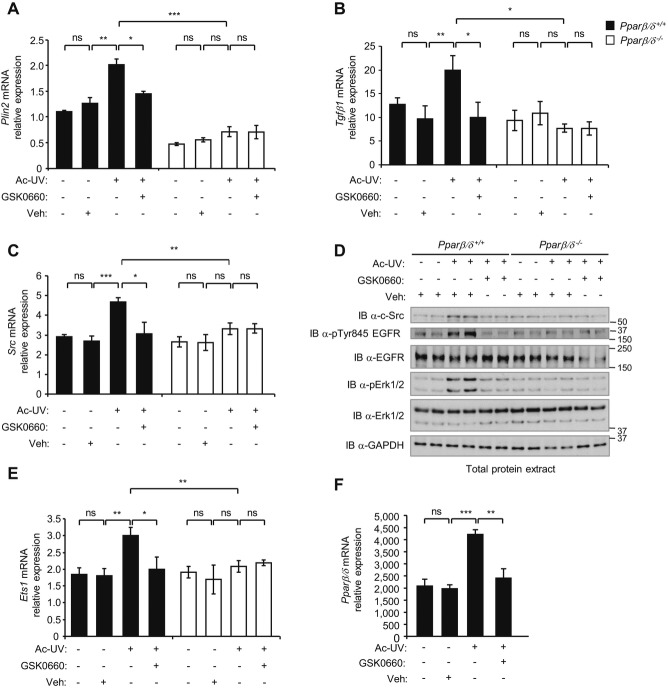
Pharmacological inhibition of PPARβ/´ prevents UV-induced Src expression and Src-dependent activation of EGFR/Erk1/2 signalling in vivo. Dorsal skin of *Ppar*β/δ^*+/*+^ and *Ppar*β/δ^*−/−*^ mice was topically treated with the PPARβ/δ antagonist GSK0660 or vehicle (Veh; 70% ethanol) prior to acute UV irradiation (Ac-UV); mice were sacrificed 24 h later. A–C  Quantification of *Plin2* (A), *Tgf*β*1* (B) and *Src* (C) mRNA expression via real-time RT-PCR. Means ± SEM are presented (*n* = 6 mice/genotype/group). Data are representative of two independent experiments; *p*-values are *p = 0.0190, **p = 0.0066, ***p = 0.0004 for *Plin2*; *p = 0.0421, 0.0153 (from left to right) and **p = 0.0098 for *Tgf*β*1*; *p = 0.0428, **p = 0.0098, ***p = 0.0045 for *Src* calculated by two-tailed Student's *t*-test; ns, not significant. D  Immunoblot of total Src, EGFR, p-Tyr845 EGFR, Erk1/2 and p-Erk1/2 protein in extracts from dorsal skin of *Ppar*β/δ^*+/*+^ and *Ppar*β/δ^*−/−*^ mice. Data are representative of two independent experiments (*n* = 12 mice/genotype/experiment). E  Quantification of *Ets1* mRNA expression by real-time RT-PCR. Means ± SEM are given (*n* = 6); *p*-values are *p = 0.0324 and **p = 0.0091, 0.082 (from left to right) calculated by two-tailed Student's *t*-test. F  Quantification of *Ppar*β*/*δ expression via real-time RT-PCR. Means ± SEM are presented (*n* = 6 mice/genotype/group). Data are representative of two independent experiments; *p*-values are **p = 0.0083 and ***p = 0.0006 calculated by two-tailed Student's *t*-test; ns, not significant.

Wild-type and *Ppar*β/δ^−/−^ mice were then topically treated with the PPARβ/δ antagonist GSK0660 prior to acute UV irradiation. The UV-induced expression of two PPARβ/δ target genes, *Plin2* and *Tgf*β*1*, was prevented by GSK0660 (Fig 5A, B), which also abolished the increase in Src gene and protein expression (Fig 5C, D), resulting in both decreased phosphorylation levels of EGFR and Erk1/2 (Fig 5D) and stimulation of *Ets1* (Fig 5E). Interestingly, the drug did not affect expression of the pro-inflammatory markers *Ptgs2* (*Cox2*) and *Il6* (supplementary Fig S9A, B) or of *Ppar*γ and *Ppar*α (supplementary Fig S9C, D). However, UV-induced *Ppar*β*/*δ upregulation was abolished, suggesting a positive feedback loop in PPARβ/δ expression, possibly involving Src (Fig 5F). Taken together, these observations identified PPARβ/δ as a positive regulator of a major pathway in skin carcinogenesis via its effect on Src expression and activity.

### PPARβ/δ coordinates a pro-tumoural gene program in advanced actinic keratosis

**Figure 6:**
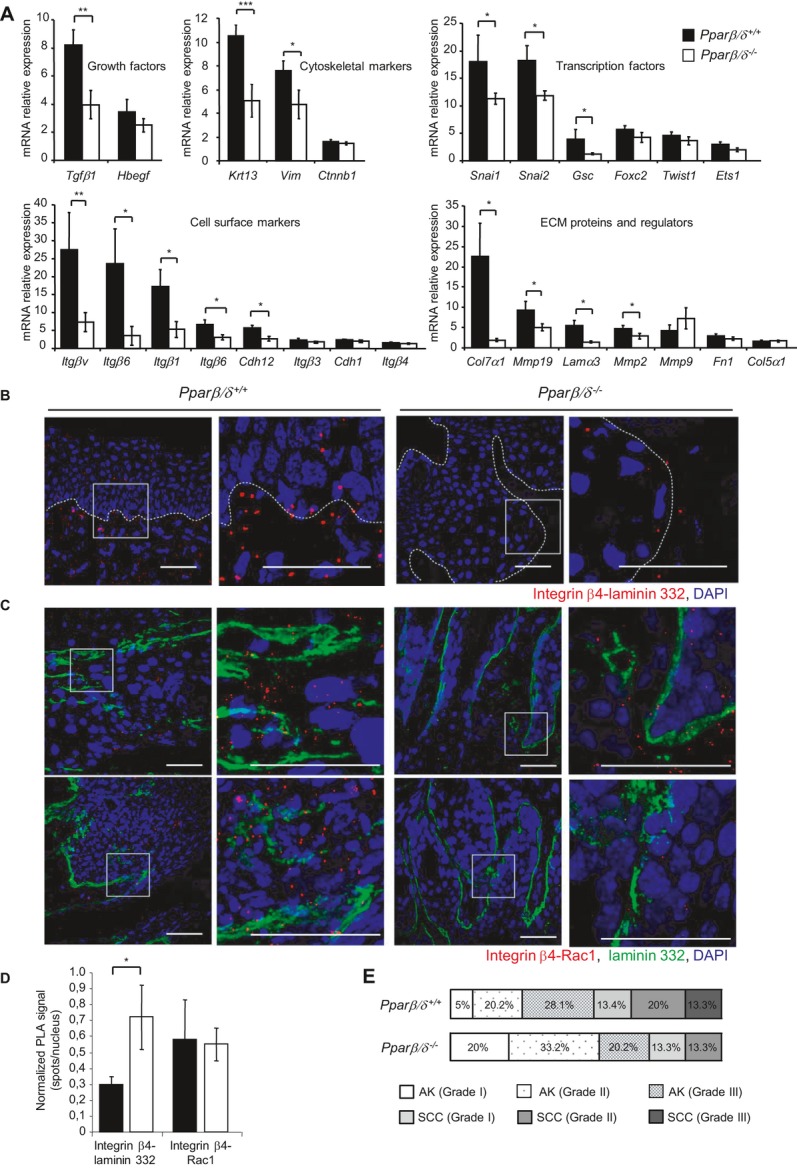
PPARβ/´ upregulates pro-tumoural marker gene expression and laminin 332 deposition in actinic keratosis, enhancing SCC progression. A  mRNA expression of epithelial-to-mesenchymal transition (EMT) markers in 20 actinic keratoses with moderate atypia (grade II) of *Ppar*β/δ^*+/*+^ and *Ppar*β/δ^*−/−*^ mice by real-time RT-PCR. Means ± SEM are presented; *p*-values are **p = 0.0077 (*Tgf*β*1*), ***p = 0.0009 (*Krt13*), *p = 0.046 (*Vim*), *p = 0.045 (*Snai1*), *p = 0.032 (*Snai2*), *p = 0.028 (*Gsc*), **p = 0.009 (*Itg*α*v*), *p = 0.025 (*Itg*α*6*), *p = 0.022 (Itgβ1), *p = 0.013 (Itgβ6), *p = 0.035 (Cadh12), *p = 0.020 (Col7α1), *p = 0.035 (Mmp19), *p = 0.025 (Lamα3), *p = 0.043 (Mmp2) calculated by two-tailed Student's *t*-test. The full gene names are given in supplementary Table S2. B  Representative immunofluorescence staining of PLA experiments for laminin 332/β4 integrin (red spots) in actinic keratosis with moderate atypia (grade II) sections from *Ppar*β/δ^*+/*+^ and *Ppar*β/δ^*−/−*^ mice. DAPI, blue. Scale bars, 50 μm. C  Representative immunofluorescence staining of PLA experiments for β4 integrin/Rac1 (red spots) and laminin 332 in actinic keratosis with moderate atypia (grade II) sections from *Ppar*β/δ^*+/*+^ and *Ppar*β/δ^*−/−*^ mice. DAPI, blue. Scale bars, 50 μm. D  Quantification of PLA staining. Average number of PLA signals (red spots) per nucleus from *n* = 10 actinic keratoses with moderate atypia sections/genotype (*p = 0.024, *t*-test). E  Actinic keratosis (AK) with grading of cellular atypia (mild, grade I; moderate, grade II; severe, grade III) and SCC distribution in 25 tumours collected from each *Ppar*β/δ^*+/+*^ and *Ppar*β/δ^*−/−*^ mouse graded histologically in a blinded manner according to Rowert-Huber et al and to the Broders' classification, respectively.

Skin tumour growth is associated with increased proliferation and migratory potential of keratinocytes, and in this process, Src promotes epithelial cell dedifferentiation during the epithelial-to-mesenchymal transition (EMT). The EMT might also be regulated by other direct or indirect PPARβ/δ-modulated signalling pathways such as the Tgfβ1/SMAD pathway, which could act on *Src* expression (Glick, 2012; Han *et al*, 2005; Hoot *et al*, 2008; Martins *et al*, 2009; Nakamura & Tokura, 2011; Newkirk *et al*, 2007). Thus, we examined the ability of PPARβ/δ to enhance this transition by measuring expression of EMT markers in actinic keratosis with moderate atypia (grade II) of wild-type and *Ppar*β*/*δ^−/−^mice, including growth factors, transcription factors, cytoskeletal and cell surface markers, extracellular matrix proteins and regulators (Fig 6A). The expression of many of these markers was higher in wild-type mice than in *Ppar*β*/*δ^−/−^ mice, suggesting that the wild-type advanced grade actinic keratosis presented a higher risk for premalignant progression. Results of basement membrane analysis using laminin 332 (α3β3γ2) immunofluorescence supported this hypothesis (supplementary Fig S10A). The basement membrane between the basal keratinocyte layer and the adjacent stroma exhibited a clear lining of laminin 332 in *Ppar*β*/*δ^−/−^ actinic keratosis with moderate atypia (grade II) whereas laminin 332 deposition was more diffuse and thicker in lesions of wild-type mice, reflecting perturbations of the basal layer. The proximity ligation assay (PLA) showed that these laminin 332 infiltrates in the basal and suprabasal epidermal layers in wild-type mice could bind β4 integrin (Fig 6B, red positive signal quantified in Fig 6D) and transduce signal via Rac1 (Fig 6C, red positive signal quantified in Fig 6D). These observations were consistent with the increased expression compared to *Ppar*β*/*δ^−/−^ mice of EMT markers in wild-type grade II actinic keratoses, which displayed a higher level of proliferating keratinocytes and more intense Keratin 13 staining, an early marker of malignant progression susceptibility (supplementary Fig S10B and S11). Moreover, histological grading of SCCs from wild-type and *Ppar*β*/*δ^−/−^mice according to the Broders' classification (Broders, 1921) showed that wild-type mice developed more advanced SCCs (Fig 6E) (supplementary Fig S12). However, because of the conditions imposed by the Veterinary Office (see Material and Methods for details), it was not possible to obtain more advanced UV-induced skin tumours (Grade IV according to Broders’ classification) to observe metastatic SCCs. Interestingly, EMT markers were also expressed at higher levels in grade II SCCs from wild-type compared to *Ppar*β*/*δ^−/−^ mice at the mRNA and protein levels. N-Cadherin staining confirmed this result (supplementary Fig S13).

Collectively, these observations suggested that keratinocytes of wild-type mice were more likely to invade the surrounding stroma. This characteristic represents a higher risk of malignant conversion in these mice compared to the *Ppar*β*/*δ^−/−^ mice.

### Correlation between PPARβ/δ and Src expression in various human carcinomas

**Figure 7:**
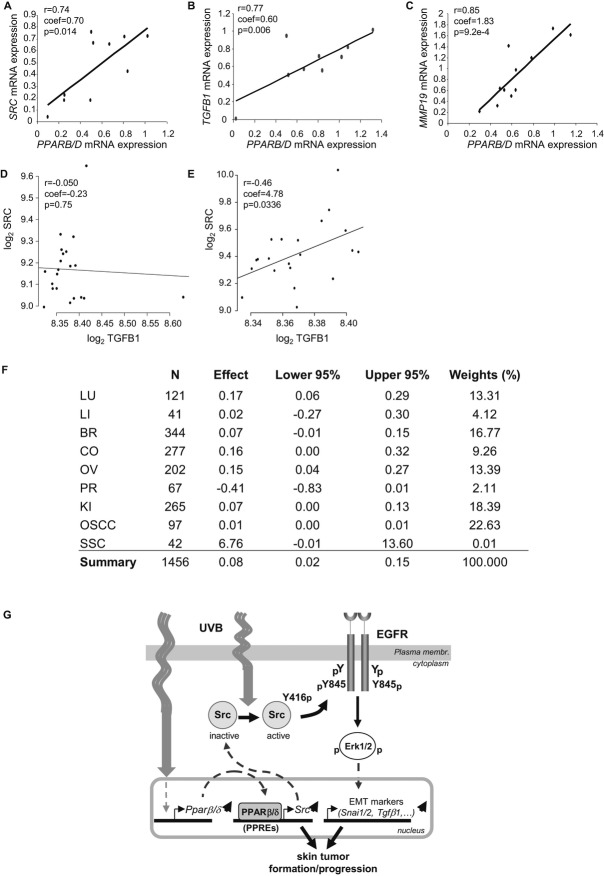
PPARB/D and SRC mRNA expression are correlated in human skin SCC and in other types of human carcinomas. A–C  Correlation between *PPARB/D* and *SRC* (A), *PPARB/D* and *TGFB1* (B) and *PPARB/D* and *MMP19* (C) expression as assessed via real-time RT-PCR of RNA extracted from human SCC biopsies (*n* = 9). *p*-values are calculated by two-tailed Student's *t*-test. D,E  Interaction between *TGFB1* and *PPARB/D* underlies *SRC* expression levels. All 42 SCCs were sorted by *PPARB/D* expression. Samples with either the highest (*n* = 21; top 50% of the samples) or lowest (*n* = 21, bottom 50% of the samples) *PPARB/D* levels were identified. (D) Least squares regression between *TGFB1* and *SRC* expression levels in samples with the lowest *PPARB/D* expression. The black line represents the least square fit. The observed linear regression coefficient (−0.23) was not significantly different from zero (Student *p*-value = 0.75). (E) Linear regression between *TGFB1* and *SRC* levels in the 21 tumour samples with the highest *PPARB/D* expression revealed a significant linear relationship. F  Table presenting the interaction coefficient β3 (estimate) and its 95% confidence interval for the linear model SRC ∼ β0 + β1TGFB1SRC + β2PPARB/D + β3TGFB1SRC: PPARB/D. The summarized meta-analysis of the interaction coefficient was estimated using a random effects model. G  Model of the molecular function of PPARβ/δ in UV-induced skin tumours. UV irradiation induces PPARβ/δ gene expression and activation. Once activated, PPARβ/δ drives the expression of *Src*, which correlates with an increased Src protein level and higher kinase activity. This activity leads to the activation of the Src-dependent EGFR/Erk1/2 signalling pathway, which drives the expression of genes involved in the epithelial-to-mesenchymal transition (EMT). In coordination with other PPARβ/δ-dependent or -independent mechanisms and/or genetic defects, this mechanism enhances skin tumour formation and progression upon UV exposure, identifying PPARβ/δ as a putative inducer of carcinoma.

We wished to investigate whether the proto-oncogene *Src* is also a PPARβ/δ target in human SCC. Interestingly, the levels of *SRC* and *PPARB/D* expression were highly and significantly correlated in these tumours (Fig 7A), as were the expression levels of *PPARB/D* and*TGFB1* and *MMP19*, respectively, and to a lesser extent, *MMP2*, *VEGFA*, *VIM* and *SNAI1* (Fig 7B, C) (supplementary Fig S14A–D). These data suggested that, as in UV-irradiated mouse skin, PPARβ/δ regulates *SRC* expression in human SCC, which is correlated with the expression of EMT markers.

To further examine the underpinnings of these observations, we investigated the relationships among *SRC*, *TGFB1* and *PPARB/D* expression levels in human carcinomas from various organs. More specifically, we tested whether the degree of linear dependency between SRC and TGFB1 mRNA levels (degree of correlation) is influenced by the abundance of PPARB/D mRNA present in the tumour (the significance of the interaction term TGFB1: PPARB/D of the linear model SRC ∼ TGFB1 + PPARB/D + TGFB1: PPARB/D). This linear model was separately fitted to nine human tumour types, including skin SCC (Fig 7–D–F) (supplementary Fig S14E–I). The least square estimation of the model parameter revealed that the TGFB1: PPARB/D interaction coefficient term was significantly different from zero for lung (0.17 [0.06–0.29], 95% confidence interval in square brackets) and ovarian (0.15 [0.04–0.27]) carcinomas and oral squamous cell carcinoma (OSCC) (0.009 [0.003–0.014]). Combining these nine independent interaction coefficients into a random effects model, a method commonly used in statistical meta-analysis, produced a significant summarized interaction coefficient (0.081 [0.014–0.146]; Fig 7F). Results for SCC show that patients with a*PPARB/D* mRNA abundance lower than the median value had an absence of linear dependency between *SRC* and *TGFB1* (*p* = 0.75; Fig 7D) but that those with a *PPARB/D* abundance above or equal to the median of the dataset showed a significant linear relationship (*p* = 0.0336; Fig 7E). Results with the ovarian carcinoma and OSCC datasets led to similar inferences (supplementary Fig S14F–I).

Thus, our statistical modelling provided evidence that the expression level of *PPARB/D* influences the degree of correlation between *SRC* and*TGFB1* levels across a wide variety of human carcinomas, an observation that supports our hypothesis of a direct influence of PPARβ/δ on *SRC* and *TGFB1* transcription. Taken together, our observations and analyses indicate that PPARβ/δ regulates *SRC* in human SCC, notably in skin SCC, and that this regulation also occurs in carcinomas of various tissue origins, thus revealing a broad pro-tumourigenic potential of PPARβ/δ via *SRC* upregulation.

## Discussion

Our study demonstrates that PPARβ/δ promotes skin cancer by enhancing Src activity in response to UV radiation. UV is a skin carcinogen causing both DNA damage resulting in tumour initiation and activation of signalling cascades that promote tumour development (Devary *et al*, 1993; Matsumura & Ananthaswamy, 2004; Rho *et al*, 2011). The signalling pathway involving the tyrosine kinase Src, the tyrosine kinase receptor EGFR and the MAPK Erk1/2 cascade is activated during UV-induced skin inflammation and carcinogenesis, promoting initiation, progression and malignant conversion. This pathway is therefore a target for epithelial cancer prevention (Fritsche *et al*, 2007; Rho *et al*, 2011). Importantly, this pathway and its cellular effects may also be modulated by other PPARβ/δ-dependent or -independent signalling pathways, such as Tgfβ1/SMAD.

To date, the nuclear receptor PPARβ/δ has been less studied than PPARα and PPARγ, which are drug targets for the treatment of dyslipidemia and type 2 diabetes, respectively. Recently, the US Food and Drug Administration released safety information linking pioglitazone, which targets PPARγ, to increased risk for bladder cancer. Although there is growing interest in developing PPARβ/δ agonists to treat dyslipidemia, no PPARβ/δ compound has been brought to market, and the role of this isotype in tumourigenesis has been debated (Michalik *et al*, 2004; Peters & Gonzalez, 2009; Peters *et al*, 2012).

Our study has yielded the first insights into how PPARβ/δ promotes UV-induced skin tumourigenesis (Fig 7G). *In vivo* UV exposure increases PPARβ/δ expression at the transcriptional level and its activity. Once activated, PPARβ/δ upregulates Src expression and activity, which in turn, boosts EGFR/Erk1/2 signalling. Thus, in our mouse model, PPARβ/δ sensitizes keratinocytes to UV, promotes actinic keratosis development and progression, and stimulates the expression of EMT markers that affect basement membrane integrity. Importantly, *PPARB/D*expression and *SRC*, *MMP19* and *SNAI1* expression directly correlate in human skin SCC, which may indicate a direct effect of PPARβ/δ on EMT initiation. Furthermore, our meta-analysis of PPARβ/δ activity in various epithelial tumours, including human skin SCC, revealed a positive interaction between *PPARB/D* expression levels and those of two of its targets, *SRC* and *TGFB1*. Interestingly, the identification of this relationship in large independent datasets of various tumour types, particularly in lung, colon and ovarian cancers known for their high levels of Src expression and activity, strongly supports a regulatory role for PPARβ/δ in human carcinomas. We speculate that early in skin actinic keratosis formation, PPARβ/δ regulates Src and may act, in concert with other genetic change, to promote tumour progression by increasing keratinocyte migratory and proliferative properties. Our data help to define the pro-carcinogenic properties of PPARβ/δ in skin carcinoma and most likely in carcinomas from other tissue origins, as well, and identify *Src* as a direct PPARβ/δ target gene, at least in mouse.

In accordance with our observations, ErbB2 activation, in concert with ErbB3 and ErbB4, leads to the expression of *FABP5*, an intracellular lipid binding protein, which plays a crucial role in PPARβ/δ transcriptional activation in the MCF7 cell line, suggesting that the EGFR superfamily could also act upstream of PPARβ/δ to regulate its activity in a model of breast cancer (Kannan-Thulasiraman *et al*, 2010). Although its consequences remain unknown, the expression of PPARβ/δ has been reported to be increased in skin pathologies such as the lesional skin of psoriatic patients (Romanowska *et al*, 2010; Westergaard *et al*, 2003) and in human skin biopsies of premalignant and malignant skin carcinoma (Nijsten *et al*, 2005; Sertznig *et al*, 2008). In the latter, increased PPARβ/δ expression has been associated with a significant increase in microvessel density and strongly correlates with the expression of COX2, an enzyme implicated in skin carcinoma development (Trifan & Hla, 2003). Interestingly, our previous findings of PPARβ/δ participation in keratinocyte survival, proliferation and migration during skin wound healing (Tan *et al*, 2007) are somewhat analogous to the tumour-development functions described in the current investigation, providing a further example of the molecular parallels between wound repair and cancer. It is intriguing to note that we and others have also described PPARβ/δ as a pro-differentiating factor in keratinocytes (Burdick *et al*, 2007; Peters & Gonzalez, 2009; Tan *et al*, 2001). Similarly, a pro-differentiation function has been attributed to the MAPK-Erk1/2 pathway in keratinocytes, a pathway that is clearly pro-carcinogenic in skin tumours (Rho *et al*, 2011; Seo *et al*, 2004). These apparently contradictory pro-differentiation and pro-carcinogenic roles may be due to different environmental stimuli and/or genetic contexts, which could lead to different kinetics and amplitude of Erk1/2 stimulation, switching the spectrum of their target proteins. This model could also accommodate a role for PPARβ/δ. Indeed, increasing evidence suggests that physiological or pathological contexts, including the genetic background, drive PPARβ/δ functions and might influence the extracellular signals, endogenous ligands produced, ligand-binding protein expression and post-translational modifications. These parameters are likely to affect the level and activity of PPARβ/δ and the expression pattern of its numerous target genes.

This hypothesized complexity could explain the between-study discrepancies regarding the role of PPARβ/δ in skin carcinogenesis. For example, Kim *et al* explored the anti-cancer properties of PPARβ/δ in skin using DMBA/TPA-induced carcinogenesis, a context in which tumour development was more severe in *Ppar*β*/*δ^−/−^ mice than in wild-type animals, suggesting that PPARβ/δ attenuates the development of chemically induced skin cancer (Kim *et al*, 2004). Inhibition of keratinocyte proliferation was thought to mediate this effect of PPARβ/δ mainly via the activity of protein kinase Cα. However, in transgenic mice that over-express protein kinase Cα, the susceptibility to DMBA/TPA-induced skin tumourigenesis was not affected (Cataisson *et al*, 2003; Wang & Smart, 1999). Moreover, induction of skin cancer by DMBA/TPA involves different molecular mechanisms from those associated with human skin cancer attributable to chronic UV exposure (Boukamp, 2005; Matsumura & Ananthaswamy, 2002). Notably, tumour initiation with a unique topical sub-carcinogenic dose of a genotoxic carcinogen (DMBA) primarily induces *Ha-Ras*-activating mutations, whose frequency reaches only 10–20% in human SCCs and basal cell carcinomas, reflecting a marginal contribution of this oncogene to these carcinomas (Campbell *et al*, 1993; Lieu *et al*, 1991; Pierceall *et al*, 1991). In this context, our proposed model pathway could be masked by such *Ha-Ras*-activating mutations, rendering the MAPK Erk1/2 activation upstream independent of Src/EGFR stimulation.

Moreover, this apparent controversial function of PPARβ/δ in cancer development is also illustrated in other cancer types for which several studies have suggested an attenuation of carcinogenesis by PPARβ/δ, while others support a tumour-promoting role (reviewed in Michalik *et al*, 2004; Peters & Gonzalez, 2009; Peters *et al*, 2012). For instance, studies indicate that colon carcinogenesis is exacerbated in the absence of PPARβ/δ expression and/or that ligand activation of PPARβ/δ attenuates tumourigenesis (Harman *et al*, 2004; Hollingshead *et al*, 2008; Reed *et al*, 2004). In contrast, other studies have shown that genetic disruption of PPARβ/δ inhibits colorectal tumourigenesis and that ligand activation of PPARβ/δ promotes tumourigenesis (Gupta *et al*, 2004; Park *et al*, 2001; Wang *et al*, 2006; Zuo *et al*, 2009). Many of these studies are based on PPARβ/δ mRNA or protein expression levels, which do not necessarily reflect the ligand-dependent activity of the receptor. Interestingly, the expression of both PPARβ/δ and COX-2 in tissues of colorectal cancer patients leads to liver metastases, a condition associated with poor prognosis (Yoshinaga *et al*, 2011). PPARβ/δ gene expression is upregulated in patients with non-small cell lung cancer and correlated with the expression of *VEGFA* and *COX2*. Moreover, cell lines with relatively high PPARβ/δ activity (*e.g*. H441 and H358) exhibit proliferative and pro-survival responses, which are absent or minimal in cells with low levels of the receptor (*e.g*. A549) (Genini *et al*, 2012). In addition, PPARβ/δ, via its regulation of ANGPTL4 gene expression, is suggested to be implicated in MDA-MB-231 cancer cell invasion into a three-dimensional matrix (Adhikary *et al*, 2012). These findings suggest a tumour-promoting function for PPARβ/δ, in accordance with our data on skin carcinoma, and this diversity of PPARβ/δ actions underscores the need for more work in mouse models and human tissue samples to decipher the complex PPARβ/δ functions in tumourigenesis. An illustration of this complexity is the importance of the isotype and expression levels of fatty acid-binding proteins in directing the same ligand towards RAR or PPARβ/δ. The latter promotes the pro-carcinogenic function of PPARβ/δ in breast tumour development (Kannan-Thulasiraman *et al*, 2010; Schug *et al*, 2007; Schug *et al*, 2008).

Src-centred investigations have thus far concentrated on the mechanisms of post-translational regulation of its kinase activity, but little is known regarding the factors regulating the expression of its gene. The observation that PPARβ/δ stimulates *Src* expression sheds new light on this powerful oncogene, and interest is growing in Src as a potential therapeutic target in skin carcinoma treatment (Serrels *et al*, 2009). Our study also raises fundamental biological questions related to cancer therapy. Inhibitors of Src are used in the treatment of many carcinomas but are associated with side effects (Kopetz *et al*, 2007). Because *Src* is a target of PPARβ/δ, specific antagonists of the receptor might be considered for the treatment of such cancers and/or prevention of malignant progression of actinic keratosis. Given that PPARβ/δ is a promising molecular target in the prevention and treatment of metabolic diseases such as dyslipidemia, obesity and diabetes, we suggest a careful evaluation of the use of high-affinity PPARβ/δ agonists such as GW501516, which is currently in phase IV clinical trials for the treatment of dyslipidemia (Ooi *et al*, 2011).

## Materials and Methods

### Animal model and UVB irradiation

SKH-1 hairless mice (Charles River) were crossed with SV129/BL6/J *Ppar*β*/*δ wild-type or *Ppar*β*/*δ^*−/−*^ mice to obtain SKH-1/*Ppar*β*/*δ wild-type and SKH1/*Ppar*β*/*δ^*−/−*^ mouse strains. Mice were housed in quarters with a 12/12-h light/dark cycle and maintained with water and food *ad libitum*. For acute and chronic UV exposure, mice were UV irradiated on their backs with a GL40E 40W tube (SNEE), which emits most of its energy within the UVB range (90%; emission spectrum 280–370; 10% UVA). Doses of UVB (312 nm) and UVA (370 nm) were monitored using an appropriate radiometer.

For acute exposure, female SKH-1 wild-type and *Ppar*β*/*δ^*−/−*^ mice ages 10–12 weeks were irradiated on their backs with the UV lamp. UVB radiation emission was controlled using a radiometer until a dose of 120 mJ/cm^2^ was delivered. Non-irradiated age-matched mice were used as controls. Twenty-four hours after UV irradiation, animals were sacrificed using CO_2_ gas, following by the acquisition of dorsal skin samples that were directly frozen in liquid nitrogen or prepared for histological analysis. For GSK0660 treatment, 200 μl of GSK0660 (625 μg/μl in 70% ethanol; Sigma, G5797) was applied topically on the back 1 h prior to UV exposure.

For chronic treatment, animals were irradiated on their backs three times per week with 70 mJ/cm^2^ of UVB, which was monitored using a radiometer. The time of tumour appearance, number and size were monitored twice per week. Mice with one tumour reaching 9 mm in diameter were sacrificed using CO_2_ gas in accordance with the requirements of the Veterinary Office of the Canton Vaud (Switzerland) and Federal Swiss Veterinary Office Guidelines. Non-irradiated aged-matched mice were simultaneously used as controls and handled in the same fashion as the irradiated animals. Dorsal tumours or non-tumoural irradiated skin samples were then obtained and prepared as described above.

### Tumour grading

Mouse skin tumours were paraformaldehyde-fixed and paraffin-embedded. Tissue sections (5 μm) were stained with hematoxylin/eosin. Histological analysis of actinic keratosis and tumour classification were performed in a blind manner by a pathologist. SCCs were classified according to the Broders' classification based on the degree of SCC keratinization and of keratinocyte differentiation (Broders, 1921). This classification is as follows: SCC Grade I: 75% keratinocytes are well differentiated; SCC Grade II: >50% keratinocytes are well differentiated; SCC Grade III: >25% keratinocytes are well differentiated and SCC Grade 4: <25% keratinocytes are well differentiated. Actinic keratoses were defined histologically and classified as grade I, II or III based on the degree of cytological atypia of epidermal keratinocytes and involvement of adnexal structures according to Rowert-Huber *et al* (Rowert-Huber *et al*, 2007). Representative pictures of actinic keratosis and SCCs are presented in supplementary Fig S12.

### Human skin biopsies

Cutaneous SCC samples were obtained anonymously from the Department of Dermatology, University Hospital of Lausanne, Switzerland. Normal skin was from healthy adult volunteers or from the edges of skin tumours. SCC was diagnosed by experienced pathologists. Informed consent for research was obtained prior to routine diagnostic services. All samples include the dermis and the epidermis.

### Preparation of expression data

We used expression microarrays from the Expression Project for Oncology Consortium (Affymetrix human genome HG-U133 plus 2.0 arrays) and from the Gene Expression Omnibus (accession code: GSE2109). Only the following tumour types were considered: breast (*n* = 344), lung (*n* = 121), liver (*n* = 41), pancreas (*n* = 14), colon (*n* = 277), ovarian (*n* = 202), prostate (*n* = 67) and kidney (*n* = 259). Other tumour types were excluded because of low sample numbers at the time of download.

Gene expression data were normalized with the robust multi-array method implemented in the library affymetrix. Three selection criteria were applied to probe sets: annotation to a defined Entrez gene, standard deviation of normalized expression greater than 0.1, and characterization as the most variable probe set for a given Entrez gene. The normalized, log-transformed expression measures were used for the analysis. For the OSCC dataset, because two datasets were available from the GEO database, optimal probe set selection among features mapped to TGFB1, SRC and PPARB/D was performed with the GSE30784 dataset and then applied in the GSE41613 (Chen *et al*, 2008; Lohavanichbutr *et al*, 2013). Only results obtained with the GSE41613 were considered in the main analysis. For the SSC dataset, the GSE32628 was taken using the normalized values provided by the contributor (Hameetman *et al*, 2013).

All data were analysed in the R programming environment (2.9.1). Linear regression models were used to assess the relationships between gene expression levels using additive and multiplicative models (interactions). Linear models were applied separately to each tumour type. A meta-analysis of the interaction coefficient estimates for the various tumour types was performed using a random effect model.

### Src cDNA isolation, polyA-tailing and 5′ RACE

To identify Src transcript isoform(s) induced by PPARβ/δ in skin and keratinocytes, Src mRNA was specifically reverse transcribed with Superscript II (Invitrogen) using 50 ng of primer GSP1 (Gene Specific Primer 1, primer sequence given in supplementary Table S2) from 5 μg of total RNA (extraction with TRIzol, Invitrogen) from a culture of wild-type primary keratinocytes treated for 24 h with GW501516 (100 nM) or total skin from wild-type SKH-1 mice acutely irradiated and sacrificed 24 h later. After treatment with RNase H (New England Biolabs) for 20 min at 37°C, first-strand Src cDNA was purified (Macherey-Nagel) before a polyA-tailing reaction in the presence of Tdt (Promega) and ATP. A first, PCR was performed using Frohmann and GSP2 (Gene Specific Primer 2) primers (Fig 3; sequences given in supplementary Table S2) using Herculase II Fusion DNA Polymerase (Agilent Technologies) according to the manufacturer's instructions. A nested PCR was then performed with Q0 and GSP3 (Gene Specific Primer 3) primers (Fig 3; sequences given in supplementary Table S2) using Herculase II Fusion DNA Polymerase (Agilent Technologies). PCR products were then purified (Macherey-Nagel) and loaded onto agarose gels for identification of the Src transcript(s). GSP1, GSP2 and GSP3 primers were designed to specifically amplify a region corresponding to Src-001, Src-007, Src-005, Src-006 or Src-201 transcripts described in Ensembl. The DNA bands revealed were then excised and purified before sequencing.

### PPRE subcloning, mutagenesis and promoter activity assay

To analyse Src promoter activity, various regions of the promoter (annotated from the transcriptional start site in the mouse genome) were inserted into the plasmid vector pGL4 (Promega, E8421) to generate pGL4-PPRE1, pGL4-PPRE2, pGL4-PPRE3, pGL4-PPRE4 and pGL4-PPRE5. pGL4-PPRE mutant plasmids carried mutated PPRE sequences introduced with the QuikChange Site-Directed Mutagenesis Kit (Stratagene). Primers used for mutagenesis are given in supplementary Table S1. NIH3T3 cells were plated the day before transfection in 12-well plates. Either 0.8 μg of wild-type or mutated pGL4-PPRE or 0.5 μg of 3 × -PPRE-TK-Luc (gift from R. Evans) plasmids were introduced into the cells via Superfect (Qiagen), together with 0.5 μg of pSG5-mouse-PPARβ/δ and 0.025 μg of a Renilla luciferase construct. After overnight serum starvation, transfected cells were treated for 24 h with GW501516 (100 nM) and then lysed in 1 × passive lysis buffer and analysed for dual luciferase activity following the manufacturer's instructions (Promega). Relative luciferase activity was calculated as the ratio of firefly to Renilla luciferase activity. Data represent means ± standard deviations of relative luciferase activities for DMSO performed in triplicate.

### Immunoblotting

Cells and tissue samples were lysed in TNE buffer containing 20 mM Tris [pH 7.4], 150 mM NaCl, 1 mM EDTA, 10% glycerol, 1% NP-40, 10 μg/ml leupeptin, 10 μg/ml aprotinin and 1 mM Na_3_VO_4_. After quantification, proteins were separated by SDS-PAGE and subjected to immunoblotting. All primary antibodies were incubated overnight in 1 × Tris-buffered saline plus 0.1% Tween-20 and 5% bovine serum albumin at a dilution of 1:1000 except where indicated otherwise. Cell Signalling was the source for the following antibodies: Src (2123), phospho-Tyr845 EGFR (6963), EGFR (4267), phospho-Erk1/2 (4370), Erk1/2 (9102), β-Catenin (8480), phospho-Ser675 β-Catenin (4176), Slug (9585) and GAPDH (2118). Anti-β-tubulin and N-Cadherin were purchased from BD Biosciences (556321) and Millipore (04-1126; 1/50 000), respectively.

### ChIP and re-ChIP

ChIP experiments were carried out with modifications of the experimental setup described by Tan *et al* (Tan *et al*, 2004). Briefly, chromatin was crosslinked using 0.5% formaldehyde for 10 min at 37°C and sonicated in SDS lysis buffer (1% SDS, 10 mM EDTA and 50 mM Tris-HCl, pH 8.1) to obtain crosslinked DNA that measured 200–500 bp in length. Approximately 10% of the supernatant was retained as input, while the remaining amount was processed with the ChIP using anti-PPARβ/δ antibody (4 μg; SC-7197, TransCruz Grade; Santa Cruz), and complexes were pulled-down by Protein A/G (Santa Cruz, CA, USA). Re-ChIP was performed by subsequent probing with anti-p300 antibody (4 μg; 05–257, Millipore); DNA fragments were reverse crosslinked at 65°C for 6 h. The pre-immune complex served as negative control. The ChIP primer sequences are listed in supplementary Table S3. Downregulation of PPARβ/δ in MK cells was performed using a siRNA construct against mouse *Ppar*β*/*δ (L-042751-01, Thermo Scientific), as recommended by the manufacturer (Dharmacon, Thermo Scientific). Briefly, MK cells were seeded in 10-cm dishes and transfected with 25 nM of siRNA in DharmaFECT1 transfection reagent (T-2001-03, Thermo Scientific). MK cells transfected with scrambled siRNA were used as controls. The efficiency of KD was tested by real-time PCR 48 h later (supplementary Fig S5E).

### Immunohistochemistry and immunofluorescence

Src, Ki67 and Keratin 13 staining was performed on tissue sections (5 μm) from paraformaldehyde-fixed, paraffin-embedded skin. Briefly, sections were quenched with 3% H_2_O_2_. Ki67 (Abcam, 15580) staining was performed as described by the manufacturer. For Src and Keratin 13 staining, the antigen-retrieval step was achieved in 0.1 M citrate buffer [pH 6] via microwave. After washing, sections were incubated in normal goat serum 1% for 30 min and probed with Src (Cell Signalling; 2109; 1:250) and Keratin 13 (Abcam; ab92551; 1:100) antibodies overnight at 4°C or at room temperature in blocking buffer, respectively. After washing, sections were incubated for 30 min with anti-rabbit horseradish peroxidase secondary antibody (EnVision+ rabbit-HRP; Dako; K4002). Visualization was performed with diaminobenzidine substrate (Vector Labs) for 14 min before being counterstained with Mayer hematoxylin and mounted. Keratin 10 and Keratin 14 co-staining and N-Cadherin staining were performed as described above with the following modifications. Tissue sections were probed with Keratin 10 (Progen; GP-K10; 1/200), Keratin 14 (Covence; PRB-155P; 1/1000) or N-Cadherin (Millipore; 04-1126; 1/50) overnight at 4°C, washed, and then incubated 30 min at room temperature with Goat anti-guinea-pig A488 (Molecular Probes; A11073; 1/200) and Goat anti-rabbit A568 (Molecular Probes; A21069; 1/1000), for Keratin 10/Keratin 14 co-staining or Goat anti-rabbit A568 (Molecular Probes; A21069; 1/1000) for N-Cadherin staining. After washing, counterstaining was performed with 4′,6-diamidino-2-phenylindole (DAPI) for 5 min at RT.

Double staining for laminin 332 and pan-Cytokeratin was performed from OTC-embedded frozen skin samples. Briefly, sections were defrosted and incubated in acetone for 10 min, washed and blocked for 1 h in 1% normal goat serum/5% bovine serum albumin. Sections were probed overnight at 4°C with anti-laminin 332 (Abcam; 14509; 1:200) and anti-pan-Cytokeratin (BMA-T1341; 1:250) in blocking buffer. After being washed, sections were incubated for 30 min with anti-mouse STAV-Cy3 (Zymed; 43-4315; 1:1800) and anti-rabbit A488 (Molecular Probes; A111034, 1:2000) diluted in blocking buffer. Sections were then washed three times and incubated 5 min with DAPI (Sigma; D95542; 1:5000) and washed again before being mounted.

### PLA and immunofluorescence

PLA experiments were performed in accordance with the manufacturer's protocol (Olink Biosciences, Sweden) using pairs of antibodies targeting the protein–protein interactions of interest. The antibodies were laminin 332 (1:20; Abcam, ab14509), active integrin β4 (Abcam, ab29042; 1/50) and Rac1 (Abcam, ab97732; 1/20). PLA images were taken using the LSM 710 confocal microscope (Carl Zeiss, Germany) with the Plan-Apochromat 40 × /1.4 oil differential interference contrast objective. Analyses were performed with the ZEN 2009 Light Edition software (Carl Zeiss, Germany). Quantification of PLA signals was performed with BlobFinder version 3.2 (Allalou & Wahlby, 2009). Immunofluorescence for laminin 332 (1/250) was performed after PLA for integrin β4 and Rac1 as previously described.

### Epidermis and dermis separation for RNA extraction

Epidermis and dermis were prepared from whole dorsal skin of acutely irradiated mice according to the protocol developed by Clemmensen *et al* (Clemmensen *et al*, 2009), with some modifications. After dorsal skin harvest, adipose tissue was removed with a scalpel on ice, and the skin was cut into small pieces (1–2 mm) and immediately incubated for 15 min at RT in ammonium thiocyanate 3.8% in 1 × phosphate-buffered saline. Epidermis and dermis were then separated mechanically with forceps in TRIzol. After RNA extraction and quality control by Bioanalyser, RT-PCR and qPCR were performed as described below to generate cDNA. Specific markers of the epidermis (Keratins 10 and 14) and dermis (Collagen 4α1) compartments were tested by qRT-PCR to check the proper separation of the two in each experiment (supplementary Fig S2D).

### Statistics summary

Unless indicated otherwise, all data are presented as the means ± standard errors of the mean, and statistical differences were evaluated by two-tailed Student's *t*-tests. For all analyses, we considered *p* < 0.05 to be statistically significant.

### Study approval

Collection of human skin biopsies was approved by the local (Centre Hospitalier Universitaire Vaudois) and cantonal (Canton de Vaud) research ethics committees. All experiments involving animals were approved by the Veterinary Office of the Canton Vaud (Switzerland) in accordance with the Federal Swiss Veterinary Office Guidelines. All experiments conform to European Commission Directive 86/609/EEC.

## References

[b1] Adhikary T, Brandt DT, Kaddatz K, Stockert J, Naruhn S, Meissner W, Finkernagel F, Obert J, Lieber S, Scharfe M (2012). Inverse PPARbeta/delta agonists suppress oncogenic signaling to the ANGPTL4 gene and inhibit cancer cell invasion. Oncogene.

[b2] Allalou A, Wahlby C (2009). BlobFinder, a tool for fluorescence microscopy image cytometry. Comput Methods Programs Biomed.

[b3] Bissell MJ, Radisky D (2001). Putting tumours in context. Nat Rev Cancer.

[b4] Bode AM, Dong Z (2003). Mitogen-activated protein kinase activation in UV-induced signal transduction. Sci STKE.

[b5] Boukamp P (2005). Non-melanoma skin cancer: What drives tumor development and progression?. Carcinogenesis.

[b6] Broders AC (1921). Squamous-cell epithelioma of the skin: A study of 256 cases. Ann Surg.

[b7] Burdick AD, Bility MT, Girroir EE, Billin AN, Willson TM, Gonzalez FJ, Peters JM (2007). Ligand activation of peroxisome proliferator-activated receptor-beta/delta(PPARbeta/delta) inhibits cell growth of human N/TERT-1 keratinocytes. Cell Signal.

[b8] Campbell C, Quinn AG, Rees JL (1993). Codon 12 Harvey-ras mutations are rare events in non-melanoma human skin cancer. Br J Dermatol.

[b9] Cataisson C, Joseloff E, Murillas R, Wang A, Atwell C, Torgerson S, Gerdes M, Subleski J, Gao JL, Murphy PM (2003). Activation of cutaneous protein kinase C alpha induces keratinocyte apoptosis and intraepidermal inflammation by independent signaling pathways. J Immunol.

[b10] Chang HY, Sneddon JB, Alizadeh AA, Sood R, West RB, Montgomery K, Chi JT, Rijn van de M, Botstein D, Brown PO (2004). Gene expression signature of fibroblast serum response predicts human cancer progression: Similarities between tumors and wounds. PLoS Biol.

[b11] Chen C, Mendez E, Houck J, Fan W, Lohavanichbutr P, Doody D, Yueh B, Futran ND, Upton M, Farwell DG (2008). Gene expression profiling identifies genes predictive of oral squamous cell carcinoma. Cancer Epidemiol Biomarkers Prev.

[b12] Clemmensen A, Thomassen M, Clemmensen O, Tan Q, Kruse TA, Petersen TK, Andersen F, Andersen KE (2009). Extraction of high-quality epidermal RNA after ammonium thiocyanate-induced dermo-epidermal separation of 4 mm human skin biopsies. Exp Dermatol.

[b13] Devary Y, Rosette C, DiDonato JA, Karin M (1993). NF-kappa B activation by ultraviolet light not dependent on a nuclear signal. Science.

[b14] Di-Poi N, Tan NS, Michalik L, Wahli W, Desvergne B (2002). Antiapoptotic role of PPARbeta in keratinocytes via transcriptional control of the Akt1 signaling pathway. Mol Cell.

[b15] Ehrhart JC, Gosselet FP, Culerrier RM, Sarasin A (2003). UVB-induced mutations in human key gatekeeper genes governing signalling pathways and consequences for skin tumourigenesis. Photochem Photobiol Sci.

[b16] Farmer P, Bonnefoi H, Anderle P, Cameron D, Wirapati P, Becette V, Andre S, Piccart M, Campone M, Brain E (2009). A stroma-related gene signature predicts resistance to neoadjuvant chemotherapy in breast cancer. Nat Med.

[b17] Fitsialos G, Chassot AA, Turchi L, Dayem MA, LeBrigand K, Moreilhon C, Meneguzzi G, Busca R, Mari B, Barbry P (2007). Transcriptional signature of epidermal keratinocytes subjected to in vitro scratch wounding reveals selective roles for ERK1/2, p38, and phosphatidylinositol 3-kinase signaling pathways. J Biol Chem.

[b18] Fritsche E, Schafer C, Calles C, Bernsmann T, Bernshausen T, Wurm M, Hubenthal U, Cline JE, Hajimiragha H, Schroeder P (2007). Lightening up the UV response by identification of the arylhydrocarbon receptor as a cytoplasmatic target for ultraviolet B radiation. Proc Natl Acad Sci U S A.

[b19] Genini D, Garcia-Escudero R, Carbone GM, Catapano CV (2012). Transcriptional and non-transcriptional functions of PPARbeta/delta in non-small cell lung cancer. PLoS One.

[b20] Glick AB (2012). The role of TGFbeta signaling in squamous cell cancer: Lessons from mouse models. J Skin Cancer.

[b21] Guasch G, Schober M, Pasolli HA, Conn EB, Polak L, Fuchs E (2007). Loss of TGFbeta signaling destabilizes homeostasis and promotes squamous cell carcinomas in stratified epithelia. Cancer Cell.

[b22] Gupta RA, Wang D, Katkuri S, Wang H, Dey SK, DuBois RN (2004). Activation of nuclear hormone receptor peroxisome proliferator-activated receptor-delta accelerates intestinal adenoma growth. Nat Med.

[b23] Hameetman L, Commandeur S, Bavinck JN, Wisgerhof HC, Gruijl de FR, Willemze R, Mullenders L, Tensen CP, Vrieling H (2013). Molecular profiling of cutaneous squamous cell carcinomas and actinic keratoses from organ transplant recipients. BMC Cancer.

[b24] Han G, Lu SL, Li AG, He W, Corless CL, Kulesz-Martin M, Wang XJ (2005). Distinct mechanisms of TGF-beta1-mediated epithelial-to-mesenchymal transition and metastasis during skin carcinogenesis. J Clin Invest.

[b25] Harman FS, Nicol CJ, Marin HE, Ward JM, Gonzalez FJ, Peters JM (2004). Peroxisome proliferator-activated receptor-delta attenuates colon carcinogenesis. Nat Med.

[b26] Hollingshead HE, Borland MG, Billin AN, Willson TM, Gonzalez FJ, Peters JM (2008). Ligand activation of peroxisome proliferator-activated receptor-beta/delta (PPARbeta/delta) and inhibition of cyclooxygenase 2 (COX2) attenuate colon carcinogenesis through independent signaling mechanisms. Carcinogenesis.

[b27] Hoot KE, Lighthall J, Han G, Lu SL, Li A, Ju W, Kulesz-Martin M, Bottinger E, Wang XJ (2008). Keratinocyte-specific Smad2 ablation results in increased epithelial-mesenchymal transition during skin cancer formation and progression. J Clin Invest.

[b28] Joseloff E, Cataisson C, Aamodt H, Ocheni H, Blumberg P, Kraker AJ, Yuspa SH (2002). Src family kinases phosphorylate protein kinase C delta on tyrosine residues and modify the neoplastic phenotype of skin keratinocytes. J Biol Chem.

[b29] Kannan-Thulasiraman P, Seachrist DD, Mahabeleshwar GH, Jain MK, Noy N (2010). Fatty acid-binding protein 5 and PPARbeta/delta are critical mediators of epidermal growth factor receptor-induced carcinoma cell growth. J Biol Chem.

[b30] Kersten S, Mandard S, Tan NS, Escher P, Metzger D, Chambon P, Gonzalez FJ, Desvergne B, Wahli W (2000). Characterization of the fasting-induced adipose factor FIAF, a novel peroxisome proliferator-activated receptor target gene. J Biol Chem.

[b31] Kim DJ, Akiyama TE, Harman FS, Burns AM, Shan W, Ward JM, Kennett MJ, Gonzalez FJ, Peters JM (2004). Peroxisome proliferator-activated receptor beta (delta)-dependent regulation of ubiquitin C expression contributes to attenuation of skin carcinogenesis. J Biol Chem.

[b32] Kopetz S, Shah AN, Gallick GE (2007). Src continues aging: Current and future clinical directions. Clin Cancer Res.

[b33] Lieu FM, Yamanishi K, Konishi K, Kishimoto S, Yasuno H (1991). Low incidence of Ha-ras oncogene mutations in human epidermal tumors. Cancer Lett.

[b34] Lohavanichbutr P, Mendez E, Holsinger FC, Rue TC, Zhang Y, Houck J, Upton MP, Futran N, Schwartz SM, Wang P (2013). A 13-gene signature prognostic of HPV-negative OSCC: Discovery and external validation. Clin Cancer Res.

[b35] Madan V, Lear JT, Szeimies RM (2010). Non-melanoma skin cancer. Lancet.

[b36] Martins VL, Vyas JJ, Chen M, Purdie K, Mein CA, South AP, Storey A, McGrath JA, O'Toole EA (2009). Increased invasive behaviour in cutaneous squamous cell carcinoma with loss of basement-membrane type VII collagen. J Cell Sci.

[b37] Matsumoto T, Jiang J, Kiguchi K, Ruffino L, Carbajal S, Beltran L, Bol DK, Rosenberg MP, DiGiovanni J (2003). Targeted expression of c-Src in epidermal basal cells leads to enhanced skin tumor promotion, malignant progression, and metastasis. Cancer Res.

[b38] Matsumoto T, Kiguchi K, Jiang J, Carbajal S, Ruffino L, Beltran L, Wang XJ, Roop DR, DiGiovanni J (2004). Development of transgenic mice that inducibly express an active form of c-Src in the epidermis. Mol Carcinog.

[b39] Matsumura Y, Ananthaswamy HN (2002). Short-term and long-term cellular and molecular events following UV irradiation of skin: Implications for molecular medicine. Expert Rev Mol Med.

[b40] Matsumura Y, Ananthaswamy HN (2004). Toxic effects of ultraviolet radiation on the skin. Toxicol Appl Pharmacol.

[b41] Michalik L, Desvergne B, Tan NS, Basu-Modak S, Escher P, Rieusset J, Peters JM, Kaya G, Gonzalez FJ, Zakany J (2001). Impaired skin wound healing in peroxisome proliferator-activated receptor (PPAR)alpha and PPARbeta mutant mice. J Cell Biol.

[b42] Michalik L, Desvergne B, Wahli W (2004). Peroxisome-proliferator-activated receptors and cancers: Complex stories. Nat Rev Cancer.

[b43] Michalik L, Feige JN, Gelman L, Pedrazzini T, Keller H, Desvergne B, Wahli W (2005). Selective expression of a dominant-negative form of peroxisome proliferator-activated receptor in keratinocytes leads to impaired epidermal healing. Mol Endocrinol.

[b44] Montagner A, Rando G, Degueurce G, Leuenberger N, Michalik L, Wahli W (2011). New insights into the role of PPARs. Prostaglandins Leukot Essent Fatty Acids.

[b45] Muller-Brusselbach S, Komhoff M, Rieck M, Meissner W, Kaddatz K, Adamkiewicz J, Keil B, Klose KJ, Moll R, Burdick AD (2007). Deregulation of tumor angiogenesis and blockade of tumor growth in PPARbeta-deficient mice. EMBO J.

[b46] Nakamura M, Tokura Y (2011). Epithelial-mesenchymal transition in the skin. J Dermatol Sci.

[b47] Newkirk KM, Parent AE, Fossey SL, Choi C, Chandler HL, Rajala-Schultz PJ, Kusewitt DF (2007). Snai2 expression enhances ultraviolet radiation-induced skin carcinogenesis. Am J Pathol.

[b48] Nijsten T, Geluyckens E, Colpaert C, Lambert J (2005). Peroxisome proliferator-activated receptors in squamous cell carcinoma and its precursors. J Cutan Pathol.

[b49] Nuyten DS, Vijver van de MJ (2006). Gene expression signatures to predict the development of metastasis in breast cancer. Breast Dis.

[b50] Ooi EM, Watts GF, Sprecher DL, Chan DC, Barrett PH (2011). Mechanism of action of a peroxisome proliferator-activated receptor (PPAR)-delta agonist on lipoprotein metabolism in dyslipidemic subjects with central obesity. J Clin Endocrinol Metab.

[b51] Park BH, Vogelstein B, Kinzler KW (2001). Genetic disruption of PPARdelta decreases the tumorigenicity of human colon cancer cells. Proc Natl Acad Sci U S A.

[b52] Peters JM, Gonzalez FJ (2009). Sorting out the functional role(s) of peroxisome proliferator-activated receptor-beta/delta (PPARbeta/delta) in cell proliferation and cancer. Biochim Biophys Acta.

[b53] Peters JM, Shah YM, Gonzalez FJ (2012). The role of peroxisome proliferator-activated receptors in carcinogenesis and chemoprevention. Nat Rev Cancer.

[b54] Pierceall WE, Goldberg LH, Tainsky MA, Mukhopadhyay T, Ananthaswamy HN (1991). Ras gene mutation and amplification in human nonmelanoma skin cancers. Mol Carcinog.

[b55] Reed KR, Sansom OJ, Hayes AJ, Gescher AJ, Winton DJ, Peters JM, Clarke AR (2004). PPARdelta status and Apc-mediated tumourigenesis in the mouse intestine. Oncogene.

[b56] Rho O, Kim DJ, Kiguchi K, Digiovanni J (2011). Growth factor signaling pathways as targets for prevention of epithelial carcinogenesis. Mol Carcinog.

[b57] Rieck M, Meissner W, Ries S, Muller-Brusselbach S, Muller R (2008). Ligand-mediated regulation of peroxisome proliferator-activated receptor (PPAR) beta/delta: A comparative analysis of PPAR-selective agonists and all-trans retinoic acid. Mol Pharmacol.

[b58] Romanowska M, Reilly L, Palmer CN, Gustafsson MC, Foerster J (2010). Activation of PPARbeta/delta causes a psoriasis-like skin disease in vivo. PLoS One.

[b59] Rowert-Huber J, Patel MJ, Forschner T, Ulrich C, Eberle J, Kerl H, Sterry W, Stockfleth E (2007). Actinic keratosis is an early in situ squamous cell carcinoma: A proposal for reclassification. Br J Dermatol.

[b60] Schafer M, Werner S (2008). Cancer as an overhealing wound: An old hypothesis revisited. Nat Rev Mol Cell Biol.

[b61] Schug TT, Berry DC, Shaw NS, Travis SN, Noy N (2007). Opposing effects of retinoic acid on cell growth result from alternate activation of two different nuclear receptors. Cell.

[b62] Schug TT, Berry DC, Toshkov IA, Cheng L, Nikitin AY, Noy N (2008). Overcoming retinoic acid-resistance of mammary carcinomas by diverting retinoic acid from PPARbeta/delta to RAR. Proc Natl Acad Sci U S A.

[b63] Seo HR, Kwan YW, Cho CK, Bae S, Lee SJ, Soh JW, Chung HY, Lee YS (2004). PKCalpha induces differentiation through ERK1/2 phosphorylation in mouse keratinocytes. Exp Mol Med.

[b64] Serrels B, Serrels A, Mason SM, Baldeschi C, Ashton GH, Canel M, Mackintosh LJ, Doyle B, Green TP, Frame MC (2009). A novel Src kinase inhibitor reduces tumour formation in a skin carcinogenesis model. Carcinogenesis.

[b65] Sertznig P, Seifert M, Tilgen W, Reichrath J (2008). Peroxisome proliferator-activated receptors (PPARs) and the human skin: Importance of PPARs in skin physiology and dermatologic diseases. Am J Clin Dermatol.

[b66] Tan NS, Icre G, Montagner A, Bordier-ten-Heggeler B, Wahli W, Michalik L (2007). The nuclear hormone receptor peroxisome proliferator-activated receptor beta/delta potentiates cell chemotactism, polarization, and migration. Mol Cell Biol.

[b67] Tan NS, Michalik L, Di-Poi N, Ng CY, Mermod N, Roberts AB, Desvergne B, Wahli W (2004). Essential role of Smad3 in the inhibition of inflammation-induced PPARbeta/delta expression. EMBO J.

[b68] Tan NS, Michalik L, Noy N, Yasmin R, Pacot C, Heim M, Fluhmann B, Desvergne B, Wahli W (2001). Critical roles of PPAR beta/delta in keratinocyte response to inflammation. Genes Dev.

[b69] Trifan OC, Hla T (2003). Cyclooxygenase-2 modulates cellular growth and promotes tumorigenesis. J Cell Mol Med.

[b70] Wang D, Wang H, Guo Y, Ning W, Katkuri S, Wahli W, Desvergne B, Dey SK, DuBois RN (2006). Crosstalk between peroxisome proliferator-activated receptor delta and VEGF stimulates cancer progression. Proc Natl Acad Sci U S A.

[b71] Wang HQ, Smart RC (1999). Overexpression of protein kinase C-alpha in the epidermis of transgenic mice results in striking alterations in phorbol ester-induced inflammation and COX-2, MIP-2 and TNF-alpha expression but not tumor promotion. J Cell Sci.

[b72] Westergaard M, Henningsen J, Johansen C, Rasmussen S, Svendsen ML, Jensen UB, Schroder HD, Staels B, Iversen L, Bolund L (2003). Expression and localization of peroxisome proliferator-activated receptors and nuclear factor kappaB in normal and lesional psoriatic skin. J Invest Dermatol.

[b73] Yagi R, Waguri S, Sumikawa Y, Nada S, Oneyama C, Itami S, Schmedt C, Uchiyama Y, Okada M (2007). C-terminal Src kinase controls development and maintenance of mouse squamous epithelia. EMBO J.

[b74] Yoshinaga M, Taki K, Somada S, Sakiyama Y, Kubo N, Kaku T, Tsuruta S, Kusumoto T, Sakai H, Nakamura K (2011). The expression of both peroxisome proliferator-activated receptor delta and cyclooxygenase-2 in tissues is associated with poor prognosis in colorectal cancer patients. Dig Dis Sci.

[b75] Zuo X, Peng Z, Moussalli MJ, Morris JS, Broaddus RR, Fischer SM, Shureiqi I (2009). Targeted genetic disruption of peroxisome proliferator-activated receptor-delta and colonic tumorigenesis. J Natl Cancer Inst.

