# Supplementary material for: Src is activated by the nuclear receptor peroxisome proliferator-activated receptor β/δ in ultraviolet radiation-induced skin cancer
Source: EMBO Mol Med. 2013 Nov 6;6(1):80–98. doi: 10.1002/emmm.201302666 (PMC3936491; doi:10.1002/emmm.201302666)
Supplement: Supplementary file 1 [file emmm0006-0080-sd1.pdf]

EMBO Molecular Medicine

Src is activated by the nuclear receptor peroxisome proliferator-activated receptor β/δ in ultraviolet radiation-induced skin cancer

Alexandra Montagner, Maria B Delgado, Corinne Tallichet-Blanc, Soon Kiat Jeremy Chan, Ming Keat Sng, Hélène Mottaz, Gwendoline Degueurce, Yannick Lippi, Catherine Moret, Michael Baruchet, Maria Antsiferova, Sabine Werner, Daniel Hohl, Talal Al Saati, Pierre J. Farmer, Nguan Soon Tan, Liliane Michalik and Walter Wahli

Corresponding author: Walter Wahli, University of Lausanne

Review timeline:

Submission date:	20 February 2013
Editorial Decision:	25 March 2013
Revision received:	23 August 2013
Editorial Decision:	12 September 2013
Revision received:	25 September 2013
Accepted:	26 September 2013

Transaction Report:

(Note: With the exception of the correction of typographical or spelling errors that could be a source of ambiguity, letters and reports are not edited. The original formatting of letters and referee reports may not be reflected in this compilation.)

Editor: Roberto Buccione

1st Editorial Decision 25 March 2013

Thank you for the submission of your manuscript to EMBO Molecular Medicine. We have now received reports from the three Reviewers whom we asked to evaluate your manuscript.

You will see that while two out of three Reviewers are generally supportive, they all raise critical points that question the impact and conclusiveness of the results, thus preventing us from considering publication at this time.

Reviewer 1 challenges your interpretation that stimulation of TGF-beta1 by UV in wild type but not in PPARbeta/delta null mice indicates activation of the receptor. S/he suggests that it is possible that loss of the receptor might affect other pathways, which in turn would impinge on TGF-beta1 expression. Reviewer 1 also notes that the changes shown in Figure 6 would suggest EMT and would like to you to verify if this is indeed confirmed in vivo. Reviewer 1 lists other important issues, which also require your action.

Reviewer 2 feels that a number of problems need to be addressed to consolidate your data and thus conclusions. Indeed, s/he suggests that the main contention that cSrc is a direct target of PPARbeta/delta in keratinocytes requires ChIP and direct demonstration that cSrc transcription is activated by PPARbeta/delta engagement in the absence of protein synthesis. Reviewer 2 also mentions that the c-Src (as opposed to other Src-family members) specificity is an interesting point and should be further analysed. In addition, s/he notes that the tumour data should be integrated with

appropriate histological and immunohistochemistry analysis. Reviewer 1 also lists other important issues, on which you should act upon.

Reviewer 3 is mostly negative, based on his/her perception that your findings are not sufficiently novel. As Reviewers 1 and 2 do not appear to share the same concern, I am willing to overlook this objection, provided you provide a reply for each point and comply with his/her other concerns. Reviewer 3 in fact, and similarly to Reviewer 2, does point out that a more comprehensive analysis should be carried out to confirm your conclusion that cSrc is a direct target of PPARbeta/delta.

While publication of the paper cannot be considered at this stage, we would be prepared to consider a suitably revised submission, with the understanding that the Reviewer's concerns must be fully addressed with additional experimental data, where appropriate. Your revised the manuscript will undergo a second round of review.

Please note that it is EMBO Molecular Medicine policy to allow a single round of revision only and that, therefore, acceptance or rejection of the manuscript will depend on the completeness of your responses included in the next, final version of the manuscript.

I look forward to seeing a revised form of your manuscript as soon as possible.

***** Reviewer's comments *****

Referee #1 (Comments on Novelty/Model System):

This paper shows that Src is activated by PPARbeta/delta which promotes accelerated UV-induced skin cancer in mice. It is interesting, but some of the data is not sufficiently convincing to recommend publication.

Referee #1 (Remarks):

This paper shows that Src is activated by PPARbeta/delta which promotes accelerated UV-induced skin cancer in mice.

Comments: Results, first paragraph. The authors state that stimulation of TGF-beta1 by UV in wild type but not in PPARbeta/delta null mice indicates activation of the receptor. Although suggestive, it is possible that loss of the receptor might affect other pathways which would also affect TGF-beta1 expression.
Figure 1: Although the results in Figure 1 definitely suggest that tumourigenesis is enhanced in the wild-type mice compared to the null mice, there is a catch-up effect, probably activation of a compensatory pathway and it would be interesting to know what this is. In Figure 1F, I was surprised that so little TGF-beta1 is detectable in the dermis.
Figure 2: Panel B, right: Do the quantification numbers refer to the Src blot (I presume so) or the Tyr416 blot. The latter is of poor quality and needs to be improved. Panel E, looks much better in black and white then it does in colour ie the increased Src staining is evident in the basal layer.
Figure 3: The title of Figure 3 suggests that the authors have shown regulation of Src expression in skin, but the luciferase assays were performed in NIH3T3 cells.
Figure 4: This Figure is generally not of good enough quality for publication. The phospho-EGFR Westerns are of poor quality and even the ERK1/2 Westerns are variable as is the EGFR IB in panel D. Why does PPAR-Beta/Delta KD have no effect on Src expression. The molecular weight of the EGFR increases in the UV lanes suggesting post-translational modification.
Figure 5: Again, the phospho-Src and p-EGFR blots are of poor quality.
Figure 6: The changes shown are suggestive of EMT and it would be interesting if the authors could show this in their in vivo model ie in the mice tumours. The ataining changes in laminin-332 suggest degradation rather than increased invasion ie the BMZ is still continuous, but there is decreased expression of laminin-332. MMP-19 has been shown to target the gamma2 chain of laminin-332.
Figure 7: Why is the data shown for MMP19 rather than TGFB1 in Figure 7B.

Referee #2 (Remarks):

This is an interesting paper, reporting on transcriptional control of the c-Src gene by the nuclear receptor PPARß/δ in the context of skin squamous cell cancer development. The findings are interesting and overall convincing. There are a few issues that need to be addressed :

1) The major conclusion that c-Src is direct gene target of PPARß/δ in keratinocytes needs to better supported. Chromatin Immunoprecitiation assays demonstrating the binding of endogenous PPARß/δ to the c-Src gene are necessary, as well as the demonstration that c-Src gene transcription occurs in keratinocytes upon PPARß/δ ligand activation in the absence of protein synthesis.
2) As the authors point out in the discussion, little is known on transcriptional control of the c-Src gene. Based on the data that they provide, a possibility to be considered is that at least some of the effects that they observe on c-Src expression are mediated by activation of TGF-ß/Smad signaling. In addition, the various EMT markers that were examined are also well known TGF-ß/Smad targets. These possibilities that need to considered in the interpretation / discussion of the data.
3) The specificity of effects on c-Src gene expression, versus other family members (Fyn, Yes) is very interesting and should be shown. Is the transcription regulatory region of Fyn and Yes devoid of PPARß/δ binding sites as determined by bioinformatics analysis ?
4) For the expression data shown in Fig. 1A, only mice at 31 weeks of UV exposure were analyzed. It would be desirable to include in the analysis early times, especially considering that 100% of mice develop tumors by 22 weeks.
5) Representative histological and immunohistochemical images of tumors are necessary, together with an adequate assessment (grading) of tumor cell differentiation. It is also important to determine how many of the UV-induced spindle cell tumors are positive for keratin expression, as keratin-negative tumors could also be mesenchymally-derived (sarcomas).
6) The existence of a positive feedback loop between c-Src and PPARß/δ expression and activity is very interesting and supporting data should be shown as an integral part of the paper, rather than as supplemental material.
7) In the analysis of various tumor types shown in Fig. 7C, skin or oral SCC data would have to be included.

Referee #3 (Comments on Novelty/Model System):

The involvement of PPARdelta in oncogenic activities and the role of the receptor in EGFR-induced cancer cell proliferation were previously documented. The authors point at a controversy in the literature but the cited references suggest that while several groups reported that PPARdelta exerts pro-oncogenic activities, only one group suggested the opposite.

Referee #3 (Remarks):

Work described in this manuscript investigated the involvement of the nuclear receptor PPARd in UV-induced skin cancer. The data indicate that : 1) UV irradiation induced the expression of PPARd and two known PPARd target genes in mouse skin; 2) that UV-induced skin tumor formation was delayed in PPARd-null vs. WT mice; 3) activation of PPARd in keratinocytes resulted in increased expression of Src, leading to activation of EGFR and ERK1/2, 4) skins of UV-irradiated PPARd-null mice displayed lower expression of markers of proliferation and invasion, and 5) a correlation between the expression levels of PPARd and Src was found in various human tumors. Overall, the data support previous observations from this and other groups indicating that PPARd exerts pro-proliferative and oncogenic activities in some cells.

Comments:

- The involvement of PPARd in oncogenic activities was previously reported. The main novel finding of the present work is the identification of Src as a direct target gene for this receptor. In addition to the reporter transactivation assays, a more comprehensive analysis should be carried out to confirm this conclusion. Does PPARd indeed target Src directly, i.e. without the need of de novo protein synthesis? is the receptor associated with the Src promoter in cells?
- The involvement of PPARd in induction carcinoma cell growth by EGFR was previously reported (JBC 2010 285:19106). These findings should be discussed.

- Considering that ablation of PPARd somewhat delayed but did not prevent UV-induced tumor development in mice (Fig. 1B), the authors' suggestion that this receptor may comprise a target for anti-cancer compounds is a stretch.

1st Revision - authors' response 23 August 2013

Point-by-point reply to the reviewers' remarks for EMM-2013_02666

We thank the three referees for their comments. They have triggered many additional experiments, which have generated a lot of new data that have improved and enriched our work.

Referee #1 (Comments on Novelty/Model System):
This paper shows that Src is activated by PPARbeta/delta which promotes accelerated UV-induced skin cancer in mice. It is interesting, but some of the data is not sufficiently convincing to recommend publication.

Referee #1 (Remarks):
Comments: Results, first paragraph. The authors state that stimulation of TGF-beta1 by UV in wild type but not in PPARbeta/delta null mice indicates activation of the receptor. Although suggestive, it is possible that loss of the receptor might affect other pathways, which would also affect TGF-beta1 expression.

Reply

It is correct that other pathways could affect TGF-beta1 expression. In our interpretation in which PPARb/d gene induction upon chronic UV exposure is correlated with an increase in nuclear receptor activity, we now demonstrate that the known PPARb/d target gene *Plin2* is also up-regulated upon UV exposure (new Fig 1A). Furthermore, we show in this revised version of the manuscript that *Tgfb1* is a target gene of PPARb/d in keratinocytes, regulated by a PPRE in its 5' upstream region (see below and Supporting Information Fig S6).

In fact, the regulation of *Tgfb1* expression has been addressed in several studies that have identified an activator protein 1 (AP-1) and a CRE binding site in its promoter region. The AP-1 site appears to be the most important element in the transcriptional regulation of *Tgfb1*, notably in response to cytokines such as IL-1b, TNFa, or IL-13 (Lee et al, 2001; Warshamana et al, 2001). Moreover, an autoinduction of *Tgfb1 via* this AP-1 site has been proposed, as well (Kim et al, 1990). MAPK Erk1/2 and JNK appear as major regulators of *Tgfb1* expression because they control the phosphorylation and formation of the c-Jun and c-Fos complex, known as the AP-1 transcription factor. As seen in this study, *Pparb/d*$^{-/-}$ mice display a defect in the activation of MAPK Erk1/2. In addition, microarray data, which we have collected in the context of a different unpublished study, also show a down-regulation of GTPases, notably RhoGTPases, suggesting a reduced JNK activity.

Moreover, Kim *et al* have shown that *Tgfb1* is a direct target of PPARb/d in rat aortic vascular smooth muscle cells (Kim et al, 2008). To test whether PPARb/d also regulates *Tgfb1* in mouse keratinocytes, we have identified *in silico* two PPAR response elements (PPREs) in the mouse *Tgfb1* regulatory region. We then performed ChIP with a PPARb/d antibody and then re-ChIP against p300, a co-activator of PPARb/d, to define the ability of activated PPARb/d to bind these putative response elements. These experiments were conducted in a mouse keratinocyte in which *Pparb/d* is down-regulated by transfection with a siRNA construct against mouse *Pparβ/δ* (knockdown cells). Scrambled siRNA (wild-type cells) was used as a control. Results presented in Supporting Information Fig S6 show that activated PPARb/d can bind the PPRE1 of the *Tgfb1* promoter in a GW501516 treatment-dependent manner, indicating that *Tgfb1* is a direct PPARb/d target gene in keratinocytes.

Figure 1: Although the results in Figure 1 definitely suggest that tumourigenesis is enhanced in the wild-type mice compared to the null mice, there is a catch-up effect, probably activation of a compensatory pathway and it would be interesting to know what this is. In Figure 1F, I was surprised that so little TGF-beta1 is detectable in the dermis.

Reply

As mentioned by the reviewer, a catch-up effect is observed in *Pparb/d*$^{-/-}$ mice after 22 weeks of UV chronic exposure, a point also noted by Reviewer 2. To identify dysregulation in signaling pathways that explain this catch-up effect observed in *Pparb/d*$^{-/-}$, the following approach may be suggested. The first step would be RNA profiling in both wild-type and *Pparb/d*$^{-/-}$ mice at weeks 0, 12, and 22. Changes in the expression profiles should be studied together with gene mutations occurring during UV exposure. Such an approach might help to explain the catch-up effect but clearly represents a study on its own and is beyond the scope of the current work.

The *Tgfb1* expression level in the dermis compartment was very similar in all the experiments that we conducted after epidermis-dermis separation. In fact, we consistently observed a higher mRNA expression level in the epidermis compartment compared to dermis with or without UV exposure. In a tentative complement to the epidermis-dermis separation experiment, we also performed IHC experiments. Unfortunately, commercially available antibodies directed against mouse TGFb1 or the anti-mouse TGFb1 antibody produced in Dr. Wakefield's laboratory did not provide conclusive results because we have been unable to overcome nonspecific signals in the negative controls. However, we think that the results obtained after separation of dermis and epidermis are quite convincing.

Figure 2: Panel B, right: Do the quantification numbers refer to the Src blot (I presume so) or the Tyr416 blot. The latter is of poor quality and needs to be improved. Panel E, looks much better in black and white then it does in colour ie the increased Src staining is evident in the basal layer.

Reply

As observed by the reviewer, the quantification presented in Fig 2B refers to total Src. This is now indicated in the legend of Figure 2B.

To improve phospho-Tyr416 Src IB quality, we have tested a new antibody (rabbit monoclonal phospho-Src family (Tyr416) (D49G4) #6943 instead of rabbit monoclonal phospho-Src family (Tyr416) (100F9) #2113, both from Cell Signaling) on total cell lysates and after Src immunoprecipitation (Millipore #OP07). Unfortunately, the quality of the IB was not really improved. However, to answer the reviewer's legitimate question satisfactorily, we argue that the increase in phospho-Tyr845 EGFR, which reflects Src kinase activity (Biscardi et al, 1999), confirms a higher Src activity (Figures 4 and 5). In turn, we decided not to show IBs directed against phosphorylated Src in this revised version because their quality was not satisfactory. The Results and Material & Methods sections have been modified accordingly.

We agree that black-and-white pictures could increase intensity of the Src staining in the basal layer of the epidermis. However, in such pictures, the counterstaining with Mayer hematoxylin, which labels nuclei and allowed us to localize Src, may confuse the reader, who might conclude that nuclear Src is present in all cells. Therefore, we would prefer to present the colored version of those pictures. We hope the reviewer understands.

Figure 3: The title of Figure 3 suggests that the authors have shown regulation of Src expression in skin, but the luciferase assays were performed in NIH3T3 cells.

Reply

In the first version of the manuscript, the title of Figure 3 referred to *in vivo*, but the data presented are mainly from *in cellulo* experiments. We have corrected this mistake. Now Figure 3 also comprises the ChIP with anti-PPARb/d followed by re-ChIP with anti-p300 experiments. These results show that activated PPARb/d directly interacts with PPREs 3, 4 and 5, previously identified in the *Src* promoter. Because of the new results, we propose to change the previous title of Figure 3 to "*Src* is a direct PPARb/d target gene".

Figure 4: This Figure is generally not of good enough quality for publication. The phospho-EGFR Westerns are of poor quality and even the ERK1/2 Westerns are variable as is the EGFR IB in panel D. Why does PPAR-Beta/Delta KD have no effect on Src expression. The molecular weight of the EGFR increases in the UV lanes suggesting post-translational modification.

Reply

To increase the overall quality of the IBs, they were redone on the same protein extracts with different antibodies. The antibodies against total and phospho-Tyr845 EGFR are now rabbit monoclonal antibodies directed against phospho-EGFR (Tyr845) (D63B4) #6963 and EGF Receptor

(D38B1) #4267 from Cell Signaling. They were used instead of phospho-Tyr845 EGFR #06-847 and EGFR #04-283 from Merck Millipore and allowed us to increase protein detection especially on mouse skin protein extracts for EGFR. The IBs presented in Figure 4B, C, and D and in Figure 5E have been replaced by these new versions.

The aim of the experiment presented in Figure 4A was to determine whether the EGFR/Erk1/2 pathway is stimulated after a short UVB exposure in a PPARb/d- and/or Src-dependent manner in keratinocytes and to identify the impact of increased PPARb/d activity on this signaling pathway. In this experimental design, HaCaT cells were transfected with siRNA directed against *Pparb/d* or *Src* and serum-starved for 24 h with or without a PPARb/d agonist (GW501516). They were then subjected to a unique UVB dose (40 mJ/cm^2) and harvested 30 min later. As the reviewer noted, Src protein levels were not decreased in DMSO-treated HaCaT cells transfected with the siRNA targeting PPARb/d and subjected to UVB irradiation. However, compared to the DMSO control, Src protein levels were higher in GW501516-treated cells, and these levels were significantly reduced in cells transfected with the Pparb/d siRNA. This suggests that PPARb/d activation is needed for increasing Src expression, leading to an enhanced EGFR/MAPK Erk1/Erk2 activation, which occurred only when cells were pre-treated for 24 h with GW501516.

As the reviewer also noted, the molecular weight of EGFR increases upon UV exposure in almost all conditions, suggesting post-translational modifications. Even if we are mainly interested in phosphorylation of Tyr845, known as a specific Src target residue in EGFR, which is necessary for its full activation (Biscardi et al, 1999), EGFR can also be phosphorylated on other Tyr or Ser/Thr residues. These phosphorylation events could induce its activation and that of its downstream signaling pathways such as the PLCg1 or MAPK (Emlet et al, 1997; Rojas et al, 1996; Zwick et al, 1999). Alternatively, some of them can also result in down-regulation of its activity by promoting binding of proteins involved, for instance, in its ubiquitination (Ettenberg et al, 1999; Levkowitz et al, 1999). As we know from the literature, UVB induces a rapid phosphorylation of EGFR *in cellulo* and *in vivo* followed by the activation of its downstream signaling pathways *via* phosphorylation events. Indeed, it has been proposed that reactive oxygen species production, induced by UVB radiation, can disturb the activity of protein tyrosine phosphatases involved in the EGFR down-regulation (Xu et al, 2006) or by producing a metabolite of tryptophan that can activate Src (Fritsche et al, 2007). Therefore, several phosphorylation events can lead to an increase in EGFR molecular weight, as seen in western blots.

Figure 5: Again, the phospho-Src and p-EGFR blots are of poor quality.

Reply

As previously mentioned, the phospho-Tyr416 Src blot was removed and immunoblotting against total and phospho-Tyr845 EGFR was performed again using the abovementioned antibodies on the same protein extracts and the results integrated into Fig 5E.

Figure 6: The changes shown are suggestive of EMT and it would be interesting if the authors could show this in their *in vivo* model *ie* in the mice tumours. The staining changes in laminin-332 suggest degradation rather than increased invasion *ie* the BMZ is still continuous, but there is decreased expression of laminin-332. MMP-19 has been shown to target the gamma2 chain of laminin-332.

Reply

Laminin 332 is composed of three chains: a3, b3, and g2. As mentioned by the reviewer, the laminin 332 g2 and a3 chains can be subjected to proteolytic processes in normal and tumoral conditions, which can also lead to functional signal transduction, enhancing or preventing keratinocyte migration (for review Marinkovich, 2007). Among them, MMP19 and MMP12, whose gene expression is increased in actinic keratosis with moderate atypia of wild-type mice compared to *Pparb/d*$^{-/-}$, have been described in g2 and a3 cleavage, respectively. Concerning MMP19-dependent g2 cleavage, it has been proposed that this mechanism could lead to increased keratinocyte migration properties and correlate with skin SCC formation (Sadowski et al, 2005).

To know whether laminin 332 staining, observed within the epidermis of wild-type mice papilloma, identified functional protein in terms of ability to enhance signal transduction, we performed an *in situ* proximity ligation assay (PLA). Briefly, this experiment allows highlighting protein-protein interaction on frozen or paraffin-embedded tissue sections by fluorescence. It is based on the use of two primary antibodies specific to each partner raised in different species and species-specific secondary antibodies, called PLA probes, linked to a unique short DNA strand. When the PLA probes are in close proximity (<40 nm), the DNA strands can interact through two

circle-forming DNA oligonucleotides added to the samples. Interacting molecules are amplified *via* the rolling circle process using a polymerase, after an enzymatic ligation step. Labeled complementary oligonucleotide probes then serve to highlight the amplified product, which becomes visible as a distinct bright dot by fluorescence microscopy.

It is well established that laminin 332 is a ligand for a6/b4 and a3/b1 integrins, promoting the formation of two different types of attachment structures: stable anchoring contacts and focal adhesion, respectively (Carter et al, 1990; Carter et al, 1991). Whereas the binding of laminin 332 to b4 promotes Rac1 activation and directional sensing (Pullar et al, 2006; Russell et al, 2003), binding to b1 integrin promotes RhoA-driven non-directional sensing. Numerous studies have shown an essential role for laminin 332 binding to a6/b4 integrin in squamous cell carcinoma progression from various organs by promoting cell migration and invasion (for reviews, Marinkovich, 2007; Tsuruta et al, 2008). In skin squamous cell carcinoma, enhanced expression of the a6/b4 integrin complex is linked to malignant progression in mouse and human, suggesting that a6/b4 can serve as an early predictive marker to identify benign squamous tumors at high risk for malignant progression (Dajee et al, 2003; Savoia et al, 1994; Tennenbaum et al, 1992; Tennenbaum et al, 1993; Tennenbaum et al, 1996). Although a6/b4 is restricted to the basal surface of the basal layer keratinocytes in normal skin, this expression pattern is disrupted early in the development of skin squamous cancer. In fact, benign actinic keratoses with a higher risk for malignant progression express a6/b4 surprabasally. Interestingly, this aberrant suprabasal expression profile of a6/b4 is associated with abnormal expression of Keratin 13, a cytokeratin that is used as an early marker of skin SCC progression susceptibility in actinic keratosis (Darwiche et al, 2007; Larcher et al, 1992; Nischt et al, 1988; Sutter et al, 1993; Tennenbaum et al, 1993).

In the first version of the manuscript, we used the term “papilloma” for structures histologically defined as actinic keratosis, which are classified as grade I, II, or III based on the degree of cytological atypia of epidermal keratinocytes and involvement of adnexal structures according to Rowert-Huber *et al* (Rowert-Huber et al, 2007). In this revised version, we now use the term “actinic keratosis” to be more precise.

We performed the PLA assay first using antibodies directed against b4/laminin 332 to determine whether laminin 332 observed in the suprabasal layer of wild-type actinic keratosis with moderate atypia can interact with a6b4 integrin, and then with antibodies that recognize b4 and Rac1 coupled to a laminin 332 staining to determine whether the b4 integrin identified in the suprabasal region can enhance efficient signal transduction. In the revised version of the manuscript, PLA results for Integrin b4/laminin 332 and Integrin b4/Rac1 coupled with IHC laminin 332 are shown in Figure 6B and C, respectively. For the Integrin b4/laminin 332 interaction (red dots), it is shown that the b4 integrin and laminin 332 interact in the basal layer of the epidermis. Some red dots were observed in the suprabasal layers of the epidermis, suggesting interaction also in these layers. In actinic keratosis with moderate atypia of *Pparb/d*$^{-/-}$ mice, staining is observed mostly in the keratinocytes of the basal layer and less in the suprabasal layers. Interestingly, PLA for b4/Rac1 coupled with laminin 332 staining in Figure 6C revealed transductionally active b4 integrin in the vicinity of laminin 332 staining in both genotypes. However, quantification of the red dots, which reflects the average number of PLA signals per nucleus per sample, showed many more interactions between b4 and laminin 332 in actinic keratosis from wild-type mice compared to *Pparb/d*$^{-/-}$ mice. Altogether, these new results show that keratinocytes of *Pparb*$^{+/+}$ benign tumors display more functional b6/laminin 332 complexes, which are functionally active in terms of signal transduction, compared to *Pparb/d*$^{-/-}$ mice. These data suggest that keratinocytes of wild-type tumors are likely more prone to invade the surrounding stroma.

In support of this observation, immunohistochemistry of Keratin 13 shows that it is highly expressed in actinic keratosis samples from wild-type mice compared to those from *Pparb/d*$^{-/-}$ mice (Supporting Information Fig S10B), suggesting that actinic keratoses of wild-type mice are more prone to progress into SCC. These new data are now presented on Pages 10 and 11.

With respect to the expression of EMT markers in carcinomas, grade II SCCs from *Pparb/d*$^{+/+}$ and *Pparb/d*$^{-/-}$ mice have been studied. Such carcinomas could be obtained from both genotypes, while grade III SCCs were identified only in *Pparb/d*$^{+/+}$ mice. The results obtained are presented in Supporting Information Figure S13. They show that most of the tested markers are expressed at higher levels in grade II SCC of wild-type compared to *Pparb/d*$^{-/-}$ mice. This is associated with higher b-Catenin protein levels, which correlate with a higher b-Catenin transcriptional activity, indicated by its phosphorylation on Ser 675. Slug and N-Cadherin proteins are also found at higher levels in grade II SCC of wild-type mice. Moreover, IHC directed against N-Cadherin shows that grade II SCC of wild-type mice expressed more N-Cadherin. These new results are presented on Page 11.

Figure 7: Why is the data shown for MMP19 rather than TGFB1 in Figure 7B.

Reply

As shown in the first version of the manuscript, the correlation between *PPARB/D* and *MMP19* mRNA expression was highly significant in human SCC samples, and it was proposed by others that *MMP19* plays a role in human SCC (Sadowski et al, 2005). Collectively, these results may suggest a role of PPARb/d in EMT initiation. In the present version of the manuscript, we also show a highly significant correlation in the expression of PPARB/D and TGFB1 in human SCC samples (new Fig 7B and page 11).

Moreover, a bioinformatics analysis indicated a positive interaction between PPARB/D, SRC, and TGFB1 in human skin SCC (n = 42 samples). These new results are shown in the new Fig 7D, E and Fig 7F for the meta-analysis comprising all human carcinoma samples tested.

Referee #2 (Remarks):

This is an interesting paper, reporting on transcriptional control of the c-Src gene by the nuclear receptor PPARß/δ in the context of skin squamous cell cancer development. The findings are interesting and overall convincing. There are a few issues that need to be addressed:

1) The major conclusion that c-Src is direct gene target of PPARß/δ in keratinocytes needs to better supported. Chromatin Immunoprecitiation assays demonstrating the binding of endogenous PPARß/δ to the c-Src gene are necessary, as well as the demonstration that c-Src gene transcription occurs in keratinocytes upon PPARß/δ ligand activation in the absence of protein synthesis.

Reply

To address the reviewer's question, we have first performed experiments using cycloheximide, an inhibitor of protein synthesis, in the HaCaT keratinocytes. Our previous experiments have shown that ANGPTL4 and SRC were induced after 4 h of GW501516 treatment in HaCaT cells (unpublished results). To test whether PPARb/d directly acts at the transcriptional level or *via de novo* protein synthesis, cells were co-treated for 4 h with cycloheximidine and with (or without) GW501516. The results shown in the new Supporting Information Fig S5D demonstrate that the GW501516-dependent stimulation of *ANGPTL4* and *SRC* was not affected by cycloheximide, suggesting that PPARb/d directly regulates their transcription by a mechanism independent of *de novo* protein synthesis.

Second, as suggested by the reviewer, we have performed ChIP experiments by first performing ChIP with an antibody against PPARb/d and then a re-ChiP with an antibody against p300, a co-activator of PPARb/d, to reveal the transcriptionally active form of PPARb/d. These experiments were conducted in mouse keratinocytes in which *Pparb/d* was down-regulated by transfection with a siRNA construct against mouse *Pparβ/δ* (knockdown cells) or with scrambled siRNA (wild-type cells). The results are shown in new Figure 3D and E for the "ChIP PPARb/d" and "ChIP PPARb/d/re-ChIP p300", respectively.

The "ChIP PPARb/d" results show that PPARb/d binds PPREs 3, 4, and 5 among the five PPREs identified *in silico* and tested in the trans-activation assay, demonstrating that these PPREs are functional in keratinocytes. There was no signal for PPREs 1 and 2. Moreover, a stronger ChIP signal was obtained from wild-type cells when compared to si-PPARb/d cells, indicating that the knockdown of PPARb/d was successful. However, the weak signal in si-PPARb/d cells suggests that the knockdown was not 100%, which was confirmed by the qPCR test presented in the new Supporting Information Fig S5E, showing a 70% *Pparb/d* knockdown. Importantly, the negative control and pre-immune (p.i.) did not generate any PCR product, confirming that the ChIP directed against PPARb/d was specific.

More interestingly, ChIP with PPARb/d followed by re-ChIP with the p300 antibody showed a clear signal on PPREs 3, 4, and 5 only in GW501516-treated WT cells when compared to vehicle-treated WT cells. This indicates that GW501516 activation of PPARb/d recruits p300 to these PPREs, suggesting a mechanism for PPARb/d regulation of the *Src* gene expression upon ligand activation. Consistently, with ChIP directed against PPARb/d, no PCR product was detected for PPREs 1 and 2. We observed a weak signal with GW501516-treated si-PPARb/d cells when compared to vehicle-treated si-PPARb/d cells, which is likely due to the incomplete knockdown (70%) of PPARb/d (see above). The negative control and pre-immune (p.i.) did not generate any signal confirming that the re-ChIP was specific for p300.

Collectively, these new data demonstrate that the stimulation of *Src* by PPARb/d does not require *de novo* protein synthesis and strongly suggest that *Src* is a direct PPARb/d target gene. These finding are presented on Page 8, §2.

2) As the authors point out in the discussion, little is known on transcriptional control of the c-Src gene. Based on the data that they provide, a possibility to be considered is that at least some of the effects that they observe on c-Src expression are mediated by activation of TGF-ß/Smad signaling. In addition, the various EMT markers that were examined are also well known TGF-ß/Smad targets. These possibilities that need to consider in the interpretation /discussion of the data.

Reply

These remarks are relevant, and we have modified the text in the revised version as follows:

- Results section, Page, 10§1: "*PPARß/δ coordinates a pro-tumoral gene program in advanced actinic keratosis.* Skin tumor growth is associated with increased proliferation and migratory potential of keratinocytes, and in this process, Src promotes epithelial cell dedifferentiation during the epithelial-to-mesenchymal transition (EMT). The EMT might also be regulated by other direct or indirect PPARß/δ-modulated signaling pathways such as the Tgfβ1/SMAD pathway, which could act on *Src* expression (Glick, 2012; Han et al, 2005; Hoot et al, 2008; Martins et al, 2009; Nakamura & Tokura, 2011; Newkirk et al, 2007)."

- Discussion section, Page 13, §1: "Importantly, this pathway and its cellular effects may also be modulated by other PPARß/δ-dependent or -independent signaling pathways, such as Tgfβ1/SMAD."

3) The specificity of effects on c-Src gene expression, versus other family members (Fyn, Yes) is very interesting and should be shown. Is the transcription regulatory region of Fyn and Yes devoid of PPARß/δ binding sites as determined by bioinformatics analysis?

Reply

To answer the reviewer's question, we first tried *in silico* analysis to identify PPREs in the promoter region of the *Fyn* and *Yes* genes using Nubiscan software (http://www.nubiscan.unibas.ch/). Because PPREs have also been identified in the transcribed region, we additionally tested the region downstream of the transcription start site (TSS). The *Fyn* promoter region has been analyzed in chronic myelogenous leukemia cell lines (Gao et al, 2009). Our search for response elements up to 4 kb upstream from the TSS identified by Gao *et al* and up to 21 kb downstream of it, which comprises the 2 first introns, did not reveal any PPRE. For *Yes*, we analyzed a genomic region of 5 kb upstream of the TSS (Matsuzawa et al, 1991) and 33.8 kb downstream of it. Interestingly, we found three putative PPREs at positions -3701 bp (AGTCCTgAGTTCA), -1345 bp (AGGCAAcAGCTCA), and +4876 bp (AGGTGAgAGGACA).

To check whether *Fyn* and *Yes* gene expression could be directly or indirectly regulated by PPARb/d in UV-induced skin cancer, we performed qPCR experiments on RNA extracted from the epidermis and dermis compartments of acutely irradiated dorsal skin samples from *Pparb/d*$^{+/+}$ and *Pparb/d*$^{-/-}$ mice. Data shown in the new Figure 2A demonstrate that *Fyn* is mainly expressed in the dermis compartment and that its expression is slightly increased upon UV exposure but in a PPARb/d-independent manner. *Yes* is expressed at similar levels in dermis and epidermis compartments in *Pparb/d*$^{+/+}$ mice in the absence of UV exposure, while its expression is lower in dermis and epidermis of *Pparb/d*$^{-/-}$ mice, suggesting a positive function of PPARb/d in *Yes* gene expression in the "non-challenged" condition. Surprisingly, whereas *Yes* expression is significantly increased in epidermis compartment under acute UV exposure in wild-type mice, its expression is increasing in dermis and epidermis of *Pparb/d*$^{-/-}$ mice but at similar levels compared to *Pparb/d*$^{+/+}$ mice.

Altogether, these results suggest that whether PPARb/d seems to regulate *Yes* expression in skin in the unchallenged condition, *Fyn* and *Yes* expression is not regulated by PPARb/d upon UV exposure, in contrast to *Src*. These findings allowed us to focus on the PPARb/d-dependent regulation of *Src* gene expression in our model of UV-induced skin carcinoma. The text (Page 7, §2) has been modified, taking into account these new results.

4) For the expression data shown in Fig. 1A, only mice at 31 weeks of UV exposure were analyzed. It would be desirable to include in the analysis early times, especially considering that 100% of mice develop tumors by 22 weeks.

© EMBO

Reply

As mentioned by the reviewer, although tumourigenesis is enhanced in the wild-type mice compared to the null mice, a catch-up effect is observed in *Pparb/d*$^{-/-}$ mice after 22 weeks of chronic UV exposure, a point also noted by Reviewer 1 who asks us to identify signaling pathways that could explain it. As noticed by the reviewer, only mice at 31 weeks of UV exposure were mainly analyzed in our study and show in Fig 1A. Nevertheless, data from Supporting Information Fig S1, which show *Ppars* expression at different time point of chronic UV exposure, demonstrate that at least PPARb/d gene expression is progressively increasing with week of chronic UV exposure. To know whether the identified dysregulated signaling pathway identify after 31 weeks of chronic UV is also affected at early time points and to determine whether other signaling pathways are also dysregulated, leading to explain the catch-up effect observed in *Pparb/d*$^{-/-}$, the following approach may be suggested. The first step would be RNA profiling in both wild-type and *Pparb/d*$^{-/-}$ mice at weeks 0, 12, and 22. Changes in the expression profiles should be studied together with gene mutations occurring during UV exposure. Such an approach might help to explain the catch-up effect but clearly represents a study on its own and is beyond the scope of the current work.

5) Representative histological and immunohistochemical images of tumors are necessary, together with an adequate assessment (grading) of tumor cell differentiation. It is also important to determine how many of the UV-induced spindle cell tumors are positive for keratin expression, as keratin-negative tumors could also be mesenchymally-derived (sarcomas).

Reply

As described in the Material and Methods section, tumors collected from wild-type and *Pparb/d*$^{-/-}$ mice were graded histologically in a blind manner by a pathologist according to the Broders' classification, which is based on the degree of differentiation and keratinization of tumor cells (Broders, 1921). This classification is as follows: SCC Grade I: 75% keratinocytes are well differentiated; SCC Grade II: >50% keratinocytes are well differentiated; SCC Grade III: >25% keratinocytes are well differentiated; and SCC Grade IV: <25% keratinocytes are well differentiated. Based on this analysis, we observed that *Pparb/d*$^{+/+}$ animals developed more advanced SCCs, as is now illustrated in the new Figure 6E of the revised version of the manuscript. This panel represents the tumor type distribution for each genotype. The grading of actinic keratoses, considered to be pre-cancerous tumors, was also established in a blind manner by the pathologist, according to Rowert-Huber *et al* (Rowert-Huber et al, 2007), as grade I, II, or III based on the extent of cytological atypia of epidermal keratinocytes (mild, grade I; moderate, grade II; and severe, grade III) and involvement of adnexal structures. The result of this grading distribution in wild-type and *Pparb/d*$^{-/-}$ mice is also presented in Fig 6E. Wild-type mice presented a higher grading of actinic keratosis.

In addition, representative hematoxylin/eosin staining and IHC directed against K10 and K14, which are markers of suprabasal differentiated and basal proliferating keratinocytes, respectively, were performed on representative actinic keratosis with moderate and severe atypia (grades II and III, respectively) and grades I, II, and III skin SCCs from wild-type mice (Supporting Information Fig S12).

Importantly, according to the histopathological analysis of the PPARβ/δ wt and null tumors and as detailed above, the PPARβ/δ wt animals developed more advanced SCCs (Fig 6E). Because of the conditions imposed by the Veterinary Office of the "Canton de Vaud", in accordance with the Federal Swiss Veterinary Office Guidelines, our UV-exposed animals had to be sacrificed as soon as one of their tumors reached 9 mm in diameter. In these conditions, it was not possible to obtain metastatic SCCs, although very rare spindle cells were identified in the most advanced SCCs obtained in wild-type mice (histological analysis and dermis infiltration of group of cells positive for Vimentin/Keratin K14 co-staining). Because these samples were not representative of all wild-type mice tested, but quite marginal, we have preferred not to show these data. Studies by other laboratories under a less constraining regulation than ours to obtain metastatic SCCs would have required longer or higher doses of UV exposure as reported in several publications (de Gruijl et al, 1993; de Gruijl & Forbes, 1995; Forbes et al, 1978; Perez et al, 2012; Thomas-Ahner et al, 2007). Again, we could not perform such studies because of the prohibition by the Veterinary Office of the "Canton de Vaud".

6) The existence of a positive feedback loop between c-Src and PPARß/δ expression and activity is very interesting and supporting data should be shown as an integral part of the paper, rather than as supplemental material.

Reply

As the reviewer has requested, the data showing that GSK0660 induces a decrease in *Pparb/d* and *Src* gene expression, previously shown in Supporting Information Fig S8 and Fig 5, is now integrated into a new Fig 5F. A sentence has been added on Page 9§2 about this possible positive feedback loop between *Pparb/d* and *Src* expression and activity.

7) In the analysis of various tumor types shown in Fig. 7C, skin or oral SCC data would have to be included.

Reply

We fully agree with the referee that extending the analysis presented in Fig 7C to include squamous tumors, both oral and skin, would definitely improve the quality and consistency of the manuscript. To address this point, the following three datasets (2 OSCC and 1 SSC) were identified within the NCBI Gene Expression Omnibus (GEO) (http://www.ncbi.nlm.nih.gov/geo/) database:

- GSE30784 (Chen et al, 2008): Oral squamous cell carcinoma (OSCC); N = 229 samples; Platform: Affymetrix Human Genome U133 Plus 2.0 Array
- GSE41613 (Lohavanichbutr et al, 2013): Oral Squamous Cell Carcinoma (OSCC); N = 97 samples; Platform: Affymetrix Human Genome U133 Plus 2.0 Array
- GSE32628 (Hameetman et al, 2013): Cutaneous squamous cell carcinomas (SSC); N = 42 samples; Platform: Illumina human-6 v2.0 expression beadchip

These gene expression datasets are characterized by multiple probe sets mapping to a single gene. Therefore, a selection procedure is required to identify the probe sets that will be included in the linear model. In Fig 7C of the previous version of the manuscript, the probe set selection process was based on highest variance. However, this methodological approach failed when analyzing the GSE30784 dataset. An alternative approach was used that took advantage of the fact that both GSE30784 and GSE41613 were two independent OSCC studies that used the same technological platform.

The GSE30784 was used to select probe set combinations (among those mapping to SRC, TGFB1, and PPARB/D) that maximizes the value of the interaction term coefficient. The selected probe sets were then directly used for the analysis of the GSE41613 datasets. Only the result for the GSE41613 datasets is reported in the manuscript, to avoid any optimization bias.

In regards to the skin SSC, as only a single dataset was identified in the GEO database, probe set selection was performed using highest variance.

Analysis of the OSCC dataset (GSE41613) showed a significant interaction coefficient term 0.009 [0.003–0.014] 95%CI; $p = 0.004$ (Fig 7F; Supporting Information Fig 14E). The actual interaction can be visualized by investigating the linear relationship between *SRC* and *TGFB1* in subjects having either high or low *PPARB/D* expression. In this analysis, high or low expression was categorized using the median value of *PPARB/D* within the studied dataset. Absence of linear dependency was observed in samples having a *PPARB/D* expression below its median value (regression coefficient of -0.002, $p = 0.63$, correlation coefficient -0.071; Supporting Information Fig 14H). In contrast, a significant linear relationship was observed between *SRC* and *TGFB1* mRNA abundance in samples having high *PPARB/D* mRNA expression above the median value (regression coefficient = 0.011, $p = 0.033$ and correlation coefficient: 0.31; Supporting Information Fig 14I). In summary, the OSCC dataset supports previous claims of a significant interaction between *PPARB/D* and *TGFB1* to explain *SRC* mRNA expression.

In regard to SSC, analysis of the dataset (GSE32628) showed a marginally significant interaction coefficient (regression coefficient = 6.76; $p = 0.061$). This value and its 95%CI are presented in Fig 7E. As for the OSCC dataset, the nature of the interaction was visualized in high and low *PPARB/D* expressers. Consistent with previous observation, an absence of linear dependency was seen in samples having a PPARB/D mRNA abundance below the median value (regression coefficient = -0.23, $p = 0.75$, correlation = -0.052; Fig 7D), while significant linear relations were observed in samples with *PPARB/D* mRNA equal to or above the median value (regression coefficient = 4.78, $p = 0.0336$, and correlation = 0.46; Fig 7E).

In our view, the SCC analysis also supports the previous observation. Although the significance of the linear model was marginal ($p = 0.06$), the interaction of *PPARB/D* was consistent with previous findings.

Because the SSC regression interaction coefficient differs in magnitude relative to the other datasets (it was the only dataset derived using the Illumina bead array), results are best presented within a table rather than in the form of a Forest plot. A forest plot is provided in Supporting Information Fig 14E presenting all datasets except the SSC datasets.

We also would like to mention that we have taken out the pancreatic cancer dataset because it represented only 14 patients; this exclusion had no effect on the conclusions of the study.

In light of these results, both the methods and results sections have been modified accordingly. Of note, an error was detected and corrected in the methods section in which the tested model was SRC ~ TGFB1 + PPARB/D + PPARB/D: TGFB1 and not TGFB1 ~ SRC + PPARB/D + PPARB/D: TGFB1.

Referee #3 (Comments on Novelty/Model System):
The involvement of PPARdelta in oncogenic activities and the role of the receptor in EGFR-induced cancer cell proliferation were previously documented. The authors point at a controversy in the literature but the cited references suggest that while several groups reported that PPARdelta exerts pro-oncogenic activities, only one group suggested the opposite.

Reply

It is correct that several groups have reported pro-oncogenic activities of PPARβ/δ, but although much less numerous, more than one group also has shown some anti-tumorigenic activity of PPARβ/δ, for instance in colorectal cancer (see Discussion section).

Referee #3 (Remarks):
Work described in this manuscript investigated the involvement of the nuclear receptor PPARd in UV-induced skin cancer. The data indicate that: 1) UV irradiation induced the expression of PPARd and two known PPARd target genes in mouse skin; 2) that UV-induced skin tumor formation was delayed in PPARd-null vs. WT mice; 3) activation of PPARd in keratinocytes resulted in increased expression of Src, leading to activation of EGFR and ERK1/2, 4) skins of UV-irradiated PPARd-null mice displayed lower expression of markers of proliferation and invasion, and 5) a correlation between the expression levels of PPARd and Src was found in various human tumors. Overall, the data support previous observations from this and other groups indicating that PPARd exerts pro-proliferative and oncogenic activities in some cells.

Comments:
- The involvement of PPARd in oncogenic activities was previously reported. The main novel finding of the present work is the identification of Src as a direct target gene for this receptor. In addition to the reporter transactivation assays, a more comprehensive analysis should be carried out to confirm this conclusion. Does PPARd indeed target Src directly, i.e. without the need of de novo protein synthesis? Is the receptor associated with the Src promoter in cells?

Reply

This important and relevant question, also asked by Reviewer 2, is addressed in the revised paper.

To address the reviewers' question, we have first performed experiments using cycloheximide, an inhibitor of protein synthesis, in the HaCaT keratinocytes. Our previous experiments have shown that ANGPTL4 and SRC were induced after 4 h of GW501516 treatment in HaCaT cells (unpublished results). To test whether PPARb/d directly acts at the transcriptional level or *via de novo* protein synthesis, cells were co-treated for 4 h with cycloheximidine and with (or without) GW501516. The results shown in the new Supporting Information Fig S5D demonstrate that the GW501516-dependent stimulation of *ANGPTL4* and *SRC* was not affected by cycloheximide, suggesting that PPARb/d directly regulates their transcription by a mechanism independent of *de novo* protein synthesis.

Second, as suggested by the reviewer, we have performed ChIP experiments by first performing ChIP with an antibody against PPARb/d and then a re-ChiP with an antibody against p300, a co-activator of PPARb/d, to reveal the transcriptionally active form of PPARb/d. These experiments were conducted in mouse keratinocytes in which *Pparb/d* was down-regulated by transfection with a siRNA construct against mouse *Pparβ/δ* (knockdown cells) or with scrambled siRNA (wild-type cells). The results are shown in new Figure 3D and E for the "ChIP PPARb/d" and "ChIP PPARb/d/re-ChIP p300", respectively.

The "ChIP PPARb/d" results show that PPARb/d binds PPREs 3, 4, and 5 among the five PPREs identified *in silico* and tested in the trans-activation assay, demonstrating that these PPREs are functional in keratinocytes. There was no signal for PPREs 1 and 2. Moreover, a stronger ChIP signal was obtained from wild-type cells when compared to si-PPARb/d cells, indicating that the knockdown of PPARb/d was successful. However, the weak signal in si-PPARb/d cells suggests that the knockdown was not 100%, which was confirmed by the qPCR test presented in the new Supporting Information Fig S5E, showing a 70% *Pparb/d* knockdown. Importantly, the negative control and pre-immune (p.i.) did not generate any PCR product, confirming that the ChIP directed against PPARb/d was specific.

More interestingly, ChIP with PPARb/d followed by re-ChIP with the p300 antibody showed a clear signal on PPREs 3, 4, and 5 only in GW501516-treated WT cells when compared to vehicle-treated WT cells. This indicates that GW501516 activation of PPARb/d recruits p300 to these PPREs, suggesting a mechanism for PPARb/d regulation of the *Src* gene expression upon ligand activation. Consistently, with ChIP directed against PPARb/d, no PCR product was detected for PPREs 1 and 2. We observed a weak signal with GW501516-treated si-PPARb/d cells when compared to vehicle-treated si-PPARb/d cells, which is likely due to the incomplete knockdown (70%) of PPARb/d (see above). The negative control and pre-immune (p.i.) did not generate any signal confirming that the re-ChIP was specific for p300.

Collectively, these new data demonstrate that the stimulation of *Src* by PPARb/d does not require *de novo* protein synthesis and strongly suggest that *Src* is a direct PPARb/d target gene. These finding are presented on Page 8, §2.

- The involvement of PPARd in induction carcinoma cell growth by EGFR was previously reported (JBC 2010 285:19106). These findings should be discussed.

Reply

In the first version of the manuscript, this paper from Noa Noy's laboratory was included and commented on in the Discussion section especially for its relevance on PPARb/d activity being enhanced by FABP5 in breast cancer. This finding introduces a new level of complexity in the mechanisms involving PPARb/d in tumorigenesis (Kannan-Thulasiraman et al, 2010). The above-cited study demonstrates that Heregulin-b1–induced activation of ErbB2, ErbB3, and ErbB4, which belong to the EGFR superfamily, leads to the induction of *FABP5* expression, which in turn enhances PPARb/d transcriptional activity in the MCF-7 cell line. In the present study, we demonstrate that PPARb/d acts upstream from EGFR (ErbB1) by up-regulating a kinase involved in its full activation. To address the reviewer's query in a satisfactory manner, the JBC 2010 paper is discussed as follows on Page 14, line 12 in the present revised version of the paper: "In accordance with our observations, ErbB2 activation, in concert with ErbB3 and ErbB4, leads to the expression of *FABP5*, an intracellular lipid binding protein, which plays a crucial role in PPARb/d transcriptional activation in the MCF7 cell line, suggesting that the EGFR superfamily could also act upstream of PPARb/d to regulate its activity in a model of breast cancer (Kannan-Thulasiraman et al, 2010)."

- Considering that ablation of PPARd somewhat delayed but did not prevent UV-induced tumor development in mice (Fig. 1B), the authors' suggestion that this receptor may comprise a target for anti-cancer compounds is a stretch.

Reply

As mentioned by the reviewer, *Pparb/d*$^{-/-}$ mice finally also develop skin tumors. Parts of the manuscript have been rewritten accordingly.

References

Biscardi JS, Maa MC, Tice DA, Cox ME, Leu TH, Parsons SJ (1999) c-Src-mediated phosphorylation of the epidermal growth factor receptor on Tyr845 and Tyr1101 is associated with modulation of receptor function. J Biol Chem 274**:** 8335-8343

Broders AC (1921) Squamous-Cell Epithelioma of the Skin: A Study of 256 Cases. Ann Surg 73**:** 141-160

© EMBO

Carter WG, Kaur P, Gil SG, Gahr PJ, Wayner EA (1990) Distinct functions for integrins alpha 3 beta 1 in focal adhesions and alpha 6 beta 4/bullous pemphigoid antigen in a new stable anchoring contact (SAC) of keratinocytes: relation to hemidesmosomes. J Cell Biol 111**:** 3141-3154

Carter WG, Ryan MC, Gahr PJ (1991) Epiligrin, a new cell adhesion ligand for integrin alpha 3 beta 1 in epithelial basement membranes. Cell 65**:** 599-610

Chen C, Mendez E, Houck J, Fan W, Lohavanichbutr P, Doody D, Yueh B, Futran ND, Upton M, Farwell DG et al (2008) Gene expression profiling identifies genes predictive of oral squamous cell carcinoma. Cancer Epidemiol Biomarkers Prev 17**:** 2152-2162

Dajee M, Lazarov M, Zhang JY, Cai T, Green CL, Russell AJ, Marinkovich MP, Tao S, Lin Q, Kubo Y et al (2003) NF-kappaB blockade and oncogenic Ras trigger invasive human epidermal neoplasia. Nature 421**:** 639-643

Darwiche N, Ryscavage A, Perez-Lorenzo R, Wright L, Bae DS, Hennings H, Yuspa SH, Glick AB (2007) Expression profile of skin papillomas with high cancer risk displays a unique genetic signature that clusters with squamous cell carcinomas and predicts risk for malignant conversion. Oncogene 26**:** 6885-6895

de Gruijl FR, Forbes PD (1995) UV-induced skin cancer in a hairless mouse model. Bioessays 17**:** 651-660

de Gruijl FR, Sterenborg HJ, Forbes PD, Davies RE, Cole C, Kelfkens G, van Weelden H, Slaper H, van der Leun JC (1993) Wavelength dependence of skin cancer induction by ultraviolet irradiation of albino hairless mice. Cancer Res 53**:** 53-60

Emlet DR, Moscatello DK, Ludlow LB, Wong AJ (1997) Subsets of epidermal growth factor receptors during activation and endocytosis. J Biol Chem 272**:** 4079-4086

Ettenberg SA, Keane MM, Nau MM, Frankel M, Wang LM, Pierce JH, Lipkowitz S (1999) cbl-b inhibits epidermal growth factor receptor signaling. Oncogene 18**:** 1855-1866

Forbes PD, Davies RE, Urbach F (1978) Experimental ultraviolet photocarcinogenesis: wavelength interactions and time-dose relationships. Natl Cancer Inst Monogr**:** 31-38

Fritsche E, Schafer C, Calles C, Bernsmann T, Bernshausen T, Wurm M, Hubenthal U, Cline JE, Hajimiragha H, Schroeder P et al (2007) Lightening up the UV response by identification of the arylhydrocarbon receptor as a cytoplasmatic target for ultraviolet B radiation. Proc Natl Acad Sci U S A 104**:** 8851-8856

Gao Y, Howard A, Ban K, Chandra J (2009) Oxidative stress promotes transcriptional up-regulation of Fyn in BCR-ABL1-expressing cells. J Biol Chem 284**:** 7114-7125

Glick AB (2012) The Role of TGFbeta Signaling in Squamous Cell Cancer: Lessons from Mouse Models. J Skin Cancer 2012**:** 249063

Hameetman L, Commandeur S, Bavinck JN, Wisgerhof HC, de Gruijl FR, Willemze R, Mullenders L, Tensen CP, Vrieling H (2013) Molecular profiling of cutaneous squamous cell carcinomas and actinic keratoses from organ transplant recipients. BMC Cancer 13**:** 58

Han G, Lu SL, Li AG, He W, Corless CL, Kulesz-Martin M, Wang XJ (2005) Distinct mechanisms of TGF-beta1-mediated epithelial-to-mesenchymal transition and metastasis during skin carcinogenesis. J Clin Invest 115**:** 1714-1723

Hoot KE, Lighthall J, Han G, Lu SL, Li A, Ju W, Kulesz-Martin M, Bottinger E, Wang XJ (2008) Keratinocyte-specific Smad2 ablation results in increased epithelial-mesenchymal transition during skin cancer formation and progression. J Clin Invest 118**:** 2722-2732

© EMBO

Kannan-Thulasiraman P, Seachrist DD, Mahabeleshwar GH, Jain MK, Noy N (2010) Fatty acid-binding protein 5 and PPARbeta/delta are critical mediators of epidermal growth factor receptor-induced carcinoma cell growth. J Biol Chem 285**:** 19106-19115

Kim HJ, Ham SA, Kim SU, Hwang JY, Kim JH, Chang KC, Yabe-Nishimura C, Seo HG (2008) Transforming growth factor-beta1 is a molecular target for the peroxisome proliferator-activated receptor delta. Circ Res 102**:** 193-200

Kim SJ, Angel P, Lafyatis R, Hattori K, Kim KY, Sporn MB, Karin M, Roberts AB (1990) Autoinduction of transforming growth factor beta 1 is mediated by the AP-1 complex. Mol Cell Biol 10**:** 1492-1497

Larcher F, Bauluz C, Diaz-Guerra M, Quintanilla M, Conti CJ, Ballestin C, Jorcano JL (1992) Aberrant expression of the simple epithelial type II keratin 8 by mouse skin carcinomas but not papillomas. Mol Carcinog 6**:** 112-121

Lee CG, Homer RJ, Zhu Z, Lanone S, Wang X, Koteliansky V, Shipley JM, Gotwals P, Noble P, Chen Q et al (2001) Interleukin-13 induces tissue fibrosis by selectively stimulating and activating transforming growth factor beta(1). J Exp Med 194**:** 809-821

Levkowitz G, Waterman H, Ettenberg SA, Katz M, Tsygankov AY, Alroy I, Lavi S, Iwai K, Reiss Y, Ciechanover A et al (1999) Ubiquitin ligase activity and tyrosine phosphorylation underlie suppression of growth factor signaling by c-Cbl/Sli-1. Mol Cell 4**:** 1029-1040

Lohavanichbutr P, Mendez E, Holsinger FC, Rue TC, Zhang Y, Houck J, Upton MP, Futran N, Schwartz SM, Wang P et al (2013) A 13-gene signature prognostic of HPV-negative OSCC: discovery and external validation. Clin Cancer Res 19**:** 1197-1203

Marinkovich MP (2007) Tumour microenvironment: laminin 332 in squamous-cell carcinoma. Nat Rev Cancer 7**:** 370-380

Martins VL, Vyas JJ, Chen M, Purdie K, Mein CA, South AP, Storey A, McGrath JA, O'Toole EA (2009) Increased invasive behaviour in cutaneous squamous cell carcinoma with loss of basement-membrane type VII collagen. J Cell Sci 122**:** 1788-1799

Matsuzawa Y, Semba K, Kawamura-Tsuzuku J, Sudo T, Ishii S, Toyoshima K, Yamamoto T (1991) Characterization of the promoter region of the c-yes proto-oncogene: the importance of the GC boxes on its promoter activity. Oncogene 6**:** 1561-1567

Nakamura M, Tokura Y (2011) Epithelial-mesenchymal transition in the skin. J Dermatol Sci 61**:** 7-13

Newkirk KM, Parent AE, Fossey SL, Choi C, Chandler HL, Rajala-Schultz PJ, Kusewitt DF (2007) Snai2 expression enhances ultraviolet radiation-induced skin carcinogenesis. Am J Pathol 171**:** 1629-1639

Nischt R, Roop DR, Mehrel T, Yuspa SH, Rentrop M, Winter H, Schweizer J (1988) Aberrant expression during two-stage mouse skin carcinogenesis of a type I 47-kDa keratin, K13, normally associated with terminal differentiation of internal stratified epithelia. Mol Carcinog 1**:** 96-108

Perez C, Parker-Thornburg J, Mikulec C, Kusewitt DF, Fischer SM, Digiovanni J, Conti CJ, Benavides F (2012) SKHIN/Sprd, a new genetically defined inbred hairless mouse strain for UV-induced skin carcinogenesis studies. Exp Dermatol 21**:** 217-220

Pullar CE, Baier BS, Kariya Y, Russell AJ, Horst BA, Marinkovich MP, Isseroff RR (2006) beta4 integrin and epidermal growth factor coordinately regulate electric field-mediated directional migration via Rac1. Mol Biol Cell 17**:** 4925-4935

© EMBO

Rojas M, Yao S, Lin YZ (1996) Controlling epidermal growth factor (EGF)-stimulated Ras activation in intact cells by a cell-permeable peptide mimicking phosphorylated EGF receptor. J Biol Chem 271**:** 27456-27461

Rowert-Huber J, Patel MJ, Forschner T, Ulrich C, Eberle J, Kerl H, Sterry W, Stockfleth E (2007) Actinic keratosis is an early in situ squamous cell carcinoma: a proposal for reclassification. Br J Dermatol 156 Suppl 3**:** 8-12

Russell AJ, Fincher EF, Millman L, Smith R, Vela V, Waterman EA, Dey CN, Guide S, Weaver VM, Marinkovich MP (2003) Alpha 6 beta 4 integrin regulates keratinocyte chemotaxis through differential GTPase activation and antagonism of alpha 3 beta 1 integrin. J Cell Sci 116**:** 3543-3556

Sadowski T, Dietrich S, Koschinsky F, Ludwig A, Proksch E, Titz B, Sedlacek R (2005) Matrix metalloproteinase 19 processes the laminin 5 gamma 2 chain and induces epithelial cell migration. Cell Mol Life Sci 62**:** 870-880

Savoia P, Cremona O, Trusolino L, Pepino E, Marchisio PC (1994) Integrins and basement membrane proteins in skin carcinomas. Pathol Res Pract 190**:** 950-954

Sutter C, Strickland PT, Mukhtar H, Agarwal R, Winter H, Schweizer J (1993) ras gene activation and aberrant expression of keratin K13 in ultraviolet B radiation-induced epidermal neoplasias of mouse skin. Mol Carcinog 8**:** 13-19

Tennenbaum T, Belanger AJ, Quaranta V, Yuspa SH (1996) Differential regulation of integrins and extracellular matrix binding in epidermal differentiation and squamous tumor progression. J Investig Dermatol Symp Proc 1**:** 157-161

Tennenbaum T, Weiner AK, Belanger AJ, Glick AB, Hennings H, Yuspa SH (1993) The suprabasal expression of alpha 6 beta 4 integrin is associated with a high risk for malignant progression in mouse skin carcinogenesis. Cancer Res 53**:** 4803-4810

Tennenbaum T, Yuspa SH, Grover A, Castronovo V, Sobel ME, Yamada Y, De Luca LM (1992) Extracellular matrix receptors and mouse skin carcinogenesis: altered expression linked to appearance of early markers of tumor progression. Cancer Res 52**:** 2966-2976

Thomas-Ahner JM, Wulff BC, Tober KL, Kusewitt DF, Riggenbach JA, Oberyszyn TM (2007) Gender differences in UVB-induced skin carcinogenesis, inflammation, and DNA damage. Cancer Res 67**:** 3468-3474

Tsuruta D, Kobayashi H, Imanishi H, Sugawara K, Ishii M, Jones JC (2008) Laminin-332-integrin interaction: a target for cancer therapy? Curr Med Chem 15**:** 1968-1975

Warshamana GS, Corti M, Brody AR (2001) TNF-alpha, PDGF, and TGF-beta(1) expression by primary mouse bronchiolar-alveolar epithelial and mesenchymal cells: tnf-alpha induces TGF-beta(1). Exp Mol Pathol 71**:** 13-33

Xu Y, Shao Y, Voorhees JJ, Fisher GJ (2006) Oxidative inhibition of receptor-type protein-tyrosine phosphatase kappa by ultraviolet irradiation activates epidermal growth factor receptor in human keratinocytes. J Biol Chem 281**:** 27389-27397

Zwick E, Hackel PO, Prenzel N, Ullrich A (1999) The EGF receptor as central transducer of heterologous signalling systems. Trends Pharmacol Sci 20**:** 408-412

2nd Editorial Decision 12 September 2013

Thank you for the submission of your revised manuscript to EMBO Molecular Medicine.

We have now received the enclosed reports from the Reviewers that were asked to re-assess it. As you will see the reviewers are now supportive and I am prepared to accept your manuscript for publication in EMBO Molecular Medicine. Before I can formally accept your manuscript for publication, however, there remain a few pending issues that require your action:

1) The current resolution of Figure 3, panels D and E remains poor (you can verify this easily by zooming in on the image) and should be improved.

2) As per our Author Guidelines, the description of all reported data that includes statistical testing must state the name of the statistical test used to generate error bars and P values, the number (n) of independent experiments underlying each data point (not replicate measures of one sample), and the actual P value for each test (not merely 'significant' or 'P < 0.05').

Please submit your revised manuscript within two weeks. The earlier you do so, the sooner we will be able to accept the manuscript!

I look forward to seeing a revised form of your manuscript as soon as possible.

***** Reviewer's comments *****

Referee #1 (Remarks):

I am happy with the authors rebuttal and additional work.

Referee #2 (Remarks):

This is an interesting study and the authors have addressed all my previous concerns

Referee #3 (Remarks):

The revised manuscript appropriately addressed the previous criticism and recommendations and the paper is improved. There are no additional concerns.

2nd Revision - authors' response 25 September 2013

The requested modifications have be now included in the ms and figures as the following:
- Figure3 was replaced by a new one with a higher resolution for panels D and E;
- The name of the statistical test used to generate error bars, actual P values, and the number of independent experiments performed are indicated in each Figure in figure legend. The old versions of the manuscript file and supporting information were therefore replaced.
Thank you for your interest in our study.
