# Supplementary figures and images for: Src is activated by the nuclear receptor peroxisome proliferator-activated receptor β/δ in ultraviolet radiation-induced skin cancer
Source: EMBO Mol Med. 2013 Nov 6;6(1):80–98. doi: 10.1002/emmm.201302666 (PMC3936491; doi:10.1002/emmm.201302666)

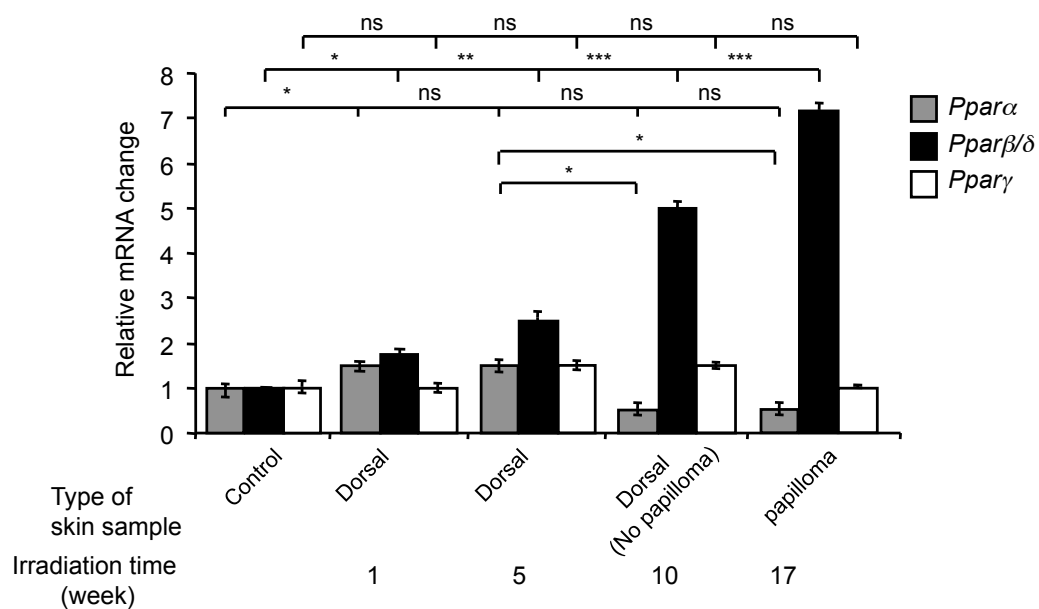

Figure S1

Supplement: Supplementary file 2 [file emmm0006-0080-sd2.pdf]

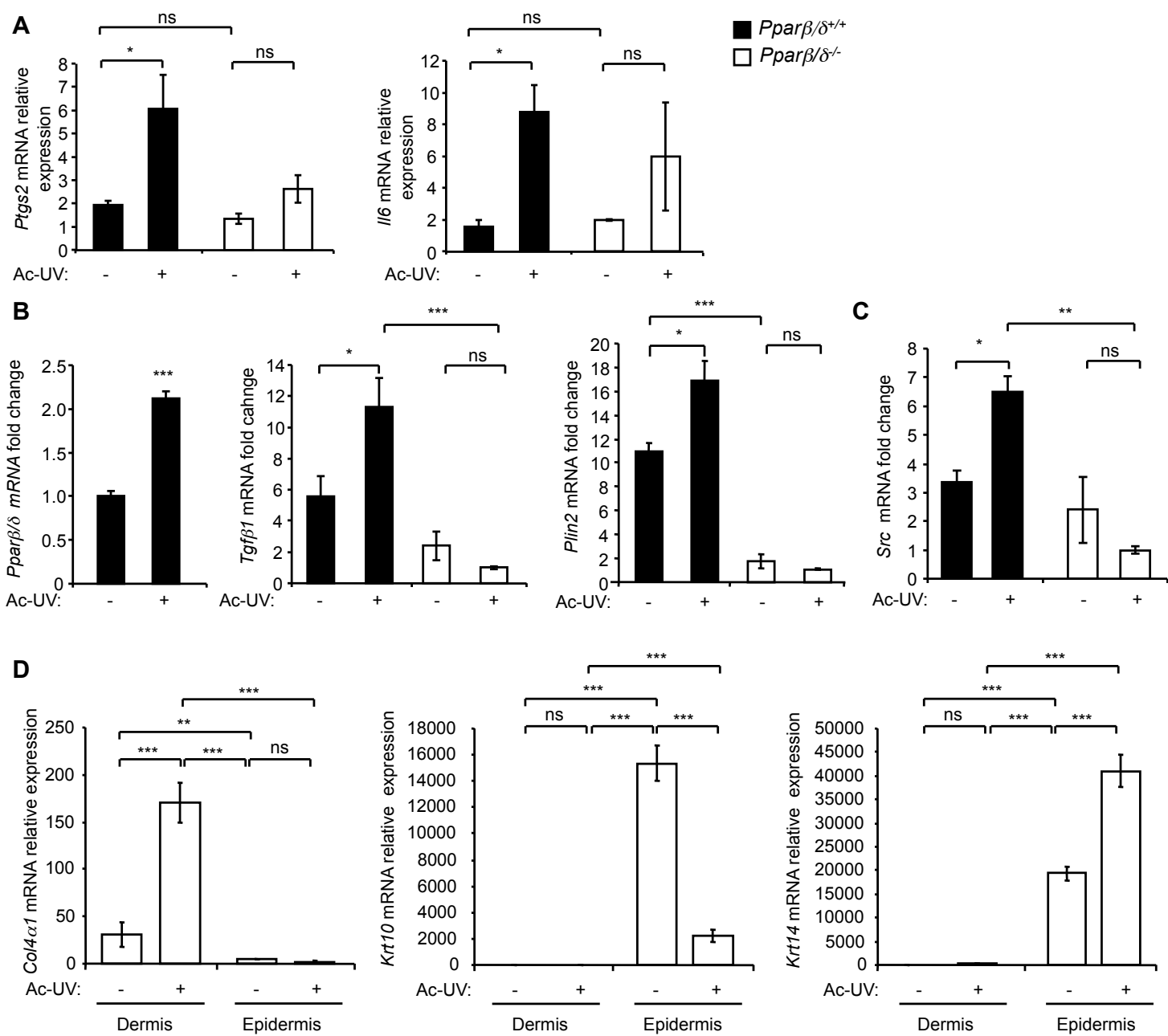

Figure S2

Supplement: Supplementary file 3 [file emmm0006-0080-sd3.pdf]

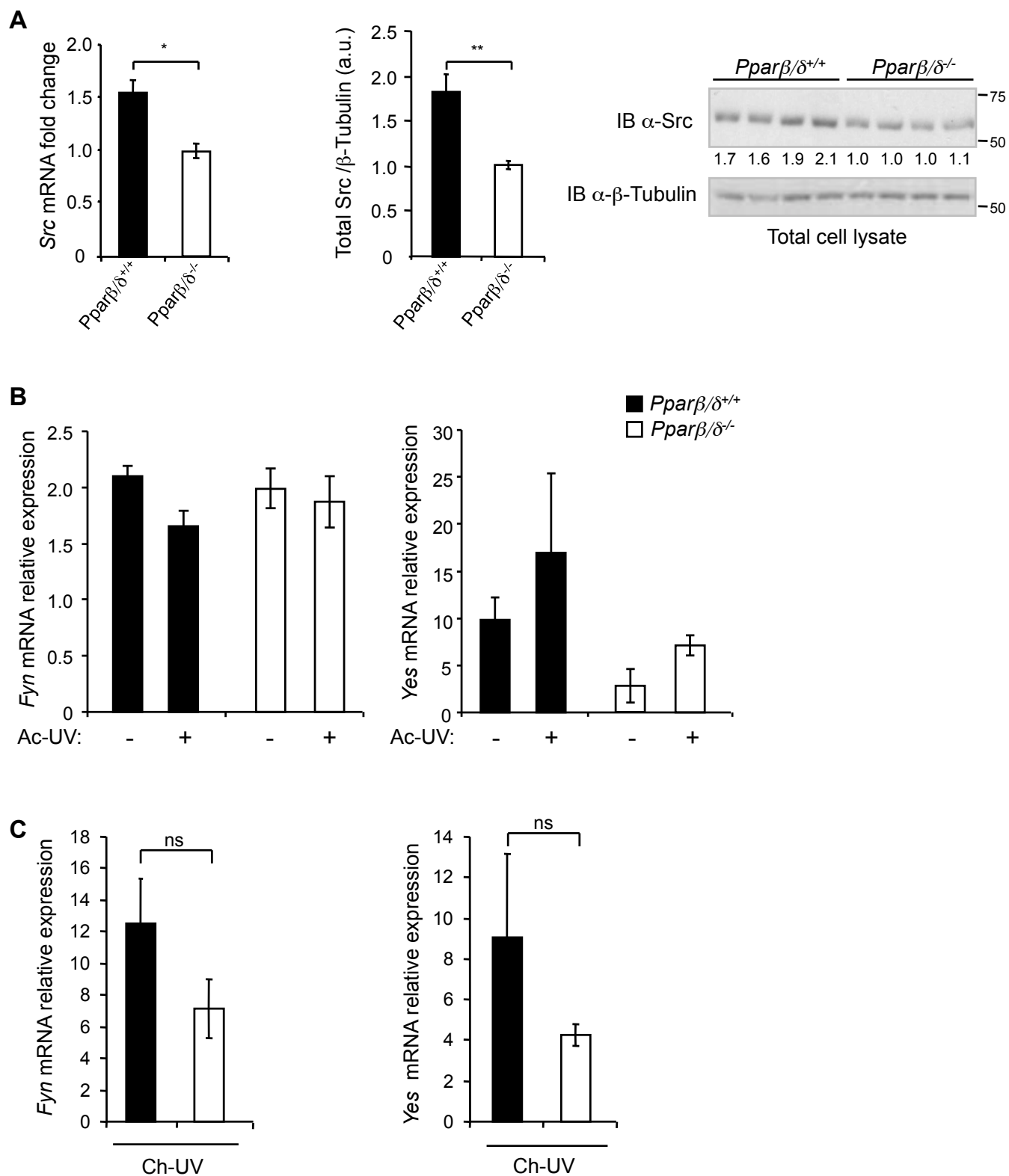

Figure S3

Supplement: Supplementary file 4 [file emmm0006-0080-sd4.pdf]

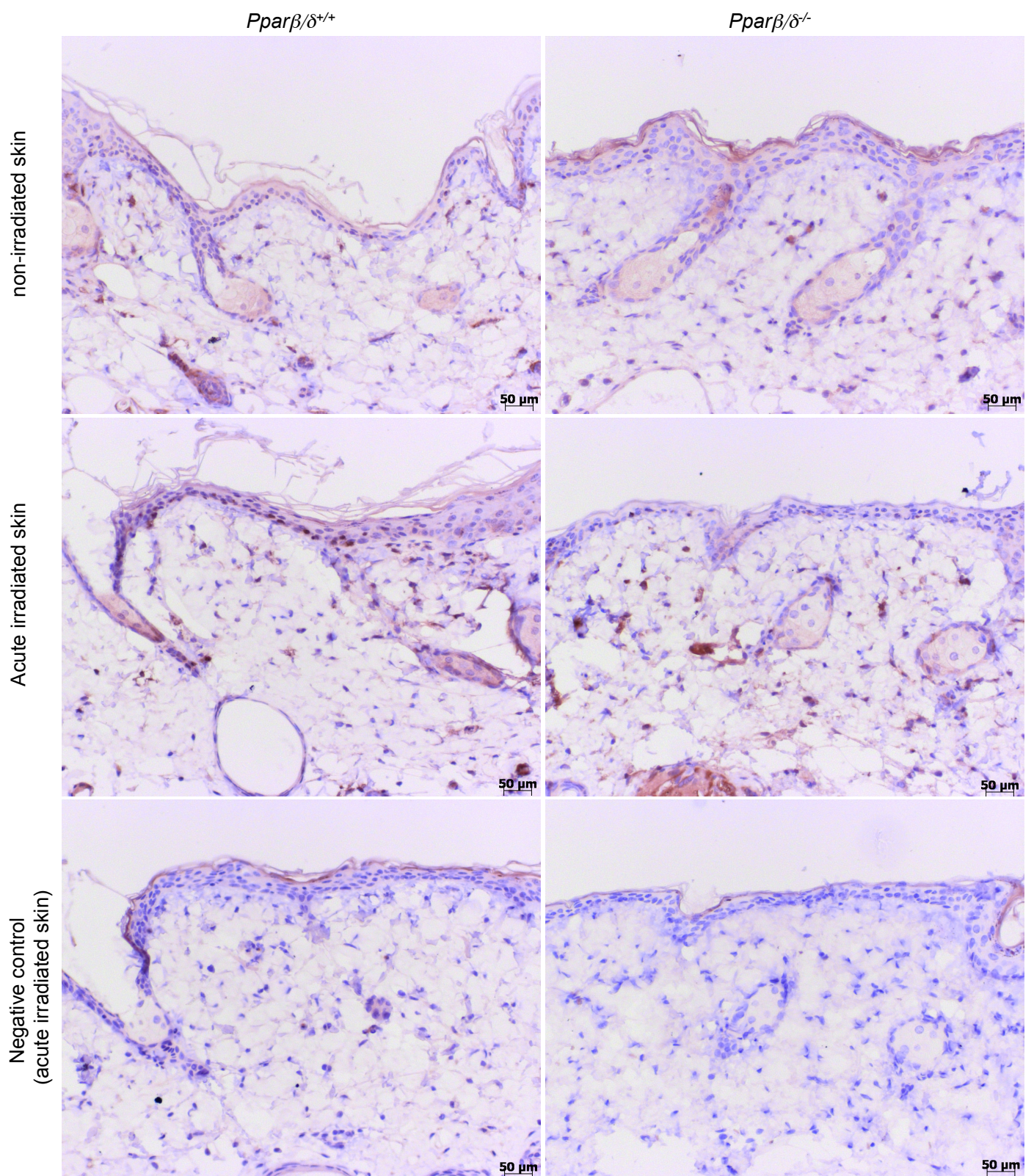

Figure S4

Supplement: Supplementary file 5 [file emmm0006-0080-sd5.pdf]

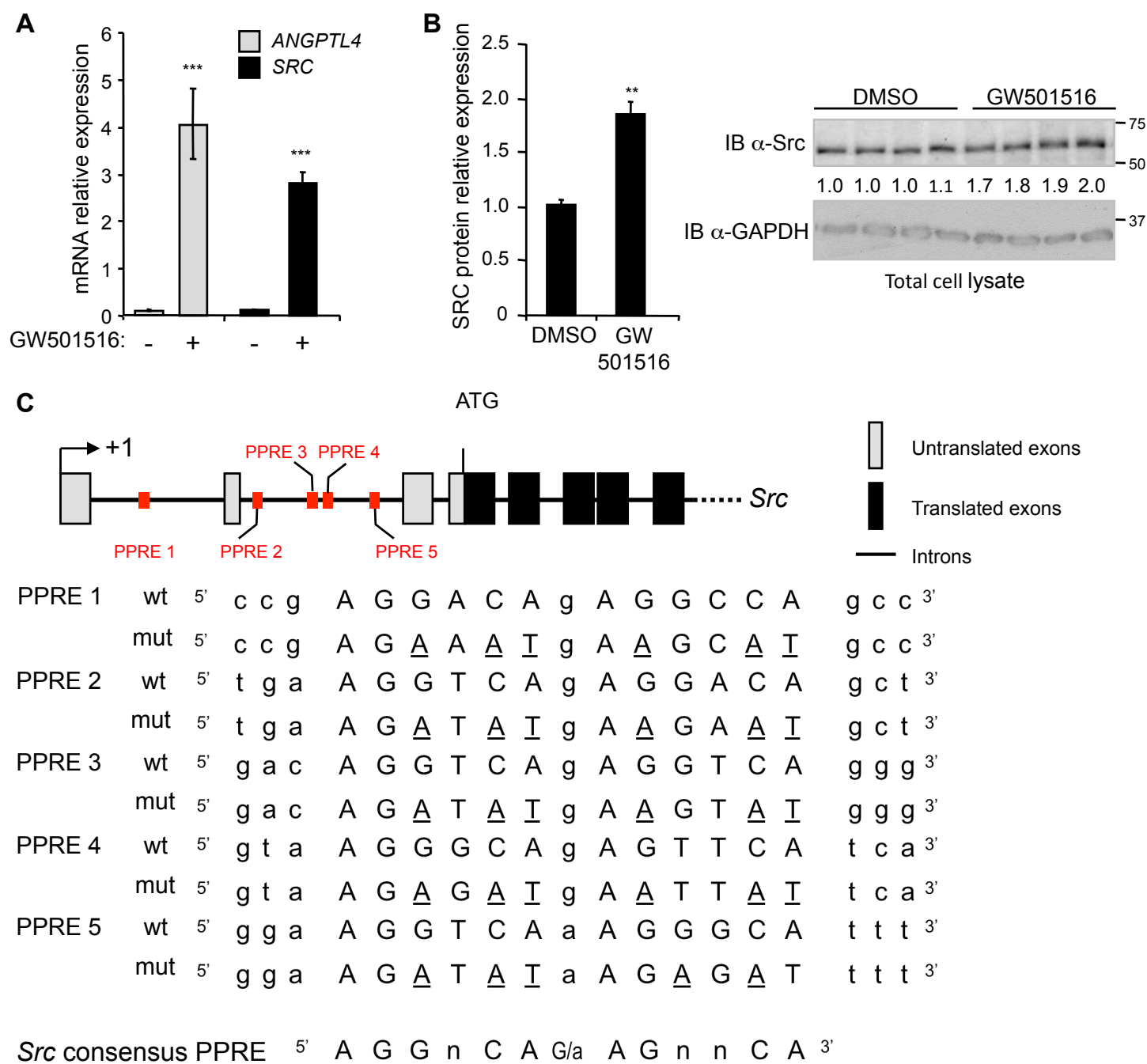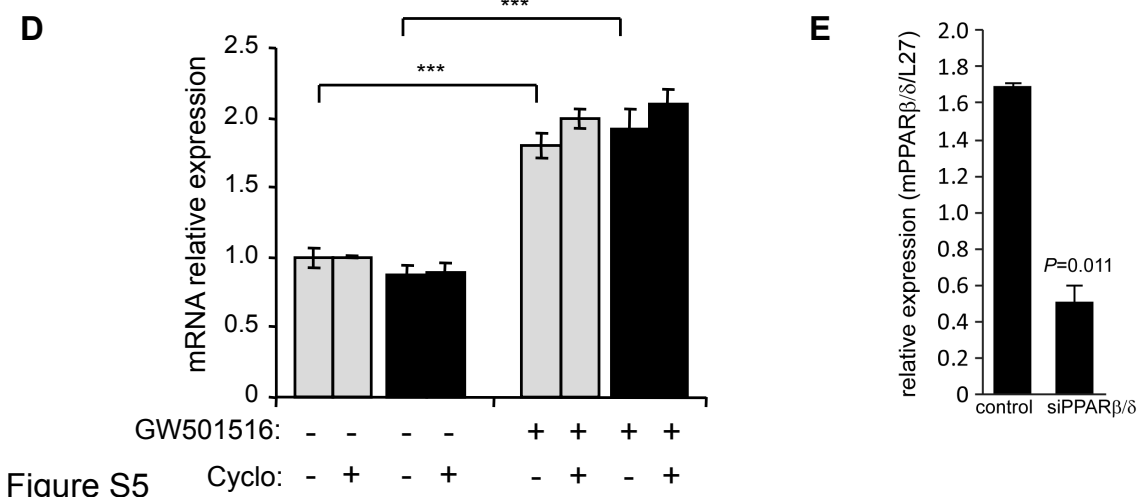

Figure S5

Supplement: Supplementary file 6 [file emmm0006-0080-sd6.pdf]

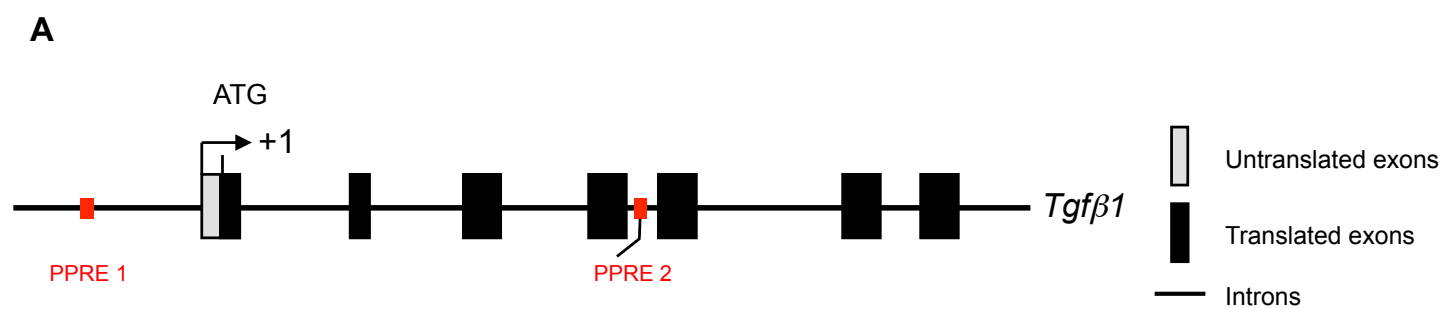

PPRE 1 (-3027) 5' TCACCTcTGTCTCCT 3'

PPRE 2 (+10201) 5' CGGTCAgAGGACA 3'

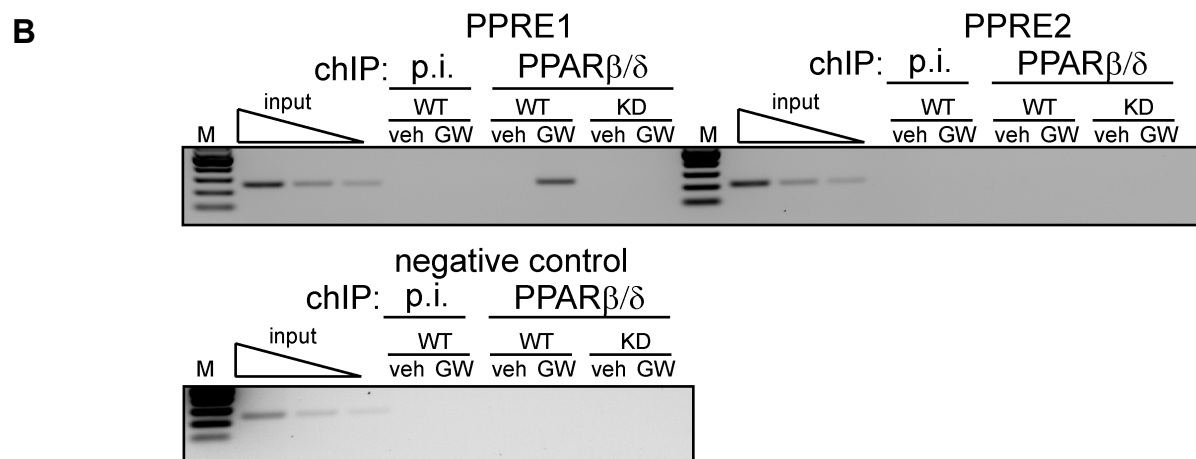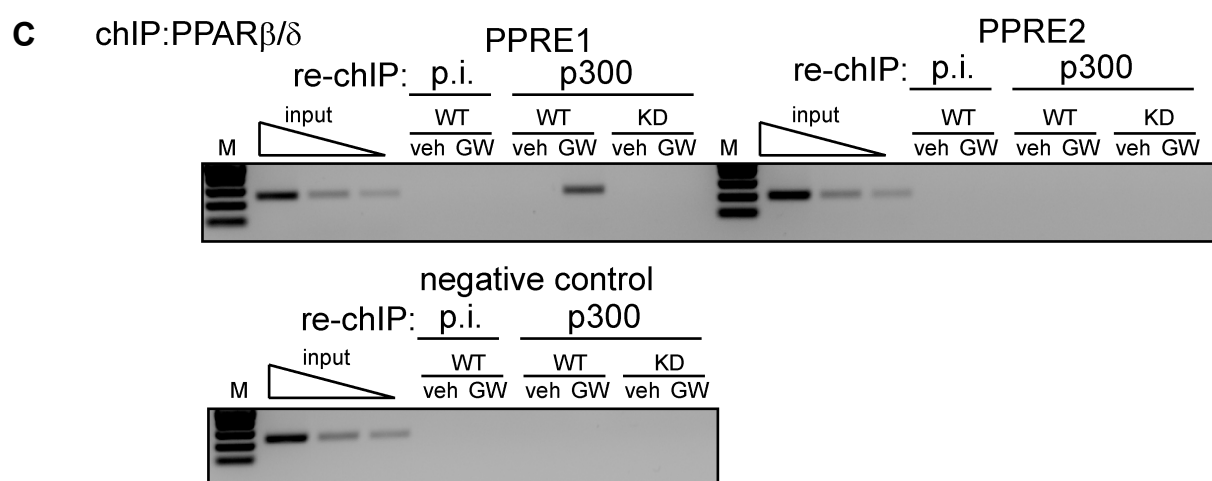

Figure S6

Supplement: Supplementary file 7 [file emmm0006-0080-sd7.pdf]

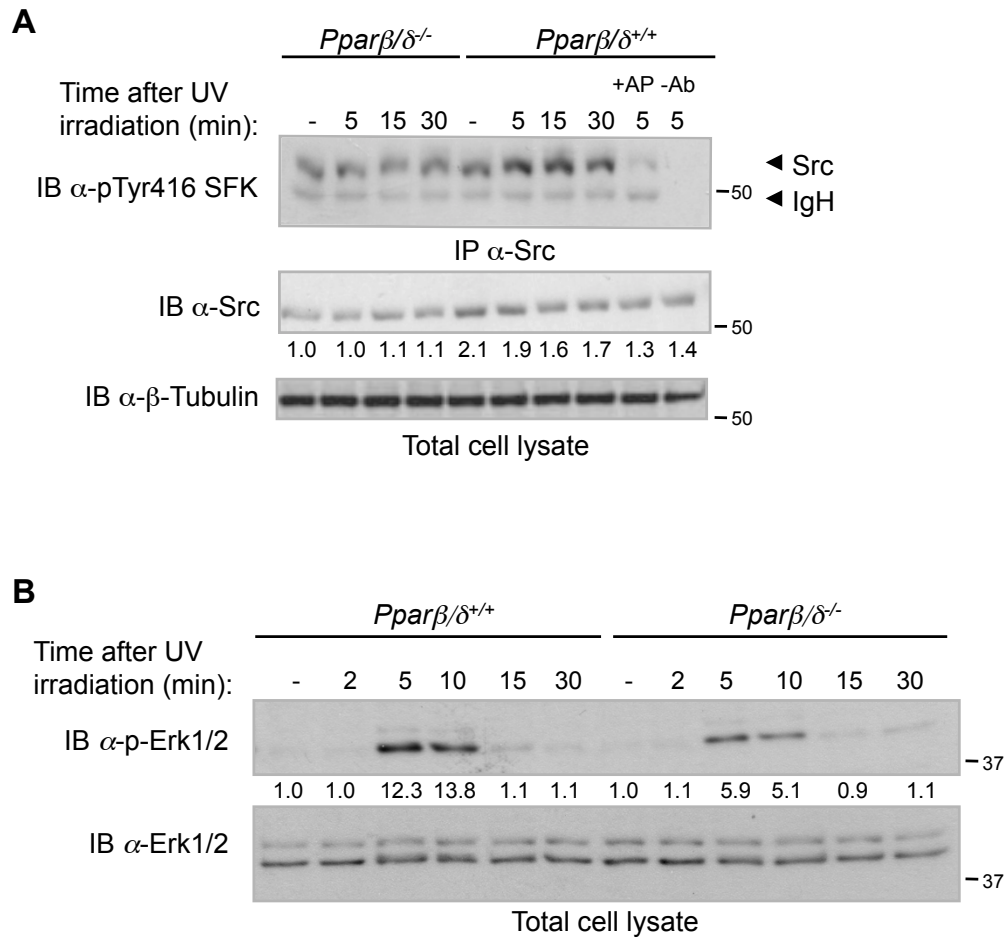

Figure S7

Supplement: Supplementary file 8 [file emmm0006-0080-sd8.pdf]

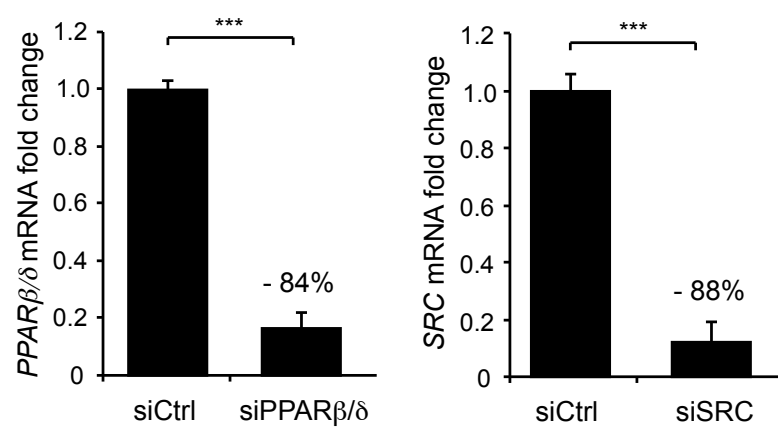

Figure S8

Supplement: Supplementary file 9 [file emmm0006-0080-sd9.pdf]

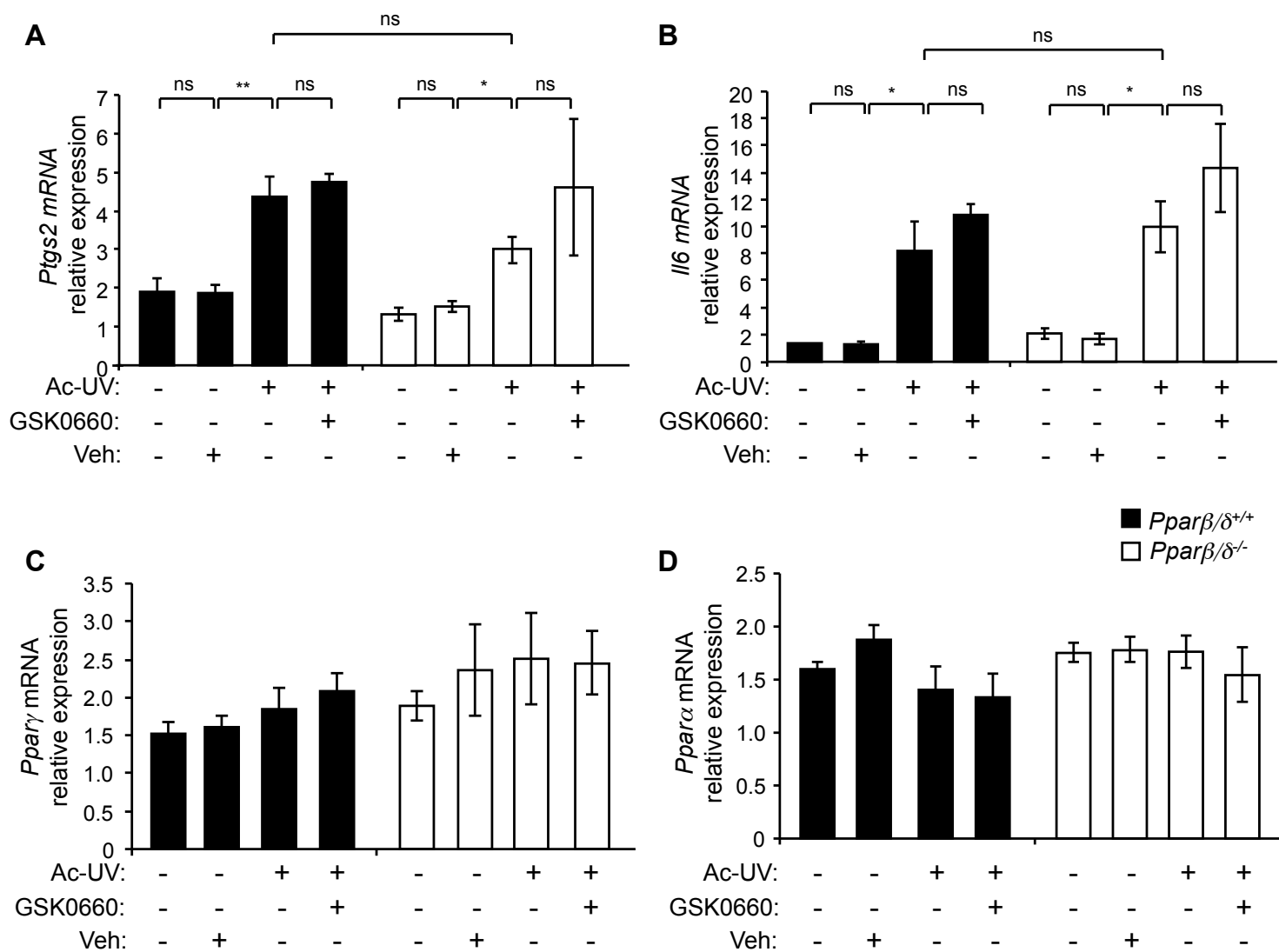

Figure S9

Supplement: Supplementary file 10 [file emmm0006-0080-sd10.pdf]

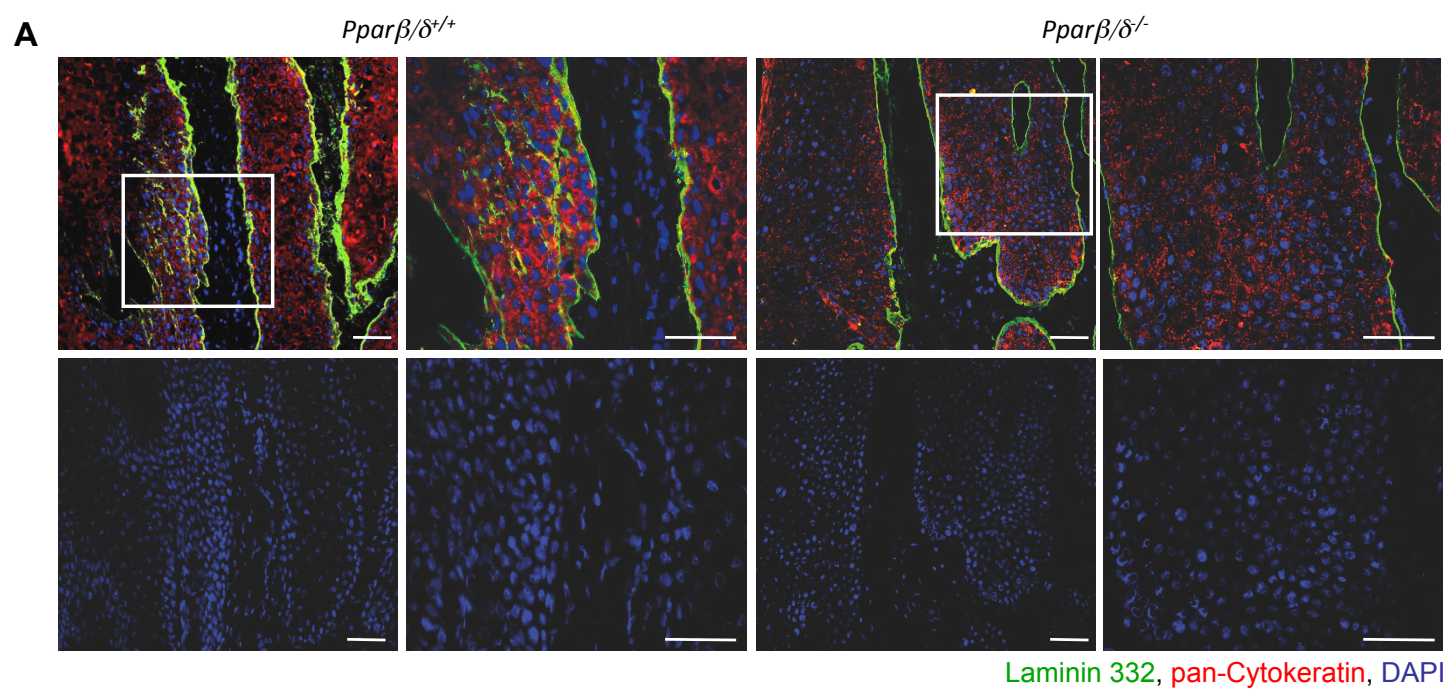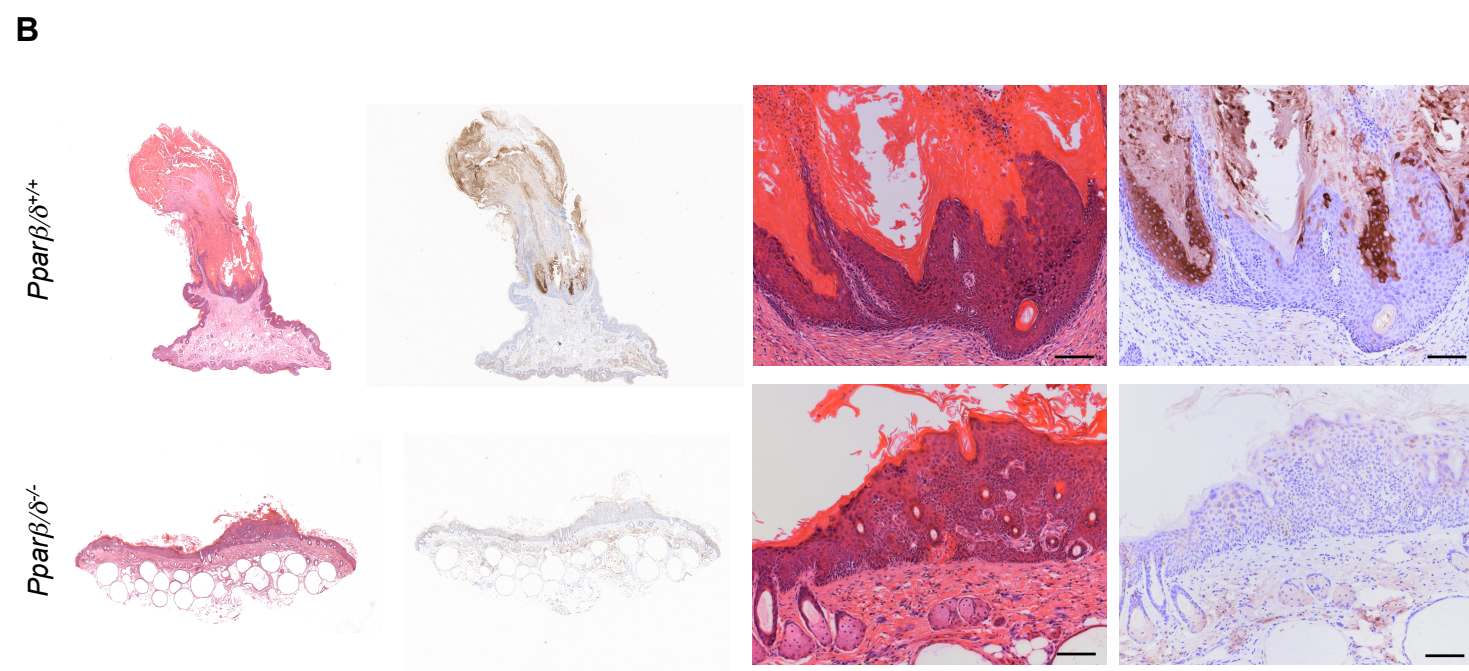

Figure S10

Supplement: Supplementary file 11 [file emmm0006-0080-sd11.pdf]

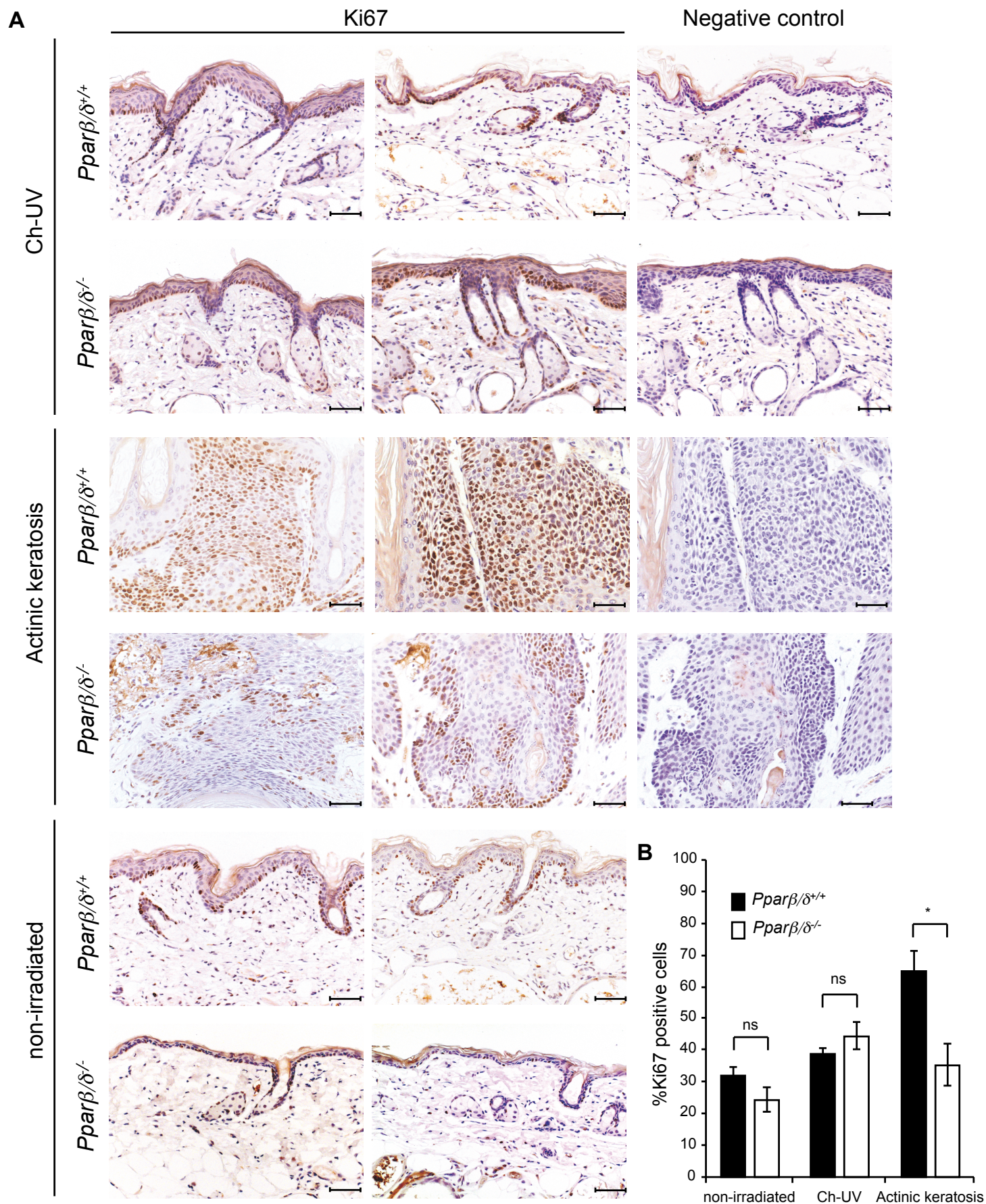

Figure S11

Supplement: Supplementary file 12 [file emmm0006-0080-sd12.pdf]

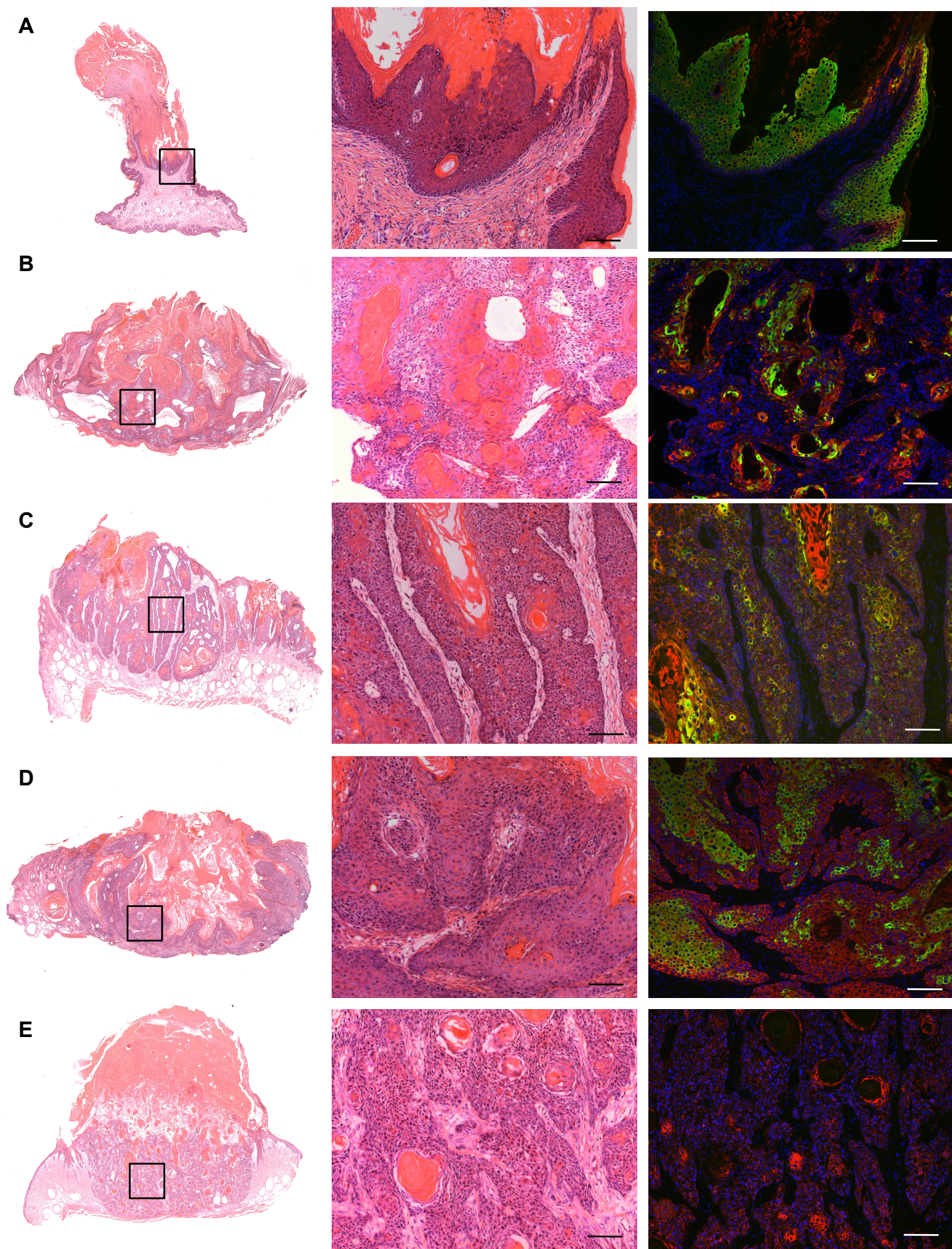

Figure S12

K10, K14, DAPI

Supplement: Supplementary file 13 [file emmm0006-0080-sd13.pdf]

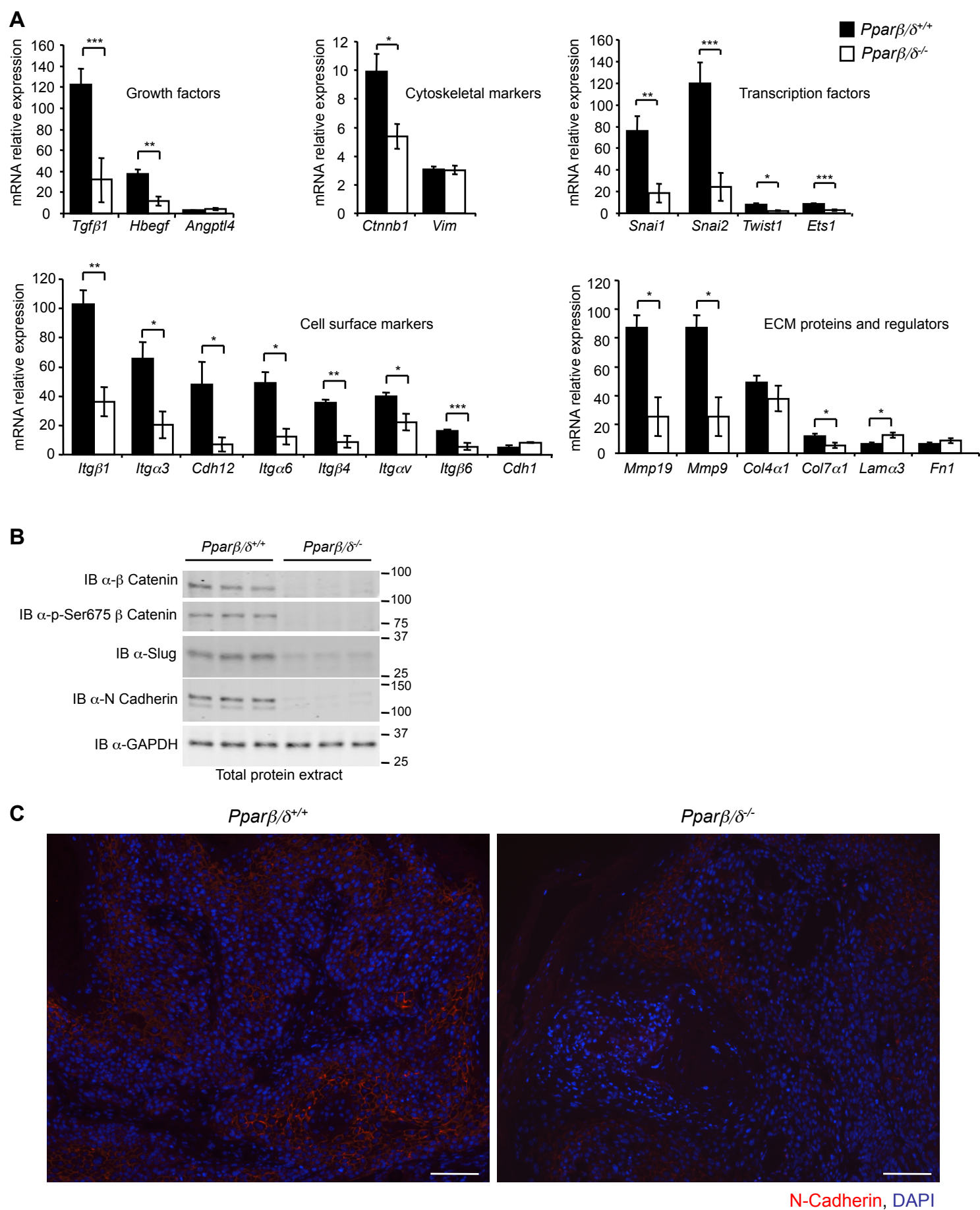

Figure S13

Supplement: Supplementary file 14 [file emmm0006-0080-sd14.pdf]

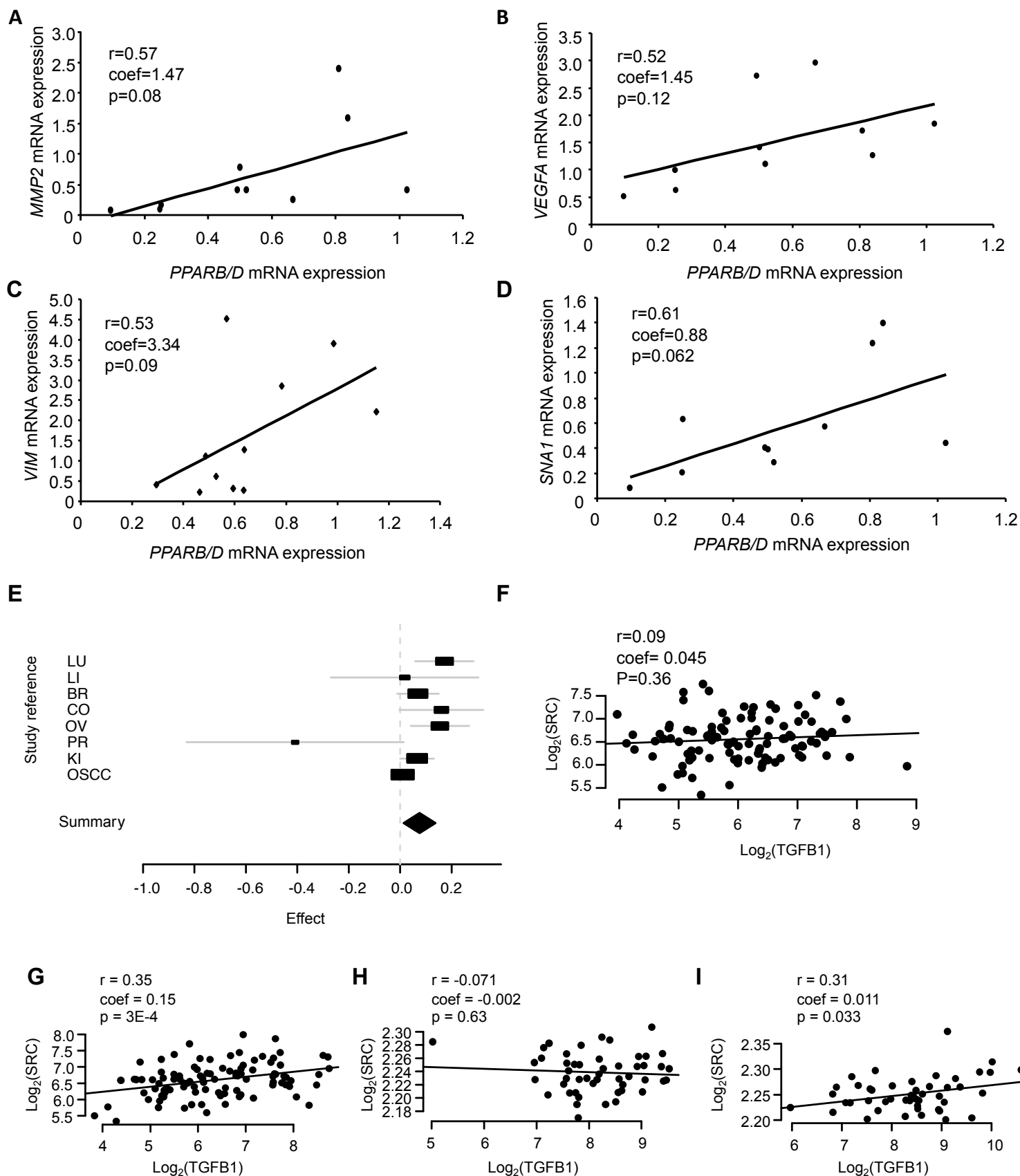

Figure S14

Supplement: Supplementary file 15 [file emmm0006-0080-sd15.pdf]
