# Supplementary material for: Src is activated by the nuclear receptor peroxisome proliferator-activated receptor β/δ in ultraviolet radiation-induced skin cancer
Source: EMBO Mol Med. 2013 Nov 6;6(1):80–98. doi: 10.1002/emmm.201302666 (PMC3936491; doi:10.1002/emmm.201302666)
Supplement: Supplementary file 16 [file emmm0006-0080-sd16.pdf]

Table S1

Primer sequences (5’–3’) used for Src-PPRE subcloning from the Balb/c mouse keratinocyte cell line genome in which digestion sites for Nhe1 in the forward primer and HindIII in the reverse primer were introduced

mSrc-PPRE1-F Nhe1	GAAGGGCTAGCCAGTGGGAACCCAGGAAGTA
mSrc-PPRE1-R HindIII	GAAGGAAGCTTGGCTCATAACAAGCTCACAGG
mSrc-PPRE2-F NheI	GAAGGGCTAGCCCCACCATTTCTGGTTCATT
mSrc-PPRE2-R HindIII	GAAGGAAGCTTATGAGTGCAAGAAGGCCACT
mSrc-PPRE3-F NheI	GAAGGGCTAGCTCTCCAGCCTCCATCCATAC
mSrc-PPRE3-R HindIII	GAAGGAAGCTTTAGGCAGGCCTCAGTTTACC
mSrc-PPRE4-F NheI	GAAGGGCTAGCCAGGTTGTGGCTTCTCCATT
mSrc-PPRE4-R HindIII	GAAGGAAGCTTTTCCATCTCCAACACCACAG
mSrc-PPRE5-FNheI	GAAGGGCTAGCTGGCACATCCAGAACACATT
mSrc-PPRE5-R HindIII	GAAGGAAGCTTGTGGGGTGTGGACATGAAA

Primer sequences (5’–3’) used for introducing mutations in pGL4-Src-PPRE constructs (mutation sites are underlined)

PPRE1mut-F	GCTCCTGACTGGGCCGAGAAATGAAGCATGCCTTCTGAGGAGCCGC
PPRE1mut-R	GCGGCTCCTCGAAGGCATGCTTCATTTCTCGGCCCAGTCAGGAGC
PPRE2mut-F	CATGGCATGTAAGTGAAGATATGAAGAATGCTTTTGGGAGTTAG
PPRE2mut-R	CTAACTCCCAAAAGCATTCTTCATATCTTCACTTACATGCCATG
PPRE3mut-F	GGTGAGAGCAATCAGAGAGATATGAAGTATGGGCAGGTCTTCCG
PPRE3mut-R	CGGAAGACCTGCCCATACTTCATATCTCTCTGATTGCTCTCACC
PPRE4mut-F	CCTCCGTTCTCTGTAAGAGATGAATTATTCAGAAACCAATTCTTC
PPRE4mut-R	GAAGAATTGGTTTCTGAATAATTCATCTCTTACAGAGAACGGAGG
PPRE5mut-F	CAGGATCTTTGGGAAGATATAAGAGATTTTTGGCGGGATCCTGA
PPRE5mut-R	TCAGGATCCCGCCAAAAATCTCTTATATCTTCCCAAAGATCCTG
