# Supplementary material for: Src is activated by the nuclear receptor peroxisome proliferator-activated receptor β/δ in ultraviolet radiation-induced skin cancer
Source: EMBO Mol Med. 2013 Nov 6;6(1):80–98. doi: 10.1002/emmm.201302666 (PMC3936491; doi:10.1002/emmm.201302666)
Supplement: Supplementary file 17 [file emmm0006-0080-sd17.pdf]

Table S2. Primer sequences used in qPCR and 5' RACE experiments.

Primers used for real-time RT-qPCR (m, murin; h, human)

**Gene**	**Forward** (5'-3')	**Reverse** (5'-3')
mPPARα	CCTGTTTGTGGCTGCTATAATTTG	GGAAGAGGAAGGTGTCATCTGG
mPPARβ/δ	CGGCAGCCTCAACATGG	AGATCCGATCGCACTTCTCATAC
hPPARβ/δ	GCATGAAGCTGGAGTACGAGAAG	GCATCCGACCAAAACGGATA
mPPARγ1	TGATTACAAATATGACCTGAAGC	TTGTAGAGCTGGGTCTTTTCAGAAT
mPlin2	GACCTTGTGTCCTCCGCTTAT	CAACCGCAATTTGTGGCTC
mEef1a1	CCTGGCAAGCCCATGTGT	TCATGTCACGAACAGCAAAGC
mRps9	GACCAGGAGCTAAAGTTGATTGGA	TCTTGGCCAGGGTAAACTTGA
hRPL27	TGTCCTGGCTGGACGCTACT	CTGAGGTGCCATCATCAATGTT
hHPRT	TGACACTGGCAAAACAATGCA	GGTCCTTTTCACCAGCAAGC
Krt13	GCCAGCTACCTGGATAAGGTG	CAGATGCCAGTCACGAATCTTC

The following primers were purchased from Qiagen (QuantiTect primer assay):

mSrc	QT02266831	mYes	QT01061116
mFyn	QT00176666	mTgfβ1	QT00145250
mEts1	QT01070923	mPtgs2	QT00165347
mIl6	QT00098875	mHbegf	QT00158452
mVim	QT00159670	mCtnnβ1	QT00160958
mSnai1	QT00240940	mSnai2	QT00098273
mGsc	QT00095221	mFoxc2	QT00252175
mTwist1	QT00097223	mItgαv	QT00095235
mItgα6	QT00144354	mItgβ1	QT00155855
mItgβ6	QT00128233	mCdh12	QT01065526
mItgα3	QT00125678	mCdh1	QT00121163
mItgβ4	QT01065729	mCol7α1	QT00105546
mMmp19	QT00138089	mLamα3	QT01889104
mMmp2	QT00116116	mMmp9	QT00108815
mFn1	QT00135758	mCol5α1	QT02324987
mCol4α1	QT00100128	mKrt10	QT01748397
mKrt14	QT00114422	hSRC	QT00039326

hYES	QT00037940	hFYN	QT00054005
hANGPTL4	QT00003661	hPLIN2	QTOOOO1911
hMMP19	QT00027286	hMMP2	QT00088396
hVEGFa	QT01682072	hSNAI1	QT00010010
hVIM	QT00095795		

PPAR: peroxisome proliferator–activated receptor; Plin2: Perilipin 2; Eef1a1: eukaryotic translation elongation factor 1 alpha 1; Rps9: ribosomal protein S9; RPL27: ribosomal protein L27; HPRT: hypoxanthine phosphoribosyltransferase 1; Src: Rous sarcoma oncogene; Yes: Yamaguchi sarcoma viral (v-yes) oncogene homolog 1; Fyn: Fyn proto-oncogene; Tgfβ1: transforming growth factor, beta 1; Ets1: E26 avian leukemia oncogene 1, 5' domain; Ptgs2: prostaglandin-endoperoxide synthase 2; Il6: interleukin 6; Hbegf: heparin-binding EGF-like growth factor; Vim: vimentin; Ctnnβ1: catenin (cadherin associated protein), beta 1; Snai1: snail homolog 1 (Drosophila); Snai2: snail homolog 2 (Drosophila); Gsc: goosecoid homeobox; Foxc2: forkhead box C2; Twist1: twist homolog 1 (Drosophila); Itgαv: integrin alpha V; Itgα6: integrin alpha 6; Itgβ1: integrin beta 1 (fibronectin receptor beta); Itgβ6: integrin beta 6; Cdh12: cadherin 12; Itgα3: integrin alpha 3; Cdh1: cadherin 1; Itgβ4: integrin beta 4; Col7α1: collagen, type VII, alpha 1; Mmp19: matrix metallopeptidase 19; Lama3: laminin, alpha 3; Mmp2: matrix metallopeptidase 2; Mmp9: matrix metallopeptidase 9; Fn1: fibronectin 1; Col5α1: collagen, type V, alpha 1; Col4α1: collagen, type IV, alpha 1; Krt10: keratin 10; Krt 13: keratin 13; Krt14: keratin 14; SRC: v-src sarcoma (Schmidt-Ruppin A-2) viral oncogene homolog; YES: v-yes-1 Yamaguchi sarcoma viral oncogene homolog 1; FYN: FYN oncogene; ANGPTL4: angiopoietin-like 4; PLIN2: perilipin 2; MMP19: matrix metallopeptidase 19; MMP2: matrix metallopeptidase 2; VEGFa: vascular endothelial growth factor A; SNAI1: snail homolog 1 (Drosophila); VIM: vimentin.

Primers used for 5’ RACE

**Primer**	**Forward** (5’-3’)
GSP1	GCGGGAGGTGATGTAGAAAC
GSP2	GGTCTCACTCTCCCTCACGA
GSP3	GAGTTGAAGCCTCCGAAGAG
Frohman	CAGTGAGCAGAGTGACGAGGACTCGAGCTCAAGCTTTTTTTTTTTTTTN
Qo	CAGTGAGCAGAGTGACG

GSP: gene-specific primer
