# Supplementary material for: Src is activated by the nuclear receptor peroxisome proliferator-activated receptor β/δ in ultraviolet radiation-induced skin cancer
Source: EMBO Mol Med. 2013 Nov 6;6(1):80–98. doi: 10.1002/emmm.201302666 (PMC3936491; doi:10.1002/emmm.201302666)
Supplement: Supplementary file 18 [file emmm0006-0080-sd18.pdf]

Table S3. Primer sequences used in ChIP experiments

Primer sequences used in ChIP experiments for *Src* PPREs

	**Forward (5' -> 3')**	**Reverse (5' -> 3')**
PPRE 1	CAGGAGTGTGCATGTGCGTG	TTGCAGGGCTCATAACAAGCTC
PPRE 2	GGTGTGCCCACCATTTCTGG	GAGCTCGTGAGATGGATGGCTC
PPRE 3	GCCTCCATCCATACAGACGGTG	GGGCAGTGCTATTTGCAAGGTG
PPRE 4	GCCCTTCCGACCCTTCTTGCAC	ATGAGCCCTCAGTTCCATCTCC
PPRE 5	TGGCACATCCAGAACACATTCC	ATCCGCACCTGCAGAGATGC
Negative Control	GGGAAGATGACTGCTCTGTGCC	ACAGACCAAGGCCGCTGATG

Primer sequences used in ChIP experiments for *Tgfβ1* PPREs and PCR conditions

	**Forward (5' -> 3')**	**Reverse (5' -> 3')**
PPRE 1	TGCAGGCCTCCTATCGCTCAAG	CATCCTGGTTGGCTTGTTTGCT
PPRE 2	AGGGCCATTGTGTGTGTGTGTC	GTTCGAGACAGGGCTTTGCTGT
Negative	TGGTCTTTGACGGAGGCTTCTG	CACATCGCCAGCTGCAGTCAT

<table>
<tr><td>95.0^{o}C</td><td>3 min</td><td></td></tr>
<tr><td>95.0^{o}C</td><td>30 s</td><td rowspan="2">30 cycles</td></tr>
<tr><td>60.0^{o}C (PPRE 1 and Negative)

56.9^{o}C (PPRE 2)</td><td>30 s</td></tr>
<tr><td>72.0^{o}C</td><td>30 s</td><td></td></tr>
</table>
