# Supplementary material for: Src is activated by the nuclear receptor peroxisome proliferator-activated receptor β/δ in ultraviolet radiation-induced skin cancer
Source: EMBO Mol Med. 2013 Nov 6;6(1):80–98. doi: 10.1002/emmm.201302666 (PMC3936491; doi:10.1002/emmm.201302666)
Supplement: Supplementary file 19 [file emmm0006-0080-sd19.pdf]

Supporting Information Material and Methods

Mouse primary keratinocyte cultures. Cultures from mouse skin keratinocytes were established from neonates at 1 to 3 days of age as previously described (Di-Poi et al, 2005), except that medium was replaced with fresh complete medium 3 h after seeding to allow selection of proliferating keratinocytes (Hakkinen et al, 2001).

HaCaT UVB GW501516 treatment. HaCaT cells were cultured routinely in Dulbecco's Modified Eagle Medium containing 10% fetal calf serum (Invitrogen) at 37°C in a 5% CO_2 atmosphere and 95% humidity. Before the experiment, sub-confluent cells were starved in serum-free Dulbecco's Modified Eagle Medium overnight. Cells were treated for 24 h with GW501516 (10 μM) or vehicle (DMSO: 1/1000) or 4 h in the presence or absence of cycloheximide (1 μg/mL) and then processed for RNA extraction.

HaCaT UVB irradiation. HaCaT cells were cultured routinely in Dulbecco's Modified Eagle Medium containing 10% fetal calf serum (Invitrogen) at 37°C in a 5% CO_2 atmosphere and 95% humidity. Before the experiment, sub-confluent cells were starved in serum-free Dulbecco's Modified Eagle Medium overnight. For siRNA experiments, 25 nM of ON-TARGETplus SMART-pool siRNA targeting PPARβ/δ or Src (Dharmacon L-003435 and L-003175, respectively) were transfected using INTERFERin reagent (Polyplus-transfection) for 24 h. Knockdown efficiency was tested by real-time PCR 48 h later (Supplemental Fig 5). For the PP2 (Calbiochem) experiment, 10 μM of the inhibitor was added 30 min before UVB exposure. For UVB irradiation, cells were irradiated in pre-heated phosphate-buffered saline (37°C) at 40 mJ/cm^2 using the UV RMX3W system (312 nm) from BioSun (Vilber Lourmat) and harvested 30 min later.

Total RNA isolation, RT-PCR, and real-time PCR. For skin and cells, total RNA was extracted with TRIzol (Invitrogen). One microgram of whole RNA was reverse transcribed using

random hexamers and MMLV reverse transcriptase (Invitrogen). The cDNA equivalent of 10 ng of total RNA was amplified by PCR in a 4500 Fast Real-Time PCR System (Applied Biosystems). Primers sequences are given in Supplementary Table 2.
