# Supplementary material for: Src is activated by the nuclear receptor peroxisome proliferator-activated receptor β/δ in ultraviolet radiation-induced skin cancer
Source: EMBO Mol Med. 2013 Nov 6;6(1):80–98. doi: 10.1002/emmm.201302666 (PMC3936491; doi:10.1002/emmm.201302666)
Supplement: Supplementary file 20 [file emmm0006-0080-sd20.pdf]

Supplementary Figures

Figure S1. PPARβ/δ is specifically up-regulated in skin upon chronic UV exposure. Real-time RT-PCR showing *Pparα*, *Pparβ/δ*, and *Pparγ* expression in dorsal skin of SKH-1 hairless wild-type mice non-irradiated (control) or irradiated for 1, 5, and 10 weeks and in papillomas at 17 weeks. Results represent means ± SEM for 15 mice. The value from control skin is set to 1. Statistical analyses compared each PPAR isotype to control skin for each time point; *P*-values are, from top to bottom and from left to right, **p* = 0.035, ***p* = 0.025, ****p* = 0.001, ****p* = 0.0008, **p* = 0.04, **p* = 0.025, **p* = 0.015 by two-tailed Student's *t*-test; ns, not significant.

Figure S2. PPARβ/δ is specifically up-regulated and activated in skin upon acute UV exposure. **A**. Quantification of *Ptgs2* (*Cox2*) (left) and *Il6* (right) mRNA levels by real-time RT-PCR in whole dorsal skin of $Ppar\beta/\delta^{+/+}$ and $Ppar\beta/\delta^{-/-}$ mice subjected (+) or not (-) to acute UV (Ac-UV) radiation. Results represent means ± SEM for 12 $Ppar\beta/\delta^{+/+}$ and 12 $Ppar\beta/\delta^{-/-}$ mice and are representative of three independent experiments; *P*-values are **p* = 0.033 (*Ptgs2*), 0.016 (*Il6*) calculated by two-tailed Student's *t*-test; ns, not significant. **B, C**. Measurement of *Pparβ/δ* (left), *Tgfβ1* (middle), and *Plin2* (right) (**B**) and *Src* (**C**) gene expression by RT-PCR in whole dorsal skin of $Ppar\beta/\delta^{+/+}$ and $Ppar\beta/\delta^{-/-}$ mice 24 h after acute UV (Ac-UV) irradiation. Values from non-irradiated dorsal skin of $Ppar\beta/\delta^{+/+}$ mice (**B**, left) and from Ac-UV dorsal skin of $Ppar\beta/\delta^{-/-}$ mice (**B**, middle and right, **C**) were set to 1. Means ± SEM for 12 $Ppar\beta/\delta^{+/+}$ and 12 $\text{Ppar}\beta/\delta^{-/-}$ mice are presented. Data are representative of three independent experiments; *P*-values are ****p* = 0.0004 for *Pparβ/δ*; **p* = 0.0176 and ****p* = 0.0035 for *Tgfβ1*; **p* = 0.0242 and ****p* = 0.0044 for *Plin2*; **p* = 0.025 and ***p* = 0.0056 for *Src* calculated by two-tailed Student's *t*-test; ns, not significant. **D**. Measurement of dermis (Collagen 4α1 (*Col4α1*), left) and epidermis (Keratins 10 (*Krt10*) and 14 (*Krt14*), middle and right) markers in dorsal skin of $Ppar\beta/\delta^{+/+}$ mice acutely irradiated or not with UV after epidermis from dermis separation. Results represent means ± SEM for n = 5 $Ppar\beta/\delta^{+/+}$; *P*-values are, from top to bottom and from left to right, ****p* = 0.0001, ***p* = 0.0091, ****p* = 0.0025, ****p* = 0.0008 for *Col4α1*; ****p* = 0.0009, ****p* = 0.0007, ****p* = 0.0007, ****p* = 0.0015 for *Krt10*; ****p* = 0.0006, ****p* = 0.0004, ****p* = 0.0003, ****p* = 0.0025 for *Krt14* calculated by two-tailed Student's *t*-test; ns, not significant.

Figure S3. PPARβ/δ drives Src mRNA and protein expression, but not Fyn and Yes mRNA expression. A. Left, *Src* mRNA expression level in cultures of primary keratinocytes from *Pparβ/δ*$^{+/+}$ and *Pparβ/δ*$^{-/-}$ newborn pups; **p* = 0.025, *t*-test. Middle, quantification of Src protein levels obtained *via* immunoblot (right) normalized to β-tubulin expression (used as a loading control). Right, immunoblot of Src protein expression in cultures of primary keratinocytes from *Pparβ/δ*$^{+/+}$ and *Pparβ/δ*$^{-/-}$ newborn pups. For all three panels, results are means ± SD (n = 4) and are representative of three independent experiments; ***p* = 0.009, *t*-test. **B**. Fyn and Yes mRNA levels were evaluated by real-time RT-PCR in dorsal skin of *Pparβ/δ*$^{+/+}$ and *Pparβ/δ*$^{-/-}$ mice subjected (+) or not (-) to an acute UV radiation (Ac-UV). Results represent means ± SEM of 12 *Pparβ/δ*$^{+/+}$ and 12 *Pparβ/δ*$^{-/-}$ mice. **C**. Fyn and Yes mRNA levels in chronically irradiated (Ch-UV) non-tumorigenic dorsal skin of *Pparβ/δ*$^{+/+}$ and *Pparβ/δ*$^{-/-}$ mice. Results show means ± SEM of 13 *Pparβ/δ*$^{+/+}$ and 9 *Pparβ/δ*$^{-/-}$ mice; ns, not significant.

Figure S4. **PPARβ/δ promotes Src protein expression in acutely irradiated skin.** Representative pictures of Src immunohistochemistry in non-irradiated (top) and acutely UV-irradiated (Ac-UV) (middle and bottom) skin from the analysis of 12 *Pparβ/δ*$^{+/+}$ and 12 *Pparβ/δ*$^{-/-}$ mice. Scale bars, 50 μm.

Figure S5. Activated PPARβ/δ promotes Src expression in a human keratinocyte cell line. A. Measurement of SRC and ANGPTL4 mRNA levels in HaCaT cells treated for 24 h with vehicle (DMSO; -) or the PPARβ/δ agonist GW501516 (10 μM; +). Results show means ± SD (n = 3) and are representative of three independent experiments. *P*-values are, from left to right, ****p* = 0.0032, 0.0007 calculated by *t*-test. **B**. Left, quantification of Src protein expression obtained *via* immunoblot (right) normalized to GAPDH expression (used as a loading control). Results depict means ± SD (n = 3) and are representative of three independent experiments; ***p* = 0.0056, *t*-test. Right, immunoblot of Src expression in HaCaT cells treated for 24 h with vehicle (DMSO) or GW501516 (10 μM). **C**. Schematic of the locations of peroxisome proliferator response elements (PPREs, red) in the *Src*-001 regulatory region. The sequences of the five wild-type (wt) and mutant (mut) PPREs that were tested in the transfection assay (Fig 3C) are indicated. **D**. Measurement of SRC and ANGPTL4 mRNA levels in HaCaT cells treated for 4 h with vehicle (DMSO; -) or the PPARβ/δ agonist GW501516 (10

μM; +) in the presence (+) or absence (-) of cycloheximide (1 μg/mL). Results show means ± SD (n = 3) and are representative of three independent experiments. *P*-values are, from top to bottom, ****p* = 0.0025, 0.0045 calculated by *t*-test. **E.** Measurement of Pparβ/δ mRNA levels in MK cells transfected by siRNA against mouse *Pparβ/δ* and scrambled siRNA (control).

Figure S6. *Tgfβ1* is a PPARβ/δ target gene in mouse keratinocytes. A. Schematic structure of murine Tgfβ1 gene and position and sequence of identified PPREs. **B, C.** Representative results of ChIP experiments using anti-PPARβ/δ antibody **(B)** followed by re-ChIP with anti-p300 **(C)** performed in mouse keratinocyte cells down-regulated for PPARβ/δ expression by siRNA (knockdown cells, KD) or not (wild-type cells, WT) treated or not with GW501516. The results show a PCR amplification of the PPRE 1 site. Preimmune serum (p.i.) served as a control for ChIP. Primer sequences are given in Supporting Information Table S3. Data are representative of n = 3 independent experiments.

Figure S7. PPARβ/δ-dependent up-regulation of Src expression enhances Src/Erk1/2 signaling in primary keratinocytes following UV exposure. Primary cultures of keratinocytes from *Pparβ/δ*$^{+/+}$ and *Pparβ/δ*$^{-/-}$ newborn pups were subjected to UV irradiation (40 mJ/cm^2) and harvested at the indicated time points. **A**. Src was immunoprecipitated (IP) from whole cell lysates and immunoblotted for p-Tyr416 Src family kinase (SFK). The total amounts of Src and β-tubulin (loading control) were analyzed by immunoblot of corresponding cell lysates. Data are representative of three independent experiments. **B**. Immunoblot detecting pErk1/2 and Erk1/2 from whole cell lysates. Data are representative of three independent experiments. IB: immunoblot.

Figure S8. Down-regulation of Pparβ/δ and Src expression by siRNA in the HaCaT cell line. Measurement of *PPARβ/δ* and *SRC* mRNA levels in the HaCaT cell line transfected by siRNA against *PPARβ/δ* (siPPARβ/δ) and *SRC* (siSRC) and corresponding control siRNAs (siCtrl) for 48 h. Results show means ± SD (n = 3) and are representative of three independent experiments; ****p* = 8.34.10^{-8} for *PPARβ/δ* and ***p = 0.0001 for *SRC* calculated by *t*-test.

Figure S9. *In vivo* effects of pharmacological inhibition of PPARβ/δ on the expression of inflammatory markers and PPARs. Dorsal skin of *Pparβ/δ*$^{+/+}$ and *Pparβ/δ*$^{-/-}$ mice was topically

treated with the PPARβ/δ antagonist GSK0660 or vehicle (Veh; 70% ethanol) prior to acute UV (Ac-UV) irradiation; mice were sacrificed 24 h later. Quantification of *Ptgs2* (**A**), *Il6* (**B**), *Pparγ* (**C**), and *Pparα* (**D**) levels by real-time RT-PCR. Means ± SEM are presented (n = 6 mice/genotype/group). Data are representative of two independent experiments; *P*-values are $*p = 0.016$, $**p = 0.010$ for *Ptgs2* and $*p = 0.037$, 0.012 from left to right for *Il6* calculated by two-tailed Student's *t*-test; ns, not significant.

Figure S10. Laminin 332 and Keratin 13 staining in samples of actinic keratosis with moderate atypia (grade II) from wild-type and *Pparβ/δ$^{-/-}$* mice. A. Representative immunofluorescence staining of laminin 332 (green). Pan-Cytokeratin, red; DAPI, blue. Scale bars, 50 µm. **B.** Representative immunohistochemistry of Keratin 13 staining. Scale bars, 100 µm.

Figure S11. Increased cell proliferation in wild-type versus *Pparβ/δ$^{-/-}$* actinic keratosis. A. Representative Ki67 staining comparing the number of proliferating cells in sections of chronically irradiated (Ch-UV) dorsal skin, actinic keratosis, and normal (non-irradiated) skin of *Pparβ/δ$^{+/+}$* and *Pparβ/δ$^{-/-}$* mice. Scale bar, 50 µm. **B**. Quantification (means ± SEM) of Ki67-positive cells in epidermis (n = 3); $*p = 0.035$ by *t*-test; ns, not significant.

Figure S12. Representative pictures of actinic keratosis with moderate and severe atypia (grades II and III) and squamous cell carcinoma (SCC) in wild-type SKH-1 hairless mice. A–E. Global morphology and representative H/E staining and Keratin 10/Keratin 14 immunoflorescence staining of actinic keratosis grade II **(A)**, grade III **(B)**, grade I SCC **(C)**, grade II SCC **(D)**, and grade III SCC **(E)**. Note that Keratin 10 is highly expressed by actinic keratoses of low histological grade, whereas Keratin 14 is very weakly expressed **(A)**. Following the progression of these lesions to higher histological grades of malignancy, the Keratin 10 gradually decreases. At the same time, the expression of Keratin 14 increases. Note the strong expression of Keratin 14 by SCC of intermediate and high grades and the reduction or absence of Keratin 10 expression in these lesions **(D** and **E)**. Scale bar, 100 µm.

Figure S13. Epithelial-to-mesenchymal transition marker expression in grade II SCC from wild-type and *Pparβ/δ$^{-/-}$* mice. A. mRNA expression of EMT transition markers in 15 grade II SCC

of *Pparβ/δ*$^{+/+}$ and *Pparβ/δ*$^{-/-}$ mice by real-time RT-PCR. Means ± SEM are presented; *P*-values are ***p = 0.0028 (*Tgfβ1*), **p = 0.0014 (*Hbegf*), *p = 0.034 (*Ctnnb1*), **p = 0.0056 (*Snai1*), ***p = 0.0035 (*Snai2*), *p = 0.0184 (*Twist1*), ***p = 0.0029 (*Ets1*), **p = 0.0144 (*Itgβ1*), *p = 0.0133 (*Itgα3*), *p = 0.0480 (*Cdh12*), *p = 0.0186 (*Itgα6*), **p = 0.0063 (*Itgβ4*), *p = 0.0124 (*Itgαv*), ***p = 0.0002 (*Itgβ6*), *p = 0.045 (*Mmp19*), *p = 0.045 (*Mmp9*), *p = 0.0326 (*Col7α1*) and *p = 0.0197 (*Lamα3*) calculated by two-tailed Student's *t*-test. The full gene names are given in Supporting Information Table S2. **B**. Immunoblot of total β-Catenin, p-Ser675 β-Catenin, Slug, and N-Cadherin in tumors from *Pparβ/δ*$^{+/+}$ and *Pparβ/δ*$^{-/-}$ mice. Results are representative of n = 8 grade II SCC/genotype. GAPDH was used as loading control; IB, immunoblot. **C.** Representative immunoflorescence staining of N-Cadherin in grade II SCC sections from *Pparβ/δ*$^{+/+}$ and *Pparβ/δ*$^{-/-}$ mice. DAPI, blue. Scale bars, 100 µm.

Figure S14. Correlations between the expressions of PPARβ/δ and EMT markers in human squamous cell carcinomas. A–D. Correlations between *PPARβ/δ* and *MMP2* (**A**), *VEGFA* (**B**), *VIM* (**C**), and *SNAI1* (**D**) expression measured *via* real-time RT-PCR from human squamous cell carcinoma biopsies (n = 9). *P*-values are calculated by two-tailed Student's *t*-test. **E.** Forest plot of the interaction coefficient β3 (estimate) and its 95% confidence interval for the linear model SRC ~ β0+ βTGFB1+β2PPARB/D+β3TGFB1: PPARB/D. The absence of an interaction occurs when the confidence interval overlaps zero. The summarized meta-analysis of the interaction coefficient was estimated using a random effect model. LU: Lung, LI: Liver: BR: breast, CO: colon; OV: ovarian; PR: prostate; KI: kidney; OSCC: oral squamous cell carcinoma. **F–I**. Interaction between *TGFB1* and *PPARB/D* underlies *SRC* expression levels. All samples were sorted by *PPARB/D* expression. Samples with either the highest (top 50% of the samples) or lowest (bottom 50% of the samples) *PPARB/D* levels were identified. (**F**) Least squares regression between *TGFB1* and *SRC* expression level in ovarian carcinoma samples with the lowest *PPARB/D* expression (N = 101). The black line represents the least square fit. The observed linear regression coefficient (0.045) was not significantly different from zero (Student p = 0.36). A correlation between both variables of 0.09 was observed. (**G**) Linear regression between *TGFB1* and *SRC* levels in the 101 ovarian tumor samples with the highest *PPARB/D* expression reveals a significant linear relationship. (**H**) Least squares regression between *TGFB1* and *SRC* expression level in oral SCC (OSCC) samples (N = 49) with the lowest *PPARB/D* expression. No significant linear dependency was observed. (**I**) Significant linear

dependency between *TGFB1* and *SRC* expression level in OSCC samples (N = 48) with the highest *PPARB/D* expression.
